# Molecular basis of the biogenesis of a protein organelle for ethanolamine utilization

**DOI:** 10.1126/sciadv.adx9774

**Published:** 2025-10-01

**Authors:** Mengru Yang, Oluwatobi Adegbite, Ping Chang, Jin Cheng, Yue Wang, Marie Held, Xiaojun Zhu, Yan Li, Gregory F. Dykes, Yu Chen, Natasha Savage, Yu-Zhong Zhang, Jun Gao, Jay C. D. Hinton, Lu-Yun Lian, Lu-Ning Liu

**Affiliations:** ^1^ Institute of Systems, Molecular and Integrative Biology, University of Liverpool, Crown Street, Liverpool L69 7ZB, UK.; ^2^ MOE Key Laboratory of Evolution and Marine Biodiversity, State Key Laboratory of Marine Food Processing and Safety Control & College of Marine Life Sciences, Ocean University of China, Qingdao 266003, China.; ^3^ Hubei Key Laboratory of Agricultural Bioinformatics, College of Informatics, Huazhong Agricultural University, Wuhan 430070, China.; ^4^ Centre for Cell Imaging, University of Liverpool, Crown Street, Liverpool L69 7ZB, UK.; ^5^ Institute of Infection, Veterinary & Ecological Sciences, University of Liverpool, Crown Street, Liverpool L69 7ZB, UK.

## Abstract

Many pathogenic bacteria use proteinaceous ethanolamine utilization microcompartments (Eut BMCs) to catabolize ethanolamine. This ability gives pathogens a competitive edge over commensal microbiota, which can drive virulence in the inflamed gut. Despite such a critical function, the molecular mechanisms underlying the synthesis of Eut BMCs remain elusive. We report a systematic study for dissecting the molecular basis underlying Eut BMC assembly in *Salmonella*. We determined the functions of individual constituent proteins in the structure and function of Eut BMCs and demonstrated that EutQ is essential for cargo encapsulation and Eut BMC formation through specific association with the shell and cargo enzymes. We found that Eut proteins can self-assemble to form cargo and shell aggregates independently in vivo and that the biogenesis of Eut BMCs follows a “shell-initiated” pathway. Cargo enzymes exhibit dynamic liquid-like organization within the Eut BMC. Our findings provide mechanistic insights into the structure and assembly of the Eut BMC that serves as a paradigm for membraneless organelles.

## Teaser

EutQ governs enzymatic cargo encapsulation within the protein shell to drive Eut microcompartment biogenesis in *Salmonella*.

## INTRODUCTION

Ethanolamine (EA) is an abundant nutrient in the mammalian gastrointestinal (GI) tract, a membrane-rich environment due to the constant turnover of the mucosal epithelium and the resident microbiota (*1*–*3*). Diverse gut bacteria, including those from pathogenic genera such as *Salmonella*, *Clostridium*, *Klebsiella*, *Listeria*, *Escherichia*, and *Enterococcus*, can use EA as a sole source of carbon and/or nitrogen (*4*–*6*). The ability to metabolize EA provides a competitive growth advantage to pathogens over commensal microbiota. Moreover, EA plays a role in regulating the virulence of these pathogens (*7*, *8*) and EA catabolism is closely associated with virulence in the inflamed gut (*9*–*13*).

**Fig. 1.**
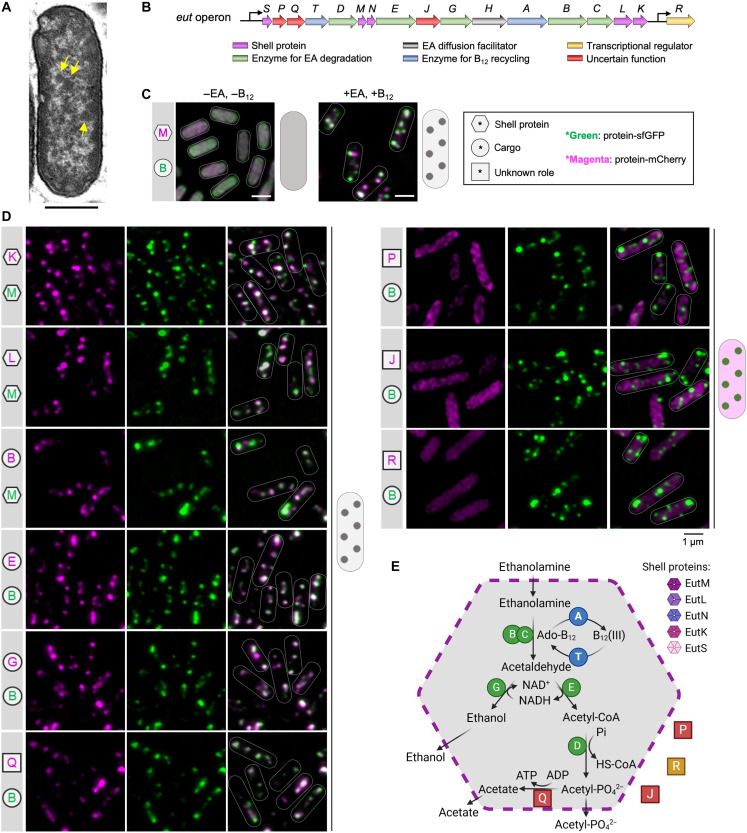
Eut BMC structure, gene operon, and protein organization in *S.* **Typhimurium** LT2. (**A**) Thin-section transmission electron microscopy of the *S.* Typhimurium LT2 WT strain following growth in the presence of EA and B_12_, showing the formation of Eut BMCs (yellow arrows). Scale bar, 500 nm. (**B**) The chromosomal *eut* operon of *S.* Typhimurium LT2 includes structural genes (*eutSMNLK*), catalytic genes (*eutDEGBC* for EA degradation and the *eutTA* for B_12_ recycling), *eutR* (transcriptional regulator), *eutH* (EA diffusion facilitator), and unknown function genes (*eutPQJ*), which are indicated in distinct colors as show in (E). (**C**) Fluorescence images show the *S.* Typhimurium LT2 cells carrying pBAD-*eutM-mCherry*::*eutB-sfGFP* grown in minimal medium, in the absence or presence of EA and B_12_. Hexagon represents shell proteins; circle represents cargo; square represents proteins with an unknown role and EutR. Eut protein in green represents tagging with sfGFP, whereas that in magenta represents tagging with mCherry. Gray color represents that the mCherry and sfGFP signals are colocalized. Scale bars, 1 μm. (**D**) Localization of Eut proteins in the cells grown in minimal medium in the presence of EA and B_12_. (**E**) Schematic model of the protein organization of the Eut BMC. The color scheme is the same as shown in (B), indicating the different organization of individual Eut proteins.

In many pathogenic species, such as *Salmonella enterica* serovar Typhimurium (*S.* Typhimurium), EA utilization is mediated by a group of proteins that are encoded by 17 genes clustered in the *eut* operon (*14*–*16*). These proteins, known as EutS/P/Q/T/D/M/N/E/J/G/H/A/B/C/L/K/R, include catalytic and structural protein components that self-assemble to form a polyhedral organelle, called the EA utilization microcompartment (Eut BMCs; Fig. 1, A and B). The Eut BMCs belong to a family of bacterial microcompartments (BMCs) that are widespread in the bacterial kingdom, which perform a variety of functions, including CO_2_ fixation, infection, and microbial ecology (*16*–*21*). However, despite their important functions in bacterial pathogenesis and host-pathogen interactions, our understanding of how the various shell proteins and cargo enzymes interact and self-assemble in vivo to form functional Eut BMCs is still limited.

Here, we perform a systematic study on the molecular mechanisms that underlie the organization and biogenesis of Eut BMCs in *S.* Typhimurium strain LT2. Using genetic modification, super-resolution fluorescence imaging, electron microscopy (EM), and growth assays, we delineate the roles of individual Eut proteins in Eut BMC assembly and functionality. Combining nuclear magnetic resonance (NMR) spectroscopy, AlphaFold predictions (*22*), and biochemical studies, we elucidate how the N-terminal domain of EutQ mediates the physical binding between the shell and cargos to ensure encapsulation. We found that Eut proteins independently self-assemble to form cargo and shell aggregates. Our results establish that the in vivo biogenesis of Eut BMCs follows a distinct “shell-initiated” assembly pathway, in which the assembly of shells takes place before the assembly of cargos and at the cell poles. We further demonstrate that the Eut BMC cargo core has liquid-like features, highlighting the essential role of phase separation in constructing and maintaining self-assembling protein organelles in pathogenic bacteria. Our study addresses long-standing questions surrounding Eut BMC biogenesis and the self-assembly mechanisms of shell and cargos into functional protein organelles. These findings could guide the development of therapeutic interventions for diseases caused by pathogens.

## RESULTS

### Subcellular localization of Eut proteins and protein organization of the Eut BMC

To examine the subcellular location and assembly of Eut proteins in *S.* Typhimurium, we labeled target Eut proteins with fluorescent proteins using a pBAD/*Myc*-His vector with the arabinose-inducible P*araBAD* promoter (*23*) (Fig. 1C, fig. S1, and tables S1 and S2). For in vivo super-resolution fluorescence imaging, selected Eut shell or cargo proteins tagged with superfolder green fluorescent protein (sfGFP) or mCherry were expressed under the control of the P*araBAD* promoter, along with the expression of the endogenous *eut* operon, which was activated in the presence of EA and cobalamin (vitamin B_12_) (*24*).

In the absence of EA and B_12_, no endogenous Eut proteins were expressed in *S.* Typhimurium. In the absence of Eut BMC formation, the individual vector-expressed Eut proteins, including the shell proteins EutM/K/L, cargo proteins EutG/B, as well as EutQ/P/J and EutR, were dispersed throughout the cytoplasm (Fig. 1C and fig. S2). One exception is the cargo protein EutE, which formed an aggregate at the cell pole. In addition, the multiple enzyme complex EutBC (sfGFP was fused at the EutC C terminus) appeared to aggregate at a single pole of the cell (fig. S3). Both EutE and EutC have short peptides at their N termini, which act as endogenous encapsulation peptides (EPs) to target cargo enzymes into the Eut BMC (*25*, *26*). The observed polar aggregation of EutE and EutBC was likely mediated by their EPs, consistent with the findings from 1,2-propanediol utilization microcompartments (Pdu BMCs) (*23*).

Growth of *S.* Typhimurium in the presence of EA and B_12_ led to expression of endogenous Eut proteins and formation of Eut BMCs (Fig. 1A). Fluorescence imaging revealed that the Eut proteins (shell proteins EutM/K/L and cargo proteins EutE/G/B/Q) exhibited a patchy distribution, representing the characteristic localization pattern of Eut BMCs in vivo (Fig. 1, C and D). By contrast, EutP, EutJ, and EutR were evenly distributed throughout the cytoplasm, indicating that these fused proteins (or likely in the native context) are not incorporated within Eut BMCs. EutQ and EutP were assumed to function as acetate kinases (*27*). Our results show that only EutQ is encapsulated within the Eut BMC, possibly catalyzing the formation of acetate and adenosine triphosphate (ATP) from acetyl phosphate generated from EutD (see further analysis below). Our data lead us to propose a model for the protein organization of the Eut BMC (Fig. 1E).

In addition to the canonical 17-gene *eut* operon, recent studies have indicated 19 adjacent genes that show conservation across the *eut* loci in various *Salmonella* strains (*16*, *28*). To investigate whether these gene products are involved in Eut BMC biogenesis and function, we performed mass spectrometry–based proteomics on *S.* Typhimurium LT2 cells grown without or with EA induction at 15, 30, 60, and 300 min (fig. S4). Our results reveal that EA induction stimulated the production of all 17 Eut proteins to different levels at a variety of time points, with increases ranging from 11- to 63-fold after 300 min. We then analyzed the expression levels of 17 of the 19 ancillary proteins (fig. S4), with MaeB and TalA proteins showing the highest abundance. Following EA induction (300 min), AmiA and YpfG proteins exhibited two- and fivefold increases, respectively. These results suggest a correlation between these ancillary proteins and Eut BMC biogenesis and assembly. To test this, we determined the cellular locations of the four proteins relative to Eut BMC in vivo localization using fluorescence tagging. Our results showed that MaeB, TalA, and AmiA were distributed evenly in the cytoplasm, whereas YpfG was located in the cell membrane (fig. S5). We conclude that the four proteins were not physically incorporated into Eut BMCs. Their precise roles in Eut BMC biogenesis and function merit further investigation.

### Roles of individual Eut proteins in Eut BMC assembly

To decipher the roles of individual protein components in the Eut BMC assembly, we generated a series of mutants that lacked individual Eut proteins via scarless deletion of the corresponding *eut* genes (Δ*eutS*, Δ*eutM*, Δ*eutN*, Δ*eutL*, Δ*eutK*, Δ*eutQ*, and Δ*eutJ*) (fig. S6A). The dual-labeling plasmid pBAD-*eutM-mCherry*::*eutB-sfGFP* was then transformed into these deletion mutants to visualize the assembly of Eut shell proteins (indicated by EutM) and cargos (indicated by EutB).

**Fig. 2.**
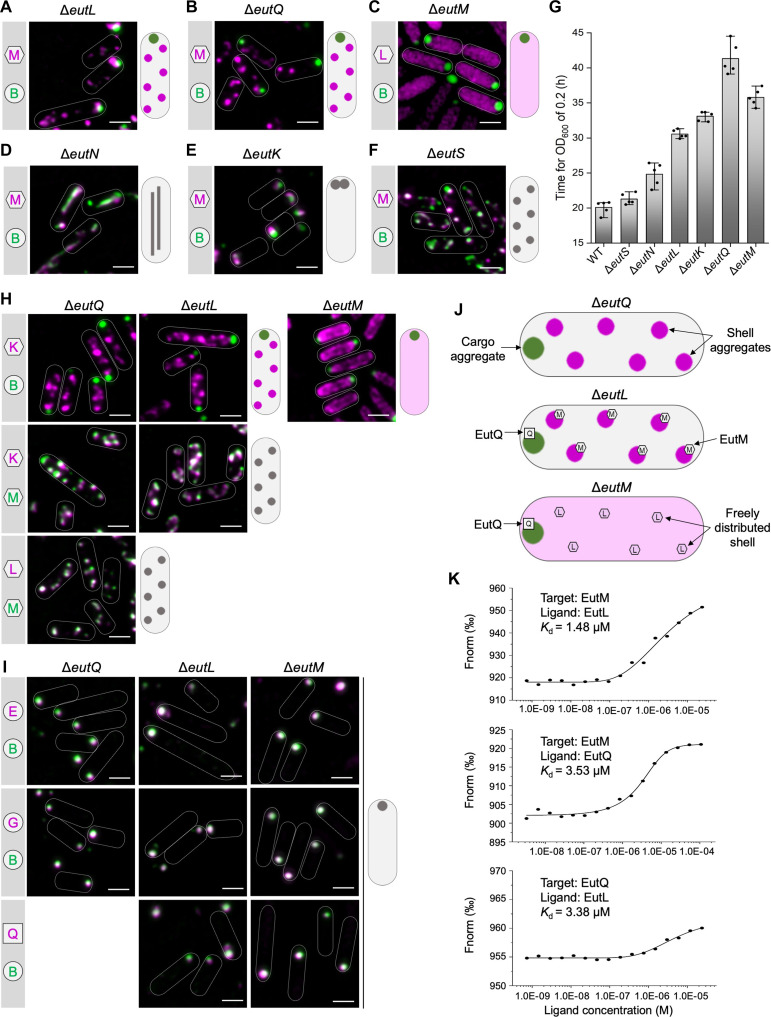
Roles of *eut* operon-encoding proteins in the assembly of Eut BMCs in *S.* Typhimurium LT2. (**A** to **F**) Localization of shell proteins and cargos in the gene deletion mutants in the absence of EutL, EutQ, EutM, EutN, EutK, or EutS, which were grown in the presence of EA and B_12_. EutM-mCherry (shell) and EutB-sfGFP (cargo) were imaged to indicate the locations of shell proteins and cargos. In the Δ*eutM* mutant, EutL-mCherry and EutB-sfGFP were imaged to indicate the locations of shell proteins and cargos. Schematic models of the in vivo localization of shell and cargo assemblies are shown on the right. (**G**) Time for LT2-WT and mutants to grow to reach OD_600_ = 0.2 in M9 medium supplemented with EA and B_12_ (*n* = 5). The center for error bars represents the mean. The whiskers extend to the smallest and largest data points that are within 1.5 times the interquartile range of the upper and lower quartiles. *n*, number of biologically independent experiments. h, hours. (**H** and **I**) Locations of Eut proteins in the Δ*eutQ*, Δ*eutL*, and Δ*eutM* strains. All strains were imaged following growth in the minimum medium in the presence of EA and B12. Scale bars, 1 μm. (**J**) Models for the locations of Eut proteins in Δ*eutQ*, Δ*eutL*, and Δ*eutM.* (**K**) MST analysis of EutM, EutL, and EutQ shows their interactions with each other.

In the absence of EutL or EutQ, EutB-sfGFP aggregated into a large punctum at a cell pole, whereas EutM-mCherry formed multiple clustered assemblies in the cytoplasm, suggesting spatial separation of shell and cargo proteins (Fig. 2, A and B). Thin-section EM of the *Salmonella* strains with and without fluorescent labels verified the observations from fluorescence imaging (fig. S7). We conclude that both EutL and EutQ play essential roles in mediating association between the shell and cargo proteins of Eut BMCs. The growth rates of the Δ*eutL* and Δ*eutQ* strains were reduced compared with the wild-type (WT) strain (Fig. 2G and fig. S8), possibly due to the release of toxic acetaldehyde, the impact of cytosolic redox state on cargo enzymatic functions, or improper enzyme stoichiometry within the formed polar aggregates. The Δ*eutQ* grew even slower than Δ*eutL*, consistent with EutQ acting as an acetate kinase during EA degradation (*27*). Expression of EutL or EutQ in the Δ*eutL* or Δ*eutQ* strain, respectively, partially rescued the assembly of Eut BMCs, evidenced by colocalization of EutM-mCherry and EutB-sfGFP and growth assays (fig. S9). We found that complementation of EutL resulted in various aggregates mostly at cell poles, whereas complementation of EutQ led to formation of rodlike structures (fig. S9A). These results suggest that precise levels of EutL/EutQ are required to define the assembly of native Eut BMCs.

For the Δ*eutM* strain, we used the pBAD-*eutL-mCherry*::*eutB-sfGFP* to monitor the assembly of shell proteins (represented by EutL) and cargos (represented by EutB) (Fig. 2C). In the absence of EutM, EutB-sfGFP formed a large punctum at a single polar position within the cell, whereas EutL-mCherry was evenly distributed throughout the cytosol (Fig. 2C and fig. S7A). The Δ*eutM* strain exhibited a growth defect on EA compared to the WT (Fig. 2G). These results indicate that EutM may initiate the assembly of the Eut BMC shell.

In the Δ*eutN* mutant, Eut assemblies were elongated (Fig. 2D and fig. S7A), suggesting that EutN proteins play an important role in shaping the Eut BMC structure. This is consistent with the functions of shell vertex pentameric proteins in other BMCs, including CsoS4 in the α-carboxysome (*29*), CcmL in the β-carboxysome (*30*), and PduN in the Pdu BMC (*23*, *31*). EutN is the only CsoS4/CcmL homolog in *S. enterica*, and no other pentamers have been identified in Eut BMCs (*32*). EutN was characterized to be pentameric in solution (*33*), whereas crystallization analysis showed that EutN was a homohexamer (*30*, *34*), suggesting the quaternary structural flexibility of the shell proteins under varying environments.

In Δ*eutK*, Eut BMCs were predominantly located at cell poles (Fig. 2E and fig. S7A) with the polar Eut BMC assemblies being more static than the highly dynamic Eut BMCs in the WT (movies S1 and S2). We conclude that EutK plays a role in determining the spatial distribution of Eut BMCs in *S.* Typhimurium. The Δ*eutK* strain exhibited a slower growth rate than the WT on EA (Fig. 2G and fig. S8). Complementation experiments showed that both Eut BMC formation and cell growth recovered when EutK was expressed in Δ*eutK* (fig. S9).

In the absence of EutS, EutM-mCherry and EutB-sfGFP colocalized into fluorescent foci (Fig. 2F), resembling the typical Eut BMC distribution in WT cells (Fig. 1C). Thin-section EM confirmed the formation of Eut BMC in Δ*eutS* (fig. S7A). The Δ*eutS* strain exhibited a similar growth profile as the WT using EA as the sole carbon source (Fig. 2G and fig. S8), consistent with previous observations (*35*). These results suggest that EutS is not essential for the assembly of Eut BMCs. Similarly, the deletion of EutJ, a putative chaperone-like protein (*36*) or an MreB/FtsZ homolog (*28*), did not remarkably affect Eut BMC assembly or the growth of *S.* Typhimurium (fig. S10).

To sum up, our systematic analyses revealed the essential roles of EutM, EutN, EutL, EutK, and EutQ for determining the structural integrity and growth of Eut BMCs.

### EutQ governs the encapsulation of the enzymatic core to the shell

Our results showed that, in the absence of EutL, EutQ, and EutM, the cargo enzymes were spatially separated from the shell proteins; cargos formed one aggregate at the cell pole, whereas shell proteins appeared as multiple aggregates (Δ*eutL* and Δ*eutQ*) or were evenly distributed (Δ*eutM*) in cells (Fig. 2, A to C). To investigate the protein composition of these self-assemblies, we determined the locations of Eut proteins in the Δ*eutQ*, Δ*eutL*, and Δ*eutM* strains grown upon EA and B_12_.

In Δ*eutQ* and Δ*eutL*, the shell protein EutK was dissociated from the cargo EutB but colocalized with the shell protein EutM to form multiple aggregates (Fig. 2H). In Δ*eutM*, EutK was freely distributed in the cytoplasm (Fig. 2H), which fits with the observations for EutL (Fig. 2C), confirming that EutM drives shell formation. Furthermore, in Δ*eutQ*, Δ*eutL*, and Δ*eutM*, the cargo proteins EutE and EutG colocalized with EutB, forming a single polar aggregate (Fig. 2I). These findings indicate that the lack of EutQ, EutL, or EutM could cause shell-cargo disassociation, resulting in the formation of a cargo aggregate at the cell pole coupled with multiple shell aggregates or the dispersion of shell proteins within cells (Fig. 2, A to C and H to J).

Our results suggest that interactions between EutQ and EutL or/and EutM may be essential for Eut BMC cargo-shell association. To test this hypothesis, we examined the locations of EutL and EutM in Δ*eutQ* and the distribution of EutQ in Δ*eutL* and Δ*eutM*. In the absence of EutQ (Δ*eutQ*), EutL and EutM assembled together to form shell aggregates (Fig. 2H) but disassociated with cargos (Fig. 2, B and H). In Δ*eutL* and Δ*eutM*, EutQ formed a punctum at the cell pole that colocalized with cargos (Fig. 2I). We conclude that EutQ forms a strong association with cargos while interacting with the shell proteins EutM and EutL, which is essential for cargo encapsulation. The findings from Δ*eutL* show that shell assemblies disassociated with cargos in the presence of EutM (Fig. 2, H and J), suggesting that the interactions between EutQ and EutM were insufficient for cargo encapsulation. A previous study has indicated that EutQ was required for Eut BMCs assembly and its interaction with EutM through pull-down assays (*37*). Our microscale thermophoresis (MST) analysis further revealed mutual interactions between EutQ, EutM, and EutL (Fig. 2K and fig. S11). We propose that the binding of EutQ to the shell is facilitated by the shell proteins EutL and EutM and that EutQ specifically attaches at the interaction interface between EutM and EutL.

### Molecular mechanisms of the EutQ-mediated shell-cargo association

**Fig. 3.**
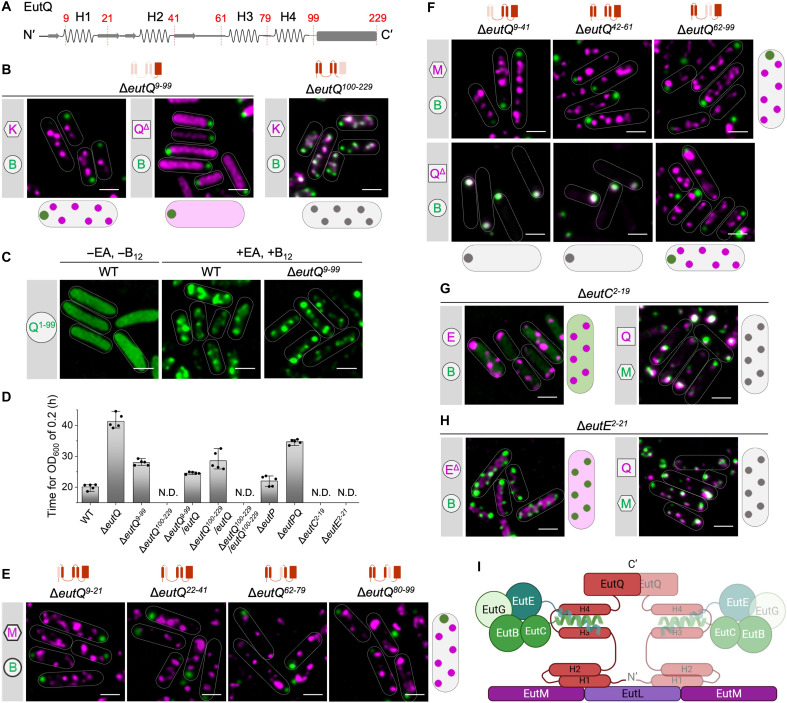
EutQ plays an essential role in mediating the binding of the shell and cargos. (**A**) Secondary structure of EutQ with the N terminus predicted by AlphaFold3. H1 to H4 represent four α helix structures. The amino acid sites for genome editing were labeled and colored in red. (**B**) EutK-mCherry/EutB-sfGFP and EutQ^Δ^-mCherry/EutB-sfGFP were visualized in Δ*eutQ*^*9*–*99*^; EutK-mCherry/EutB-sfGFP were visualized in Δ*eutQ*^*100*–*229*^ in the presence of EA and B_12_. (**C**) Localization of EutQ^1–99^-sfGFP in LT2-WT and Δ*eutQ*^*9*–*99*^. (**D**) Time for LT2-WT and mutants to grow to OD_600_ = 0.2 on EA and B_12_ in M9 medium (*n* = 5). The center for error bars represents the mean. The whiskers extend to the smallest and largest data points that are within 1.5 times the interquartile range of the upper and lower quartiles. *n*, number of biologically independent experiments. “N.D.” stands for “not detected,” due to the lack of growth in the tested bacterial strains. (**E** and **F**) EutM-mCherry/EutB-sfGFP were visualized in Δ*eutQ*^*9*–*21*^ Δ*eutQ*^*22*–*41*^, Δ*eutQ*^*62*–*79*^, Δ*eutQ*^*80*–*99*^, Δ*eutQ*^*9*–*41*^ Δ*eutQ*^*42*–*61*^, and Δ*eutQ*^*62*–*99*^; EutQ^Δ^-mCherry/EutB-sfGFP were visualized in Δ*eutQ*^*9*–*41*^ Δ*eutQ*^*42*–*61*^, and Δ*eutQ*^*62*–*99*^ in the presence of EA and B_12_. EutQ^Δ^ represents the truncated EutQ with different fragments deleted, which differs from each other in different genome editing mutants. The deletion parts of EutQ were shown in the models on the top of (B), (E), and (F). (**G** and **H**) EutE-mCherry/EutB-sfGFP and EutQ-mCherry/EutM-sfGFP were visualized in Δ*eutC*^*2*–*19*^ and Δ*eutE*^*2*–*21*^ in the presence of EA and B_12_. EutE^Δ^ represents the truncated EutE with EutE^2–21^ deleted. (**I**) Schematic model for the assembly mode between EutQ and shell/cargo. EutQ is shown as a dimer, and the dimerization site is at the C terminus. Scale bars, 1 μm.

Size exclusion chromatography coupled with multiangle light scattering analysis revealed that EutQ (~25 kDa per monomer) forms an ~50-kDa dimer and that dimerization was mediated by the contacts between two EutQ C termini (fig. S12). This is consistent with the crystal structure of EutQ C terminus (Protein Data Bank ID: 2PYT) (*38*). AlphaFold prediction of the entire EutQ structure shows that the EutQ N-terminal region (EutQ^1–99^) has four α helices (H1 to H4) and a long flexible unstructured region linking H2 and H3. The C-terminal region (EutQ^100–229^) consists of mainly β strands (Fig. 3A and fig. S13, A and B), representing a cupin barrel fold domain (Pfam: PF06249) that is widespread and conserved among bacteria.

To determine the function of EutQ in Eut BMC assembly, we generated two mutants that lacked the EutQ N terminus (Δ*eutQ*^*9*–*99*^; note that *eutQ*^*1*–*9*^ overlaps with the upstream gene *eutP*) or the C terminus (Δ*eutQ*^*100*–*229*^). Deletion of EutQ^9–99^ caused disassociation of the shell and cargos and formation of a polar cargo aggregate along with numerous shell assemblies (Fig. 3B and fig. S13, C and E), in line with the observations from the deletion of full-length EutQ (Fig. 2 and fig. S7). In the absence of EutQ^9–99^ (Δ*eutQ*^*9*–*99*^), the EutQ C terminus exhibited an even distribution throughout the cytosol and did not assemble with either the shells or the cargos (Fig. 3B). The EutQ N terminus (EutQ^1–99^) was sufficient to target sfGFP to Eut BMC, both in the presence and absence of EutQ^9–99^ (Fig. 3C). By contrast, Eut BMCs were formed in Δ*eutQ*^*100*–*229*^ (Fig. 3B and fig. S13, D and F). These results demonstrate that the EutQ N terminus plays a critical role in mediating the shell-cargo association, whereas the EutQ C terminus is not essential for Eut BMC assembly.

Both the Δ*eutQ*^*9*–99^ and Δ*eutQ* mutants had reduced growth rates compared to the WT (Fig. 3D and fig. S14). We propose that the absence of EutQ coordinating function upon loss of the full protein (Δ*eutQ*) or the N-terminal helices involved in interactions with cargo and shell proteins (Δ*eutQ*^*9*–99^) adversely affects bacterial growth under EA utilization. Both EutQ and EutP have been suggested to have acetate kinase activity, facilitating the conversion of acetate and ATP into acetyl phosphate and vice versa; however, EutQ is required for *Salmonella* growth, but EutP is not (*27*). Likewise, EutQ was found to be 10 times more abundant than EutP in *Escherichia coli* (*39*). A recent study assumed that both EutP and EutQ are cytosolic, highlighting that their subcellular localization requires further investigation (*39*). Our results demonstrate that EutQ is incorporated within the Eut BMC, whereas EutP is most likely cytosolic (Fig. 1D). We found that Δ*eutP* had a similar growth rate to the WT and, unexpectedly, that Δ*eutPQ* showed slightly faster growth than Δ*eutQ* (fig. S14). This presumably reflects the presence of the housekeeping acetate kinase (AckA) in the cytosol, which is encoded by the *ackA* gene outside the *eut* operon (*40*, *41*). In Δ*eutQ*^*9*–*99*^, formation of acetate can be catalyzed by EutQ^100–229^ that contains the C-terminal catalytic domain of EutQ, ensuring a faster growth of Δ*eutQ*^*9*–99^ than Δ*eutQ* (Fig. 3D and fig. S14). Moreover, Δ*eutQ*^*100*–*229*^ cannot grow on EA as the sole carbon source. In Δ*eutQ*^*100*–*229*^, Eut BMCs were still formed, but the access of acetyl phosphate to the external acetate kinase was limited by shell encapsulation, resulting in a slower growth rate of Δ*eutQ*^*100*–*229*^ compared to other strains (Fig. 3D and fig. S14). The growth of Δ*eutQ*^*100*–*229*^ was restored by expression of full-length EutQ (Fig. 3D and fig. S14). However, expression EutQ^100–229^ in Δ*eutQ*^*100*–*229*^ did not restore growth, suggesting that EutQ^100–229^ alone could not be encapsulated into the Eut BMC without the EutQ N terminus.

The encapsulation of EutQ suggests its role in the localized acetyl phosphate metabolism, enabling ATP generation within Eut BMCs. Because the large adenosine diphosphate (ADP) and ATP molecules may be difficult to pass through the shell pores, this localized ATP pool may serve as a local energy source for EutBC reactivation by EutAT (*42*). In this context, the presence of an acetate kinase within the BMC core may be essential for sustaining enzymatic activity and maintaining efficient metabolic flux within the BMC lumen.

To determine how the N terminus of EutQ mediates shell-cargo association, we deleted the H1 to H4 α helices individually (Δ*eutQ*^*9*–*21*^, Δ*eutQ*^*22*–*41*^, Δ*eutQ*^*62*–*79*^, and Δ*eutQ*^*80*–*99*^) and examined the subcellular locations of shell and cargo proteins in these mutants. Deletion of each α helix led to the disassociation of the shell and cargos (Fig. 3E), indicating that all four α helices are essential for Eut BMC formation. The AlphaFold-predicted EutQ structure and EutQ^1–99^-EutC^1–20^ interactions (figs. S13A and S15) led us to propose that H1/H2 interacts with the shell and that H3/H4 is responsible for anchoring the EPs of cargos. To test this hypothesis, we deleted the H1/H2 (Δ*eutQ*^*9*–*41*^), H3/H4 (Δ*eutQ*^*62*–*99*^), and the flexible region (Δ*eutQ*^*42*–*61*^) individually. The shell and cargo assemblies were spatially separated in all three mutants (Fig. 3F and fig. S16), suggesting that the three regions were crucial for shell-cargo association. Furthermore, EutQ^Δ9–41^ and EutQ^Δ42–61^ aggregated with the cargo cores at the cell poles in Δ*eutQ*^*9*–*41*^and Δ*eutQ*^*42*–*61*^, whereas EutQ^Δ62–99^ dissociated with the cargos and colocalized with the shell assemblies in Δ*eutQ*^*62*–*99*^ (Fig. 3F and fig. S17).

Together, our results demonstrate that the EutQ N-terminal H1/H2 interacts with the shell whereas H3/H4 associates with cargos. We propose that the interactions between H3/H4 and cargos were stronger than those between H1/H2 and the shell because the EutQ that lacked the flexible region (EutQ^Δ42–61^) colocalized with cargos rather than shells (Fig. 3F, middle bottom). This finding indicates that the flexible region plays an important role in shell-cargo binding and potential interactions with the shell.

We then examined the roles of endogenous cargo-derived EPs by studying their interactions with EutQ. Deletion of the N terminus of EutC (Δ*eutC*^*2*–*19*^) caused cytosolic distribution of EutB, whereas EutQ, the cargo protein EutE, and the shell protein EutM could still form assemblies individually (Fig. 3G). These results suggest that the EutC N terminus was specifically responsible for the assembly and encapsulation of EutBC cargo complexes. Likewise, removal of the native EP of EutE (Δ*eutE*^*2*–*21*^) resulted in an even distribution of EutE^Δ2–21^ throughout the cytosol, whereas the subcellular locations of other Eut proteins were not affected (Fig. 3H), identifying a role for the native EP in targeting EutE to the Eut BMC. Growth assays of the EP deletion mutants showed no growth on EA as the sole carbon source after 50 hours (Fig. 3D and fig. S14), indicating that, without the EPs, EutBC and EutE could not be incorporated into Eut BMCs. The location of another cargo protein, EutG, was not affected in both Δ*eutC*^*2*–*19*^ and Δ*eutE*^*2*–*21*^ (fig. S18). EutG has an N-terminal extension with an unknown function (fig. S19A). Our results reveal that, although this extension played a role in mediating the incorporation of EutG into Eut BMCs (fig. S19B), it was insufficient to independently target sfGFP to Eut BMCs (fig. S19C). The N terminus of EutG lacks canonical EP features (fig. S19D), and EutG proteins are likely incorporated into Eut BMCs by “piggybacking” with other cargo enzymes (*25*, *42*). Collectively, our results elucidate the mechanisms by which the EutQ N terminus mediates the assembly of the Eut shell and cargos (Fig. 3I).

**Fig. 4.**
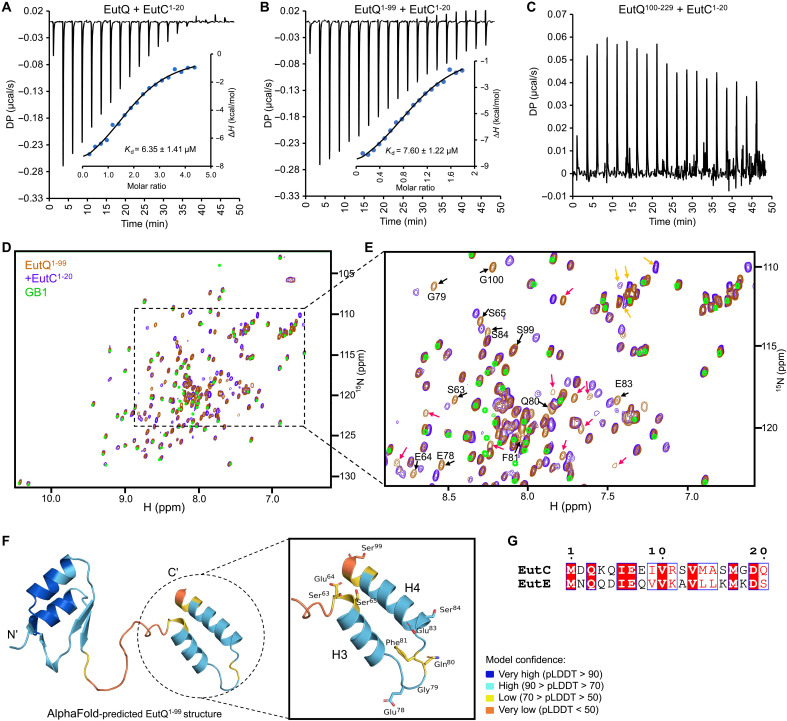
Interactions between EutC^1–20^ and EutQ^1–99^. Fitted isotherm of EutC^1–20^ binding with (**A**) EutQ (*N* = ~2, dimeric EutQ with ligand-binding domain contributed by each subunit; ∆*G*° = −7.10 kcal mol^−1^; ∆*H*° = −9.24 ± 0.01 kcal mol^−1^; *T*∆*S* = −2.14 kcal mol^−1^), (**B**) EutQ^1–99^-GB1 (*N* = ~1; ∆*G*° = −6.98 kcal mol^−1^; ∆*H*° = −9.63 ± 0.48 kcal mol^−1^; *T*∆*S* = −2.65 kcal mol^−1^), and (**C**) EutQ^100–229^ (no binding) by ITC. Inset: Thermodynamic parameters. Entropic parameters of binding suggest hydrophilic/polar-driven interaction between EutQ and EutC^1–20^. (**D**) 2D ^15^N-^1^H HSQC spectrum (298 K) of ^15^N uniformly labeled EutQ^1–99^-GB1 overlaid with (i) uniformly ^15^N-labeled EutQ^1–99^-GB1 (red) mixed with unlabeled EutC^1–20^ (purple) and (ii) uniformly^15^N-labeled GB1 (green). The CSPs observed in EutQ^1–99^-GB1 in the presence of EutC^1–20^ without equivalent shifts on GB1 indicates the specificity of peptide interaction occurring within EutQ^1–99^ alone. ppm, parts per million. (**E**) Zoomed-in region of CSPs arising from EutQ^1–99^-GB1 peptide binding. Chemical shift changes occurring in assigned peaks are highlighted with black arrows, whereas those occurring in unassigned peaks are shown in red arrows. Peaks of Gln and Asn side chains that undergo CSPs are designated with yellow arrows. CSPs of amino acid residues 63 to 65, 78 to 81, 83, 84, and 99 of EutQ^1–99^ in the presence of the peptide were observed, whereas peaks assigned to residues 2 to 62 remained unchanged. Severe line broadening of many residues in H3 and H4 helices precluded the specific assignments of many resonances from H3 and H4, limiting the completeness of the data. (**F**) AlphaFold-predicted structure of EutQ^1–99^. Resonances that were affected by the presence of EutC^1–20^ are confined to residues from the linker and C-terminal region of EutQ^1–99^. This indicates that the interacting region of the EutQ^1–99^ with EutC^1–20^ spans the 63 to 99 amino acid region. (**G**) Sequence alignment of EutC^1–20^ and EutE^1–20^.

To determine how EutQ binds cargo EPs, we examined the protein-protein interactions using isothermal titration calorimetry (ITC) and NMR spectroscopy. The ITC results revealed strong binding between EutC^1–20^ and EutQ [dissociation constant (*K*_d_) = 6.35 μM] or between EutC^1–20^ and the N-terminal region of EutQ, EutQ^1–99^, fused with a soluble fusion partner, the immunoglobulin binding domain of streptococcal protein G (GB1) (*43*) (*K*_d_ = 7.60 μM) (Fig. 4, A and B, and fig. S20). A binding stoichiometry of 2 for the EutQ interactions suggests that EutQ functions as a dimer. This is in contrast to EutQ^1–99^-GB1, where the binding stoichiometry to the same EutC peptide is 1, consistent with the monomeric state of EutQ^1–99^. No binding was detected between EutC^1–20^ and EutQ^100–229^ (Fig. 4C) or for GB1 alone or for the incubation buffer (fig. S21). Two-dimensional (2D) heteronuclear single-quantum correlation (HSQC) NMR identified interactions between EutQ^1–99^ and EutC^1–20^, indicated by chemical shift perturbations (CSPs) of selected residues of ^15^N-labeled EutQ^1–99^ in the presence of unlabeled EutC^1–20^ (Fig. 4D). Complete backbone assignment of EutQ^1–99^-GB1 was hampered by severe resonance overlap and/or line broadening due to the propensity of the H3/H4 region to aggregate; specifically, resonances from the residues 66 to 77, 82, and 85 to 98 within H3 and H4 regions could not be assigned (Fig. 4E and fig. S22). The absence of CSPs of residues in H1 and H2 (which were all assigned) confirmed that this region of EutQ is not involved in interactions with EutC^1–20^. Assigned resonances showing CSPs include amino acids 63 to 65, 78 to 81, 83, 84, and 99; these are residues found in the linker region and within H3/H4 of EutQ (Fig. 4F). These NMR results confirmed the in vivo findings (Fig. 3) and the AlphaFold predictions described above (fig. S15). Furthermore, molecular dynamics (MD) simulations revealed that the amino acids I6, V10, M14, and V13 of EutC^1–20^ lie on one side of the peptide helices and interact with the conserved amino acids V93, L89, E64, R67, and Q75 of EutQ (figs. S23 and S24). Point mutation of EutQ (figs. S25 and S26) showed that the mutants V93A and L89A, with the alternations to hydrophobic residues, completely eliminated the binding of EutQ to EutC^1–20^. The Q75A mutant exhibited a higher *K*_d_ (*K*_d_ = 21.5 μM) compared to the WT EutQ, indicating reduced binding affinity. These results suggest that the EutQ-EutC^1–20^ interaction involves both hydrophobic and hydrophilic amino acids, with hydrophobic interactions playing a dominant role in complex stabilization.

We investigated the binding between EutQ and EutE^1–20^, the EP of the distinct EutE cargo protein, using ITC and NMR (fig. S27). The EutE binding characteristics, including the binding site and thermodynamics, resembled those observed for EutC^1–20^ binding, albeit with a notable decrease in affinity. EutC^1–20^ and EutE^1–20^ helical peptides show 90% homology with 40% identity (Fig. 4G). The difference between the affinities of EutC and EutE to EutQ could be explained by slight variations in the hydrophilic interactions between the two peptides and EutQ. Together, our results suggest that the EutC^1–20^ and EutE^1–20^ peptides interact with EutQ to mediate cargo encapsulation.

Comparative genomic analysis and protein sequence alignment showed that Eut proteins are highly conserved in sequence among Gram-negative bacteria (figs. S28 and S29). EutQ homologs are also known as components of another type of metabolosomes, the glycyl radical enzyme-associated microcompartments (GRMs) (*18*), implying that the EutQ-mediated assembly mechanisms of Eut BMCs we have found in *S.* Typhimurium may serve as a general principle for the assembly of BMCs in a variety of bacterial species. In contrast, the Eut proteins from Gram-positive bacteria exhibit low sequence similarity compared to *S.* Typhimurium counterparts (fig. S28). Notably, the EutQ proteins of Gram-positive species like *Enterococcus faecalis* and *Listeria monocytogenes* lack the first three α helices (fig. S29), which, according to our results, are crucial for BMC assembly in *S.* Typhimurium. This difference strongly indicates that the assembly mechanisms of Eut BMCs may vary substantially between Gram-negative and Gram-positive bacteria. Instead of one universal and conserved assembly pathway, our results highlight that Eut BMC assembly follows various mechanisms across different bacterial species.

### Eut BMC undergoes a shell-initiated assembly pathway

**Fig. 5.**
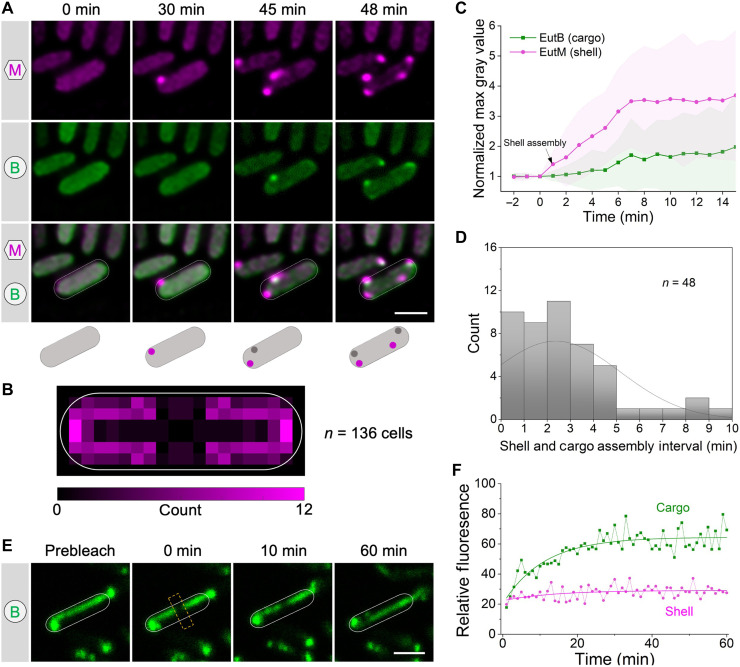
Eut BMC undergoes a “shell-initiated” assembly pathway and cargo enzymes form liquid-like condensates within the Eut BMC. (**A**) Aggregation of the shell (EutM-mCherry) and cargo (EutB-sfGFP) in the WT strain following induction with EA and B_12_. Scale bar, 1 μm. (**B**) Heatmap of the spatial distribution of the first Eut BMC shell within the cell (*n* = 136). The scale bar represents the count number of initial shell fluorescence observed at the corresponding location of the cell. (**C**) Time course of the normalized max fluorescence intensity of the shell and cargo of the Eut BMC. The time point of the first shell assembly was normalized to 1 min. The center for error bars represents the median. (**D**) Histogram of the time interval between the first shell and cargo assembly of the Eut BMC. (**E**) Representative FRAP images on EutB-sfGFP in Δ*eutN* at various time lapses. The yellow rectangular boxes indicated the bleaching area. Scale bar, 1 μm. (**F**) Representative time courses of fluorescence recovery of bleached regions of EutB-sfGFP (green) and EutM-sfGFP (magenta). The *y* axis indicates fluorescence values relative to the fluorescence intensity of the selected region before bleaching.

To delineate the de novo biogenesis of Eut BMCs, we conducted time-lapse live-cell fluorescence imaging to monitor the initial in vivo assembly of cargo and shell proteins. We expressed EutM-mCherry/EutB-sfGFP, EutK-mCherry/EutB-sfGFP, or EutB-mCherry/EutM-sfGFP in *S.* Typhimurium cells. After adding EA and B_12_ metabolites to induce expression of native Eut proteins, the first fluorescent spot of Eut shell proteins (indicated by EutM-mCherry, EutK-mCherry, or EutM-sfGFP) emerged from the cytosolic distribution fluorescence background after ~30 min of induction. For comparison, *Salmonella* cells growing on solid media under the microscope exhibited localization patterns similar to those seen in liquid media (Fig. 5A and fig. S30), indicating that the observed localization patterns accurately reflect the authentic in vivo organization of Eut BMCs. Intriguingly, the assembly of shell proteins always appeared before the assembly of cargo proteins (Fig. 5A and fig. S31, A and B). We observed that the first shell assembly tended to occur at the cell pole and along the cell membrane (Fig. 5B). The time interval between the first shell and cargo assembly was 2.4 ± 2.2 min (means ± SD, *n* = 48) (Fig. 5D); in 62.5% of cells, this interval was less than 3 min.

Following initiation of assembly, the fluorescence intensity of shell and cargo assemblies continued to increase and reached a steady state at ~6 to 7 min (Fig. 5C and fig. S32). Consistent with this, during the early stage (60 min) after EA induction, our proteomic analysis revealed a greater increase in the level of shell proteins (for example, EutM) compared to cargo proteins (such as EutBC) (fig. S4). Together, the results strongly support our proposed “shell-initiated” pathway, where Eut BMC biogenesis is initiated with the assembly of shell proteins followed by the nascent, partially formed shell assemblies serving as scaffolds for recruiting cargo enzymes and incorporating other shell proteins, ultimately leading to the formation of entire Eut BMCs. This pathway is distinct from a combination of the “shell first” and “cargo first” pathways of Pdu BMCs (*23*) (fig. S31, C and D).

### Liquid-like association of cargo enzymes within the Eut BMC

The Eut BMC sequesters a series of cargo protein complexes within the shell. These cargo enzymes can oligomerize via interactions mediated by native EPs, whereas enzymes that lack EPs “piggyback” onto the enzymes containing EPs to achieve encapsulation. To understand the organizational properties of Eut BMCs, we determined the diffusion dynamics of the shell (indicated by EutM) and cargos (indicated by EutB) of Eut BMCs by fluorescence recovery after photobleaching (FRAP). Elongated structures of Eut BMCs formed in the Δ*eutN* strain were used for FRAP experiments and analysis (Fig. 5E and fig. S33). We found that cargo proteins showed a higher mobile fraction (58.5 ± 4.5%, *n* = 20) than shell proteins (12.8 ± 3.0%, *n* = 20) (Fig. 5F); the half-life of recovery of the cargos was 3.85 ± 2.0 min (*n* = 20). We conclude that the cargo proteins form a liquid-like condensate within the Eut BMC, consistent with the findings for α- and β-carboxysomes and for Pdu BMCs of *S.* Typhimurium (*23*, *44*, *45*). These findings suggest that oligomerization and condensation of cargo proteins are a widespread phenomenon that occurs within a diverse range of BMCs, which plays a crucial role in BMC assembly and in sustaining their structures and functions.

## DISCUSSION

**Fig. 6.**
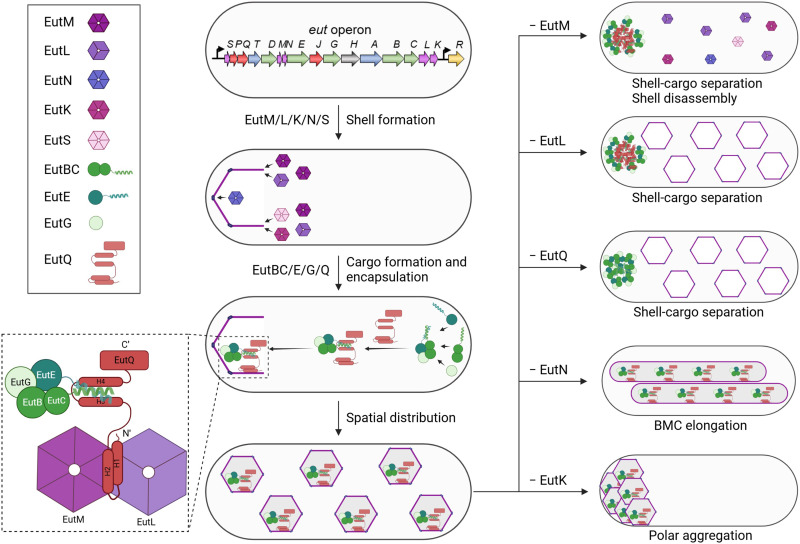
Schematic model of Eut BMC biogenesis. The Eut BMC shell proteins are first recruited by EutM to assemble at a cell pole. Then, cargo proteins self-aggregate at a cell pole and are encapsulated into shell through interactions between EutQ and the N-terminal EPs of cargos. EutQ forms a bridge between the cargo and shell proteins: Helices H1 and H2 bind the interface between shell proteins, EutL and EutM, whereas cargos are bound to the H3 and H4 helical region, which provides the binding sites for the recruitment of the enzymatic core with shell proteins. The EutN proteins, which likely occupy the vertices, are important for full encapsulation of individual Eut BMC. EutK is responsible for the subcellular partitioning of Eut BMCs after their biogenesis at the cell poles. EutK is a hexamer due to a single BMC domain at its N terminus. EutS is a shell component but appears to be not essential for formation of the Eut BMC shell.

Eut BMCs are self-assembling proteinaceous organelles that mediate EA catabolism. Previous attempts using protein crystallization, growth analysis, and synthetic biology had only provided fragmented knowledge about the composition and function of Eut BMCs. The mechanism by which various protein components connect and self-assemble to create a complete and functioning entity remains unclear. Here, we have elucidated the organizational and assembly mechanisms of Eut BMCs in *S.* Typhimurium in unprecedented detail and propose a model for the biogenesis pathway of Eut BMCs (Fig. 6).

The assembly of the Eut BMC shell, triggered by EutM, initially occurs at the cell pole. Next, EutQ interacts with the interface of EutM and EutL to provide the binding sites for endogenous EPs of cargo enzymes, enabling cargo encapsulation. The binding mode between EutQ and EutM/L aligns with a model proposed by Aussignargues *et al.*, in which EPs interact with an epitope created by the interactions of multiple shell proteins (*42*). It should be noted that tagging fluorescent proteins may potentially cause artifacts of BMC assembly. To minimize possible interference, low levels of fluorescently tagged Eut proteins were expressed from a pBAD plasmid without induction and were incorporated, together with nontagged Eut proteins, into Eut BMCs. To corroborate our fluorescence imaging observations, we conducted a comprehensive set of control experiments and comparative analyses and used complementary techniques, including thin-section EM, mass spectrometry, and growth assays.

Our data reveal that the Eut BMC undertakes the shell-initiated assembly pathway, distinct from the assembly pathways of Pdu BMCs in *Salmonella* (concomitant assembly of shell and cargo proteins; fig. S31) (*23*), and β-carboxysomes in cyanobacteria using conventional fluorescence imaging and EM (“cargo first” pathway) (*46*, *47*). The biogenesis of Eut BMC structures begins with the initial assembly of shell proteins. These newly forming, incomplete shell structures then serve as structural frameworks, facilitating the attraction of cargo enzymes and the integration of additional shell components during subsequent maturation stages. This stepwise process ultimately leads to the formation of fully intact and functional Eut BMCs. In *Salmonella*, the distinct assembly pathways of Eut BMCs and Pdu BMCs might reflect differences in the availability of the particular shell and cargo proteins. Our proteomic analysis reveals a greater fold increase in Eut shell proteins compared to cargo proteins following EA induction for 60 min (fig. S4). In addition, previous transcriptomic analysis revealed higher expression levels of genes encoding Eut shell proteins than those encoding Eut cargos, whereas genes encoding *Salmonella* Pdu shell and cargo proteins were expressed at similar levels under anaerobic growth conditions (*48*). Moreover, the assembly of Eut BMC cargos occurs separately from the assembly of its shell as shell and cargo aggregates were spatially partitioned in the Δ*eutQ* and Δ*eutL* mutants (Fig. 2J). The independent assembly of shell and cargo proteins resembles the observations in Pdu BMCs (*23*). Together, our findings indicate that the shell and cargo assembly pathways of different metabolosomes are completely independent, and the temporal sequence of assembly of shell and cargo proteins varies considerably.

We found that the shell proteins, EutM, EutN, EutL, and EutK, were essential for the formation and function of Eut BMCs, but EutS was not required. We can draw parallels with the second *Salmonella*-encoded metabolosome, the Pdu BMC. PduU, the EutS homolog within the Pdu BMC, is not required for the assembly and function of Pdu BMCs (*38*, *49*, *50*). EutS appears to be well conserved among the isolates presented in fig. S33, suggesting that it may be under selective pressure and could play a role under more physiologically relevant conditions. EutM can self-assemble to form large protein filaments (*51*), which resemble the nanotube structures formed by the homologous PduA and PduJ proteins (*52*–*54*). On the basis of the fact that PduA and PduJ are the two major canonical shell proteins of Pdu BMCs (*49*), we propose that EutM is a major building component of the Eut shell. Our results also show that the pseudohexameric homotrimer EutL (*55*) is essential for the shell-cargo association of Eut BMCs (Fig. 2J). This is consistent with the function of its homolog, PduB, in the assembly of Pdu BMCs (*23*). EutK plays a role in the in vivo distribution of Eut BMCs, reminiscent of the role of PduK (with a disordered C-terminal extension) in determining the subcellular locations of Pdu BMCs (*49*). The C-terminal tail of EutK has a positively charged domain, which could potentially bind to nucleotides (*56*). This implies that EutK may function in a manner analogous to the ParB protein or associate with ParB, which plays a crucial role in chromosome segregation (*57*), to mediate Eut BMC positioning in *Salmonella*. Similarly, the McdAB system mediates the partitioning of carboxysomes in cyanobacteria and some proteobacteria (*58*, *59*). The molecular mechanism by which EutK governs the partitioning of Eut BMCs and interactions with nucleotides remains to be determined. Overall, our data indicate that Eut shell proteins perform similar functions to their counterparts in Pdu BMCs during the assembly of metabolosomes, suggesting that the specific functions of shell proteins we have identified may extend to a range of catabolic BMCs found in various bacterial hosts.

Our results demonstrate both the structural and catalytic roles of EutQ. The N terminus of EutQ links the shell and cargos of Eut BMCs, comparable to CcmN, CsoS2, and PduB^1–37^, the linkers proteins that mediate the binding of the shell and cargo enzymes of β-carboxysomes, α-carboxysomes, and Pdu BMCs, respectively (*23*, *45*, *46*). Recent studies have revealed that the interactions between CsoS2 C terminus and shell proteins occurred at the interface of shell proteins (*29*, *60*), in good agreement with our findings that EutQ could interact with the shell proteins EutL and EutM (Fig. 3I), whereas the N terminus of CsoS2 binds the cargo enzyme Rubisco in α-carboxysomes (*45*, *61*, *62*). We have shown that the H1/H2 region of EutQ N terminus specifically binds to the shell, whereas its H3/H4 region associates with cargo. Moreover, the C terminus of EutQ demonstrates acetate kinase activity and contains a cupin barrel fold domain. It is worth noting that many cupin proteins, including (*S*)-ureidoglycine aminohydrolase (*63*), are not associated with BMCs. We have found that the N terminus of EutQ is critical for the integration of the EutQ protein into Eut BMCs. Overall, EutQ serves as a multifunctional protein that integrates both structural and enzymatic roles within the BMC system.

In summary, our extensive live-cell super-resolution fluorescence imaging, structural, and biochemical studies provide unprecedented insights into the organization and biogenesis of Eut BMCs. We dissected the molecular mechanisms of the stepwise protein assembly and the hierarchical cooperation that governs the formation of structurally defined and functional Eut BMCs. This breakthrough advances our knowledge of catabolic BMC assembly and provides a molecular roadmap for reprogramming and modulating BMCs to improve the efficiency of metabolic reactions. The future identification of specific binding sites for BMC shell and cargo protein interactions could pave the way for the development of therapeutic interventions for a variety of infectious diseases that target the mammalian GI tract.

## MATERIALS AND METHODS

### Bacterial strains and growth conditions

The bacterial strains used in this study derived from *S. enterica* subsp. *enterica* serovar Typhimurium LT2 (*S.* Typhimurium LT2) (*64*). The rich medium was LB broth (Lennox) medium [tryptone (10 g liter^−1^), yeast extract (5 g liter^−1^), and sodium chloride (5 g liter^−1^)], and the minimal medium was no-carbon-E (NCE) medium supplemented with 1 mM MgSO_4_, 0.5% succinate, and 30 mM EA and 200 nM vitamin B_12_ (if applicable). The LB plate was prepared with agar (15 g liter^−1^), and the minimal medium plate was prepared with 2% (w/v) low–melting point agarose. Antibiotics were added to media as required at the following final concentrations: ampicillin at 100 μg ml^−1^, kanamycin at 50 μg ml^−1^, and gentamicin at 20 μg ml^−1^ in ddH_2_O, chloramphenicol at 25 μg ml^−1^ in ethanol, and tetracycline at 25 μg ml^−1^ in methanol.

Intracellular visualization of fluorescently tagged Eut proteins was conducted following the growth of the plasmid-carrying strains in minimal medium under aerobic conditions. An overnight LB culture was inoculated 1:100 in 100 μl of minimal medium in a 2-ml Eppendorf tube in the absence of EA and B_12_ shaken horizontally at 220 rpm overnight. Unless otherwise specified, 1 μl of this culture was subinoculated to 100 μl of minimal medium in a 2-ml Eppendorf tube, both in the absence and in the presence of EA and B_12_, shaken aerobically at 37°C for 5 hours. For birth event detection, 5 μl of the subinoculated culture (in minimal medium without EA and B_12_) was dropped onto the minimal medium plate in the presence of EA and B_12_ and incubated aerobically at 37°C for time-lapse imaging.

### Construction of chromosomal mutations

All mutants in this study were generated by a scarless genome editing technique developed previously using the pEMG and pSW-2 plasmids (*65*). A pair of DNA fragments (~800 base pairs) flanking the chromosome regions of interest (ROIs) were firstly polymerase chain reaction (PCR) amplified and integrated into the linear pEMG suicide plasmid (digested by EcoRI and BamHI) by Gibson assembly (NEBuilder HiFi DNA Assembly kit) (*66*). The pEMG-derivative plasmids were mobilized from *E. coli* S17-1 λ *pir* to *S.* Typhimurium by conjugation. *S.* Typhimurium transconjugants that have integrated pEMG-derivative plasmids were selected on M9 minimal medium plates supplemented with kanamycin and 0.2% glucose. pSW-2, extracted from *S.* Typhimurium LT2, was subsequently transformed into the transconjugants by electroporation. Then, transformants were selected on LB plates with gentamicin and 1 mM *m*-toluate was added. Colonies were screened for kanamycin resistance and sensitive clones were verified by PCR. pSW-2 was lastly cured from the resulting strains by two passages in LB without adding gentamicin. Please note that, for the deletion of N terminus of EutQ, the first nine amino acids were kept due to an overlap of coding genes between *eutP* and *eutQ*. Strains and plasmids used in this study are listed in table S1. A complete list of primers can be found in table S2. All mutants were verified by PCR and DNA sequencing of PCR-amplified genomic DNA sequencing (fig. S6).

### Construction of vectors

pBAD/*Myc*-His was used as a backbone for generation of fluorescence tagging vectors. pBAD/*Myc*-His was first digested by NcoI and HindIII. Then, PCR-amplified DNA fragments of individual *eut* genes, mCherry, and sfGFP were integrated into the linear vector by Gibson assembly (*66*). pXG10-SF containing a constitutive P_LtetO-1_ promoter was used as a backbone for complementation experiments (*67*). The PCR-amplified coding sequences of EutK/L/Q were inserted into linear pXG10-SF (obtained by PCR cloning) via Gibson assembly. Plasmids and primers used in this study were listed in tables S1 and S2, respectively. All constructed vectors were verified by PCR and plasmid sequencing.

### Bacterial growth assays

The *S.* Typhimurium strains of interest were firstly inoculated from isolated colonies from LB plates into 5 ml of LB in 30-ml universal glass vials and then incubated at 37°C overnight with shaking at 220 rpm. The overnight cells were washed three times and then resuspended in M9 medium (supplemented with 2 mM MgSO_4_, 100 μM CaCl_2_, 30 mM EA, and 200 nM B_12_) to an optical density at 600 nm (OD_600_) of 0.01. A 250-μl culture was growing aerobically at 37°C in a 96-well microplate reader with shaking at 225 rpm, using the System Duetz technology platform (Growth Profiler 960, EnzyScreen). The readings of OD_600_ were taken every 30 min. At least three biological replicates of each growth curve were collected. Lag time was calculated by time to reach OD_600_ = 0.2. Nonlinear regression was used for calculation by the Gompertz growth model.

### Transmission electron microscopy

The structures in the *S.* Typhimurium WT and mutant strains were visualized using thin-section EM. An overnight LB culture was inoculated 1:100 in 5 ml of minimal medium in a 30-ml universal glass vial in the absence of EA and B_12_ shaken at 220 rpm overnight at 37°C. A 500-μl culture was subinoculated to 50 ml of minimal medium in a 250-ml flask in the presence of EA and B_12_, shaken aerobically at 37°C for 5 hours. Bacterial samples were pelleted at 6000*g* for 10 min and fixed twice with 0.05 M sodium cacodylate buffer (pH 7.2) containing 2.5% glutaraldehyde using 100 W for 1 min (P1). Samples were embedded in 4% agarose and then stained three times with 2% osmium tetroxide and 3% potassium ferrocyanide using 100 W for 20 s (P2). The reduced osmium stain was then set using a 1% thiocarbohydrazide solution for 10 min. The second osmium stain was applied using P2 with 2% osmium tetroxide. The sample was made electron dense with 2% uranyl acetate incubated at 4°C overnight. Dehydration was run with a series of increasing alcohol concentrations (30 to 100%) before cell embedding in medium resin. Thin sections of 70 nm were cut with a diamond knife. Images were recorded by a Gatan Rio 16 camera, DigitalMicrograph software, and an FEI 120-kV Tecnai G2 Spirit BioTWIN transmission electron microscope.

### Fluorescence microscopy

The bacterial cells were imaged using a ZEISS Elyra 7 with Lattice SIM^2^ super-resolution microscope. Cells were pelleted and washed twice with phosphate-buffered saline (PBS) buffer, followed by fixing with 4% paraformaldehyde solution (prepared with PBS buffer) and incubated at room temperature for 15 min before imaging. Images were captured in Lattice SIM mode using an SBS LP 560 dual camera beam splitter and a Plan-Apochromat 63×/1.4 oil DIC M27 objective. Two tracks (one for sfGFP imaging excited at 488 nm and the other one for mCherry imaging excited at 561 nm) detected by two cameras were used to avoid fluorescence cross-talk. Two cameras were always aligned using the microscope internal calibration pattern before imaging. Images were captured as 1280 x 1280 pixels at 16 bits. Super-resolution images were obtained by processing under SIM^2^ by Zen software. Additional imaging and processing parameters of light microscopy are listed in table S3.

To monitor the birth event and the progression of the shell and the cargo of Eut BMCs, 5 μl of bacterial culture in minimal medium in the absence of EA and B_12_ was dropped onto the minimal medium plate in the presence of EA and B_12_ and left to dry at 37°C. The agarose plate with cell patches was cut out and attached to a 35-mm glass-bottom dish and covered by a 0.17-mm glass coverslip. The images were taken at 0 min when cells just started to grow on the minimal medium plate in the presence of EA and B_12_ and at either 1- or 3-min intervals to track the assembly of Eut BMCs. Represented images were shown at different time points when important events occurred. The temperature was controlled at 37°C during imaging. All images were captured from at least three biological replicates. A custom Fiji (*68*) pipeline was developed to analyze multichannel fluorescence microscopy image stacks for quantitative and classification purposes. The time series images were preprocessed with bleach correction using an exponential decay fit method (*69*), and sequential time frames were registered to correct for stage jitter (*70*). Individual bacteria were cropped from the registered images and resaved for further analysis. A developed macro then generates a maximum intensity projection (MIP) and sums the signal of both channels. ROIs are identified using a global threshold (*71*) followed by connected component analysis (Analyze Particles, size 0.5 μm^2^-Infinity) and measured across all frames for both channels. The key intensity metrics—including maximum, mean, SD, and kurtosis—are extracted. The resulting measurements are compiled into structured CSV files, which serve as the primary input for downstream analysis in the KNIME Analytics Platform (version 4.7.8) (*72*). A secondary input consists of an Excel file, which includes manually determined frames for each channel where the first microcompartment appears. These two inputs are merged using the file name as a unique identifier. Subsequently, the data are split by channel (magenta and green), and a random forest classifier is trained on each dataset to categorize bacteria as exhibiting either homogeneous fluorescence or punctate signals. The training uses the four extracted intensity-based measurements and the identified first microcompartment appearance frame as input. The trained models are saved for use in classifying new data of similar type. The results are further processed to determine the time frame of the first microcompartment appearance for each channel and input file. The complete workflow, including the training data and trained models, is publicly available on the KNIME Community Hub (https://hub.knime.com/marie_held/spaces/Public/~FiLNvaHabPidBPpt/). Last, the extracted first frames of MC appearance are used to normalize and align the time series maximum intensity data to the penultimate frame preceding microcompartment appearance.

FRAP experiments were performed on a Zeiss LSM780 confocal microscopy as described previously (*23*). Five microliters of bacterial cells that grew in minimal medium in the presence of EA and B_12_ for 5 hours was dropped onto the minimal medium plate and allowed to dry before sandwiching with a 35-mm glass-bottom dish and a 0.17-mm glass coverslip. 100% laser power was applied to bleach a cross-sectional line across the center of the cell. Images were captured every 1 min for 60 min. Fluorescence profiles along the long axis of the cell were obtained by ImageJ software and normalized to the same total fluorescence. The mobile proportion (*M*) was given by

$$M=\frac{\left(\text{Final fluorescence}\right)-\left(\text{Postbleach fluorescence}\right)}{\left(\text{Scaled prebleach fluorescence}\right)-\left(\text{Postbleach fluorescence}\right)}$$

Experimental fluorescence recovery curves were obtained by plotting the fluorescence profile values at the center of the bleached area against time. Then, fluorescence recovery curves were fitted to a single exponential function, given by

$$f\left(t\right)=A\left(1-e^{-\tau t}\right)$$

### Protein expression and isolation

The EPs EutC^1–20^ and EutE^1–20^ were synthesized by GenicBio. Synthetic gene for EutQ (residues 1 to 229; UniProt code: Q9ZFV5) was cloned into pET14b modified with a TEV protease site located before the protein gene sequence. The EutQ N-terminal domain fused with a soluble fusion partner, the immunoglobulin binding domain of streptococcal protein G (GB1) at its C terminus (EutQ^1–99^-GB1), EutQ C-terminal domain (EutQ^100–229^), and GB1 were cloned into the pETM11 expression vector. Hexahistidine Ni^2+^ affinity tag (His-tag) was positioned between EutQ^1–99^ and GB1. For MST, the *eutM*, *eutQ* and *eutL* genes of *S.* Typhimurium LT2 were inserted into pET-22b (+) with either a C-terminal his tag or a strep tag. Different pairs of primers containing restriction enzyme sites (*Nde* I and *Xho* I) and gene-specific sequences were used to amplify the gene fragment, and the gene fragment was ligated to the expression vector pET-22b (+) using a seamless cloning kit. The vector backbone and primers for Gibson assembly cloning are listed in tables S1 and S2.

Heterologous expression of recombinant EutQ, EutQ^1–99^-GB1, EutQ^100–229^, and GB1 were achieved by transforming BL21 (DE3) star cells with the relevant expression plasmid. Cells were plated and incubated overnight on LB agar containing either ampicillin (pET14b) or kanamycin (pETM11) at 37°C in a static incubator. Starting *E. coli* culture were prepared by inoculating 1 ml of LB broth containing the relevant antibiotics was inoculated with one colony picked from the prepared plates and incubating at 210 rpm for 5 hours at 37°C. For protein expression in minimal media, another starting *E. coli* culture were prepared by inoculating 20 ml of M9 media with 100 μl of primary culture in the presence of the relevant antibiotics and incubated overnight at 210 rpm and 37°C. M9 protein expression media for preparing the ^15^N and ^13^C isotopically labeled proteins were prepared with either isotopically labeled ^15^NH_4_Cl for 2D NMR or with both ^15^NH_4_Cl and ^13^C-glucose for 3D NMR studies. Expression culture was then made by adding 20 ml of starting culture into 1 liter of M9 media containing the relevant antibiotics and incubated at 37°C and 210 rpm until the OD_600_ of 0.7 was reached.

Protein expression was induced with 0.8 mM isopropyl-β-d-thiogalactopyranoside (IPTG). The cell culture was incubated overnight at 18°C and 180 rpm, and cells were harvested by centrifugation at 5000 rpm for 30 min at 4°C. Cell pellets of 2-liter culture were resuspended to 5% (w/v) in buffer A [50 mM Na_2_HPO_4_, 300 mM NaCl, 1 mM dithiothreitol (DTT), and 10% glycerol (pH 7.2)] supplemented with DNase1 (15 μg ml^−1^), stood in ice for 20 min, and lysed by one pass through the cell disruptor (Constant Systems, UK) at 19 kpsi (131 MPa) at 4°C. The lysate was centrifuged at 18,000 rpm and 4°C, and the supernatant was collected and passed through a preequilibrated 5-ml FF His-trap column (Cytiva, USA). The column was washed with 5 column volumes (CV) of 5% buffer B (buffer A + 500 mM imidazole) and eluted by a gradient increase in buffer B. Protein samples were further purified by size exclusion chromatography using an S200 Superdex 16/600 GE Healthcare column in a buffer containing 50 mM Na_2_HPO_4_,150 mM NaCl, and 10% glycerol (pH 7.2). These were aliquoted and stored at −80°C. Experimental protein samples were buffer exchanged into 20 mM Na_2_HPO_4_ and 20 mM NaCl (pH 6.5).

For MST, the gene expression plasmid construction was carried out in *E. coli* strain BL21(DE3) at 37°C in LB medium with ampicillin (100 μg ml^−1^). The BL21(DE3) constructs were cultured overnight at 37°C in LB medium with ampicillin (100 μg ml^−1^) to an OD_600_ of 0.8 to 1.0. EutQ and EutL expression were induced at 25°C, and EutM expression was induced at 37°C for 12 to 16 hours with 0.4 mM IPTG. The proteins were first purified with Ni^2+^-NTA resin (GE Healthcare, USA) or Strep-tag XT (IBA Lifescience, Germany), and then gel filtration chromatography was performed on a Superose 6 column or a Superdex G200 column with fast protein liquid chromatography (AKTA purifier 10, GE Healthcare, USA).

### Proteomic analysis

Overnight LB cultures from four single colonies of *S.* Typhimurium LT2 were inoculated 1:100 in 50 ml of minimal medium in a 250-ml flask in the absence of EA and B_12_ shaken at 220 rpm overnight at 37°C. Five milliliters of overnight minimal medium cultures was pelleted, washed, and subinoculated to 100 ml of minimal medium in a 500-ml flask in the presence of EA and B_12_, shaken aerobically at 37°C for 15, 30, 60, and 300 min. Control samples are *S.* Typhimurium growing in the same condition but without EA and B_12_ for 0 and 300 min. Bacterial samples (24 samples in total) were pelleted at 6000*g* for 10 min and stored at −80°C for further analysis.

Samples were lysed in 250 μl of lysis buffer [1% SDS, 1% IGEPAL, 1% sodium deoxycholate, 125 mM NaCl, 5 mM EDTA, and 100 mM Tris buffer (pH 8)] and then boiled at 80°C for 10 min and sonicated using a probe sonicator at 30% amplitude for six cycles of 10-s on and 20-s off. Protein quantification was conducted by BCA assay. Fifty micrograms of protein was diluted to a total volume of 80 μl using 50 mM ammonium bicarbonate (AmBic). Cysteine reduction was performed by adding 5 μl of a DTT solution (11.1 mg/ml; to a final concentration of 4 mM) and incubating at 60°C for 10 min with 600 rpm. Subsequent alkylation was performed by adding 5 μl of a iodoacetamide (46.6 mg/ml; to a final concentration of 14 mM) and incubating for 30 min in the dark. Sixty nanograms of SP3 beads was added followed by 212 μl of ethanol to a total volume of 80% ethanol to bind the protein to the beads. The beads were mixed for 30 min at 1500 rpm. Samples were placed onto a magnetic rack and left to settle for 5 min. The eluent was removed. Samples were washed six times with 200 μl of ethanol, leaving the sample to stand for 5 min before removing the wash solution. In-solution digestion was carried out in 45 μl of 50 mM AmBic with 5 μl of trypsin (0.2 μg/μl) in 50 mM acetic acid and incubated at 37°C overnight with 1500 rpm. Samples were placed onto a magnetic rack and left to settle for 5 min. The solution was removed and retained. Samples were analyzed using an Evosep One liquid chromatography system (Evosep Biosystems, Odense, Denmark) coupled online to a TIMS ToF HT mass spectrometer (Bruker) using the built-in 15 SPD (samples per day) method. Four survey loading analysis was done to determine final loading. Samples were diluted with 0.1% formic acid. The proteomics data have been deposited to the ProteomeXchange Consortium via the PRIDE partner repository with the dataset identifier PXD059040.

### NMR spectroscopy

Uniformly ^15^N labeled recombinant EutQ^1–99^-GB1 used was diluted to a final concentration of 100 μM 600-μl NMR buffer [20 mM Na_2_HPO_4_ (pH 6.5) and 20 mM NaCl] supplemented with 10% (v/v) D_2_O. Binding studies was done by mixing ^15^N-labeled EutQ^1–99^-GB1 or EutQ with unlabeled peptides (either EutC^1–20^ or EutE^1–20^) in a 1:5 molar ratio of EutQ:peptide. All ^15^N-H HSQC NMR experiments were acquired at 298 K on Bruker Avance III 700-MHz spectrometer equipped with TCI cryoprobe [^1^H, ^15^N, ^13^C] with frequency locked on ^2^H_2_O. Water suppression was achieved by excitation sculpting. Protein backbone resonance assignment of uniformly ^15^N, ^13^C-labeled 1 mM EutQ^1–99^-GB1 was achieved using 25% nonuniform sampling of three pairs of 3D triple-resonance experiments: CBCA(CO)NH/CBCANH, HBHA(CO)NH/HBHANH, and HNCO/HN(CA)CO. Data analysis was performed with CcpNMR Analysis version 3.1.1.

### Microscale thermophoresis

MST assays were performed as previously described (*73*). Dyes were incubated with target proteins at a final concentration of 0.25 μM for 30 min at room temperature to label His-tagged target proteins. After mixing target proteins and twofold increased concentrations of nonlabeled legend proteins, the samples were drawn into 16 glass capillaries and were measured using a Monolith NT.115 instrument (Nano Temper Technologies GmbH, Munich, Germany) at 25°C with 60% excitation power and medium light-emitting diode power. For MST analysis of EutM and EutL, the EutM-his and EutL-strep samples were 0.25 and 24 μM, respectively. The signal-to-noise ratio of MST analysis was 23.00. For MST analysis of EutM-his and EutQ-strep, the EutM and EutQ samples were 0.25 and 108 μM, respectively. The signal-to-noise ratio was 28.15. For MST analysis of EutQ-his and EutL-strep, the EutQ and EutL samples were 0.25 and 24 μM, respectively. The signal-to-noise ratio was 23.05. Please note that 24 μM EutL was used as higher concentrations resulted in protein precipitation. The *K*_d_ was calculated and fitted using the Nano Temper Analysis software.

### Isothermal titration calorimetry

Malvern Microcal Peaq Automated fitted with its proprietary software (MicroCal PEAQ_ITC Automated Control Software) was used for the experiment. The instrument was set for 19 injections at 25°C, whereas reference power, initial delay, and stir speed were set for 6 μcal/s, 60 s, and 750 rpm, respectively. The cell and syringe contained 20 μM protein (EutQ/EutQ^1–99^-GB1/EutQ^100–229^/GB1) and 200 to 400 μM EPs, respectively. Peptide to buffer titration was used as the internal control. Both the protein and ligand were diluted in buffers containing 20 mM Na_2_HPO_4_ and 20 mM NaCl (pH 6.5). The start injection volume was 0.4 μl, whereas subsequent injections were 2 μl. MicroCal PEAQ-ITC Analysis Software was used for data evaluation.

### MD simulations

To investigate the interactions between Helix3 and Helix4 of EutQ^1–99^ and EutC^1–20^, MD simulations were performed. To avoid substantial conformational influences from Helix1 and Helix2 of EutQ^1–99^, which might affect the binding site between Helix3/Helix4 and EutC^1–20^, only Helix3/Helix4 and EutC^1–20^ were modeled and simulated during the process. The MD simulations were performed using the Amber22 software package. The protein force field leaprc.protein.ff19SB was applied, and TIP3P water molecules were added within a 15-Å distance from the protein, creating a cubic box with dimensions of 69 Å by 78 Å by 64 Å. Sodium (Na^+^) and chloride (Cl^−^) ions were added to neutralize the system to a concentration of 150 mM, resulting in a solvated system containing 27,344 atoms.

Position restraints of 2 kcal/mol per square angstrom were applied to the backbone atoms of the protein, and energy minimization was conducted for 2000 steps (1000 steps of steepest descent followed by 1000 steps of conjugate gradient). Then, the restraints were removed, and another 2500 steps (1000 steps of steepest descent followed by 1500 steps of conjugate gradient) of energy minimization were performed. Afterward, under control of the Langevin thermostat, the system was gradually heated from 0 to 310 K over 500 ps, with position restraints of 2 kcal/mol per square angstrom applied to the backbone atoms. This was followed by an unrestrained equilibrium simulation for 5 ns under the NVT ensemble [constant number of particles (N), volume (V), and temperature (T)]. Last, a production MD simulation for 500 ns was performed under the NPT [constant number of particles (N), pressure (P), and temperature (T)] ensemble, where the Berendsen barostat maintained the pressure at 1 atm, and a 10-Å cutoff was applied for nonbonded interactions. Electrostatic interactions were calculated using the particle mesh Ewald method. All covalent bonds involving hydrogen atoms were constrained using the SHAKE algorithm.

Trajectory analysis was performed using the cpptraj module in AMBER22. Binding free energies between Helix3/Helix4 of EutQ^1–99^ and EutC^1–20^ were calculated using the Molecular Mechanics/Generalized Born Surface Area (MM/GBSA) method, and entropy was computed using the NMODE module.

### Bioinformatics analysis

According to previous studies, bacteria from 10 genera were reported to contain the *eut* operon: *Citrobacter*, *Clostridium*, *Enterococcus*, *Escherichia*, *Klebsiella*, *Listeria*, *Proteus*, *Salmonella*, *Shigella*, and *Yersinia* (*74*, *75*). To screen the bacterial species that produce the Eut BMCs, the representative genomes of bacterial species from the 10 genera were downloaded from the RefSeq database (ftp://ftp.ncbi.nlm.nih.gov/genomes/). A protein BLAST database was made from the translated CDS of the representative genomes. The protein sequences of the *eut* operon from *S.* Typhimurium LT2 (RefSeq ID: ASM694v2) were queried against the BLAST database using BLASTp v2.5.0+ (*76*). According to the presence of the essential proteins including EutB, EutL, and EutM, 30 representative isolates were found to contain the Eut BMCs (data S1).

To infer the phylogenetic relationship of the 30 bacterial isolates, the core genes of the isolates were extracted using BUSCO v5.2.5 with the “bacteria_odb10” database. From the single-copy orthologs detected by BUSCO, 113 proteins were existing in all the genomes. The orthologs were extracted and then aligned using Mafft v7.475 (*77*). The alignments were then merged using Seqkit v0.15.0 and trimmed with trimAl (*78*). Fasttree v2.1.10 was used to infer the phylogenetic tree using the JTT + Gamma model. The tree was mid-rooted. The identity of Eut proteins summarized from the BLASTp result was visualized with the tree in iTOL (*79*). The sequences of EutQ, EutC, and EutE proteins were aligned by ESPript 3 (*80*).

The structural predictions of EutQ and EutG were generated by AlphaFold 3 (*81*). The sequence logo of EutQ was generated by WebLogo 3 (*82*, *83*). The alignment was generated using the EutQ sequence queried against the NCBI BLAST refseq-select-prot database. Sequences with similarity greater than 80% were selected, resulting in a total of 44 sequences.
