# Supplementary material for: Molecular basis of the biogenesis of a protein organelle for ethanolamine utilization
Source: Sci Adv. 2025 Oct 1;11(40):eadx9774. doi: 10.1126/sciadv.adx9774 (PMC12487888; doi:10.1126/sciadv.adx9774)
Supplement: Supplementary file 1 — Figs. S1 to S33 Tables S1 to S3 Legends for movies S1 and S2 Legend for data S1 [file sciadv.adx9774_sm.pdf]

Science Advances
AAAS

Supplementary Materials for

Molecular basis of the biogenesis of a protein organelle for ethanolamine utilization

Mengru Yang *et al.*

Corresponding author: Lu-Ning Liu, luning.liu@liverpool.ac.uk

Sci. Adv. **11**, eadx9774 (2025)
DOI: 10.1126/sciadv.adx9774

The PDF file includes:

Figs. S1 to S33
Tables S1 to S3
Legends for movies S1 and S2
Legend for data S1

Other Supplementary Material for this manuscript includes the following:

Movies S1 and S2
Data S1

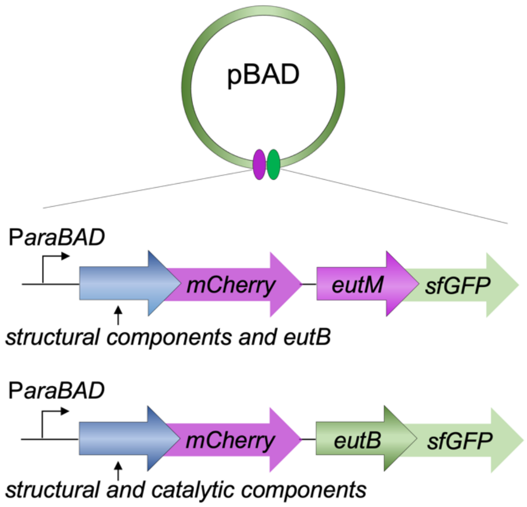

Fig. S1. Constructed vectors to visualize the locations of Eut proteins. Eut proteins are visible by dual-labeling with fluorescence proteins (mCherry and sfGFP). Two series of vectors were constructed. First, EutM (a shell protein) was labeled with sfGFP, and other structural components or EutB (an enzymatic component) were labeled with mCherry. Second, EutB was labeled with sfGFP, and the structural components or other catalytic components were labeled with mCherry.

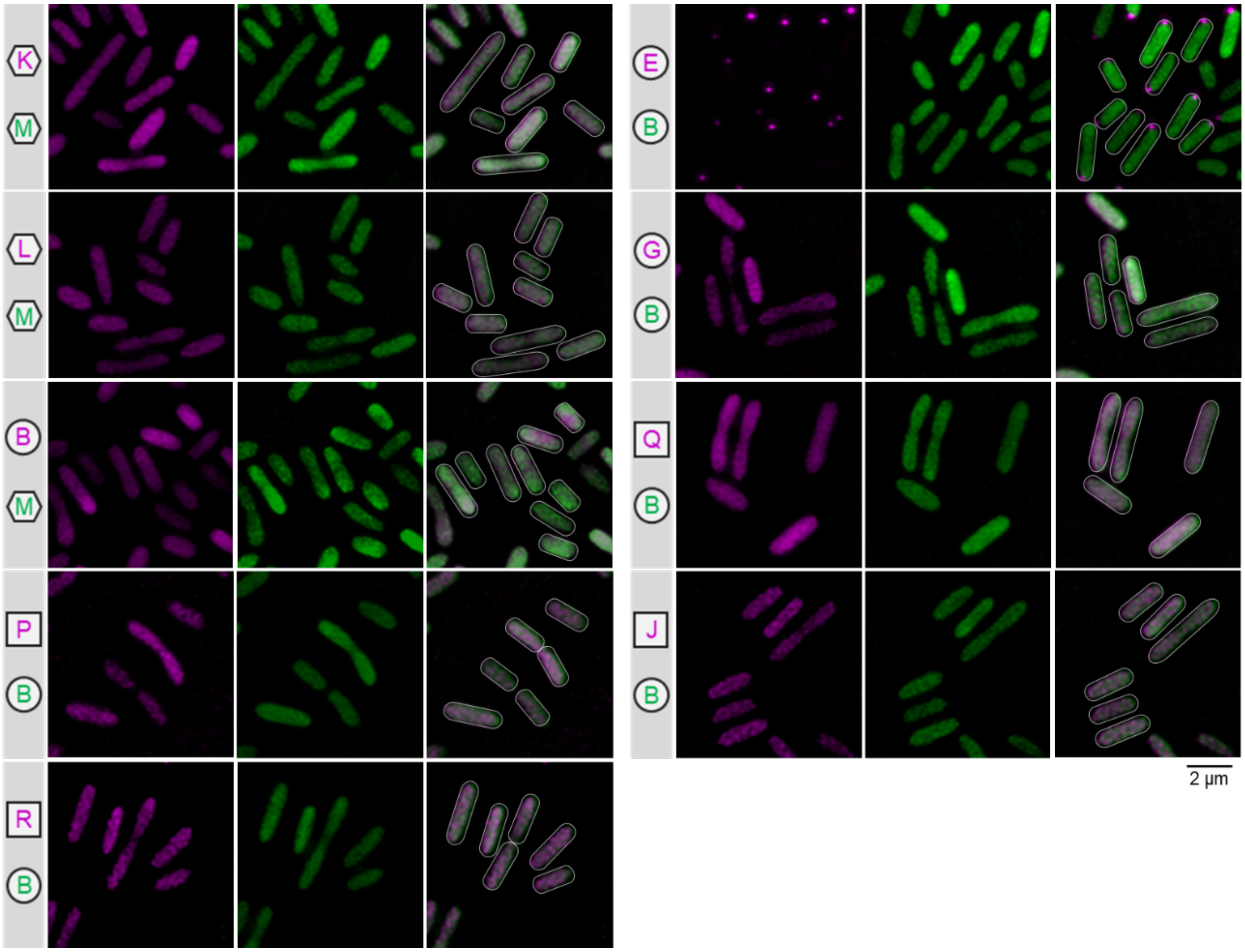

Fig. S2. Eut BMCs are not formed in the absence of EA and B_{12}. *S*. Typhimurium carrying pBAD to express Eut proteins tagged with mCherry and sfGFP, was growing in minimal medium in the absence of EA and B_{12}. EutE aggregated at the cell pole, likely due to its encapsulation peptides at the N-terminus *(26)*.

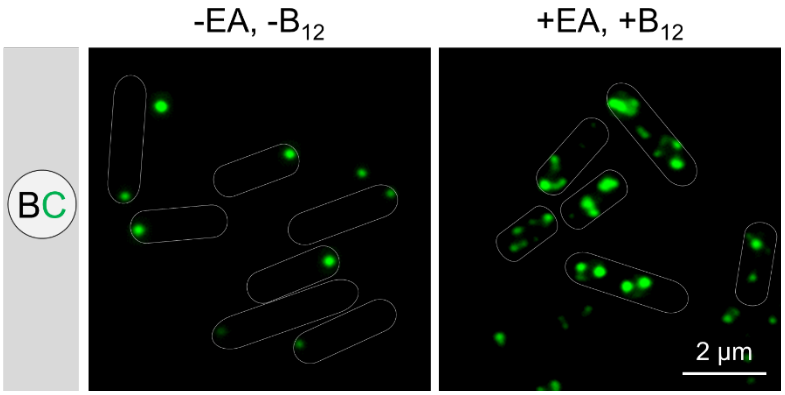

Fig. S3. The distribution of EutBC in the absence or presence of EA and B_{12}. *S.* Typhimurium carrying pBAD to express EutB and EutC-sfGFP, was growing in minimal medium in the absence (left) or presence (right) of EA and B_{12}. EutBC aggregated at the cell pole in the absence of EA and B_{12}, likely due to its encapsulation peptides at the N-terminus *(25)*.

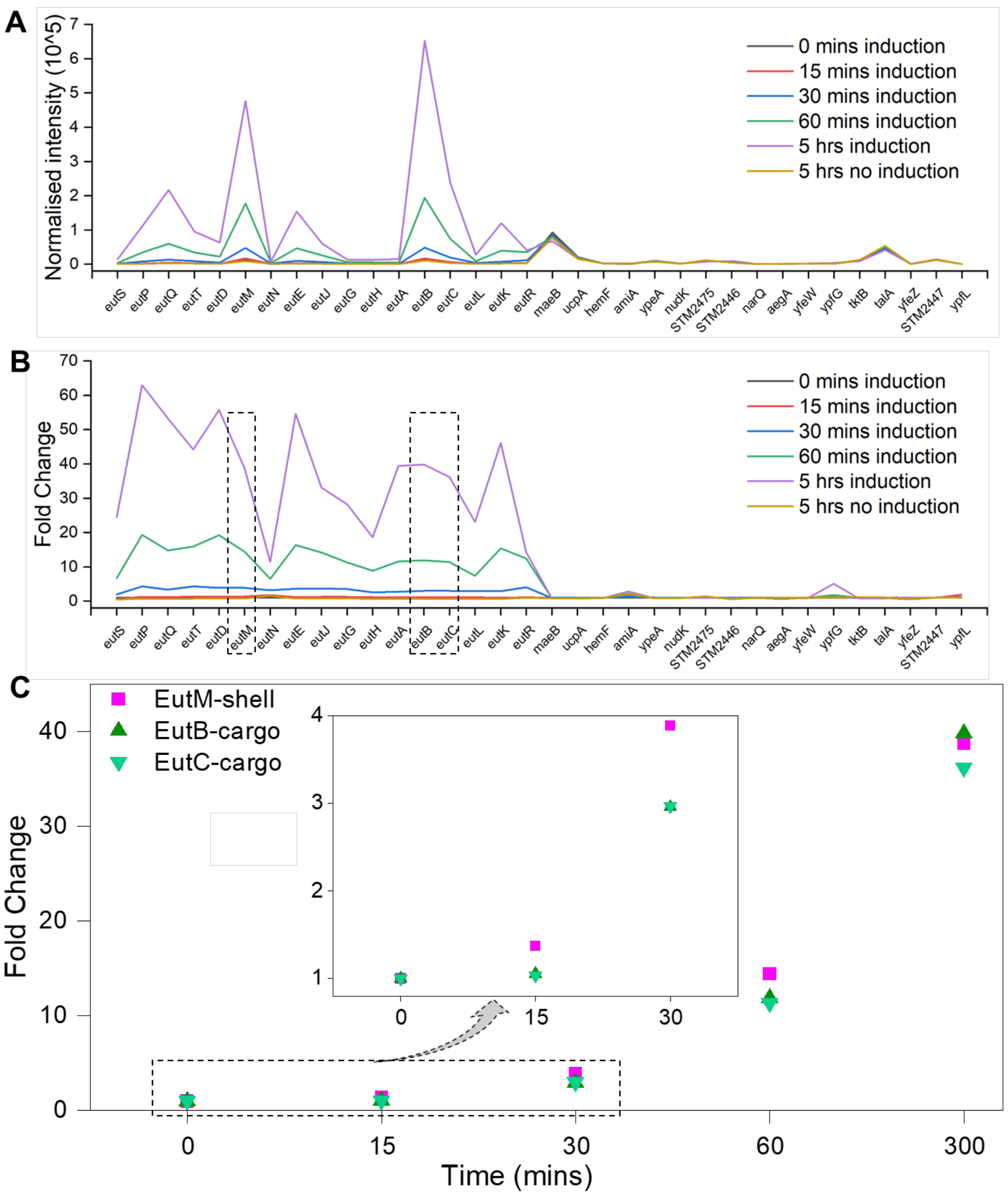

Fig. S4. Proteomics analysis of Eut proteins and potential ancillary proteins involved in EA utilization. A, Normalized intensity of all detected proteins. **B**, Fold changes in protein levels relative to non-induced conditions. Cells were grown aerobically in NCE medium supplemented with 10% succinate and 1 mM $MgSO_4$, with or without 30 mM EA and 200 nM vitamin B_{12}. **C**, Fold change of selected Eut proteins (as indicated in the frames in **B**) at different time points following EA and B_{12} induction, analyzed by mass spectrometry. After 60 minutes of induction, the fold change of the Eut shell protein EutM is higher than that of Eut cargo proteins EutBC. After 300 minutes of induction, the fold changes between the shell protein and cargo proteins become similar. Bacteria were collected at various time points, and whole cell lysates were analyzed using proteomics. Protein levels were compared to those at time 0, prior to induction.

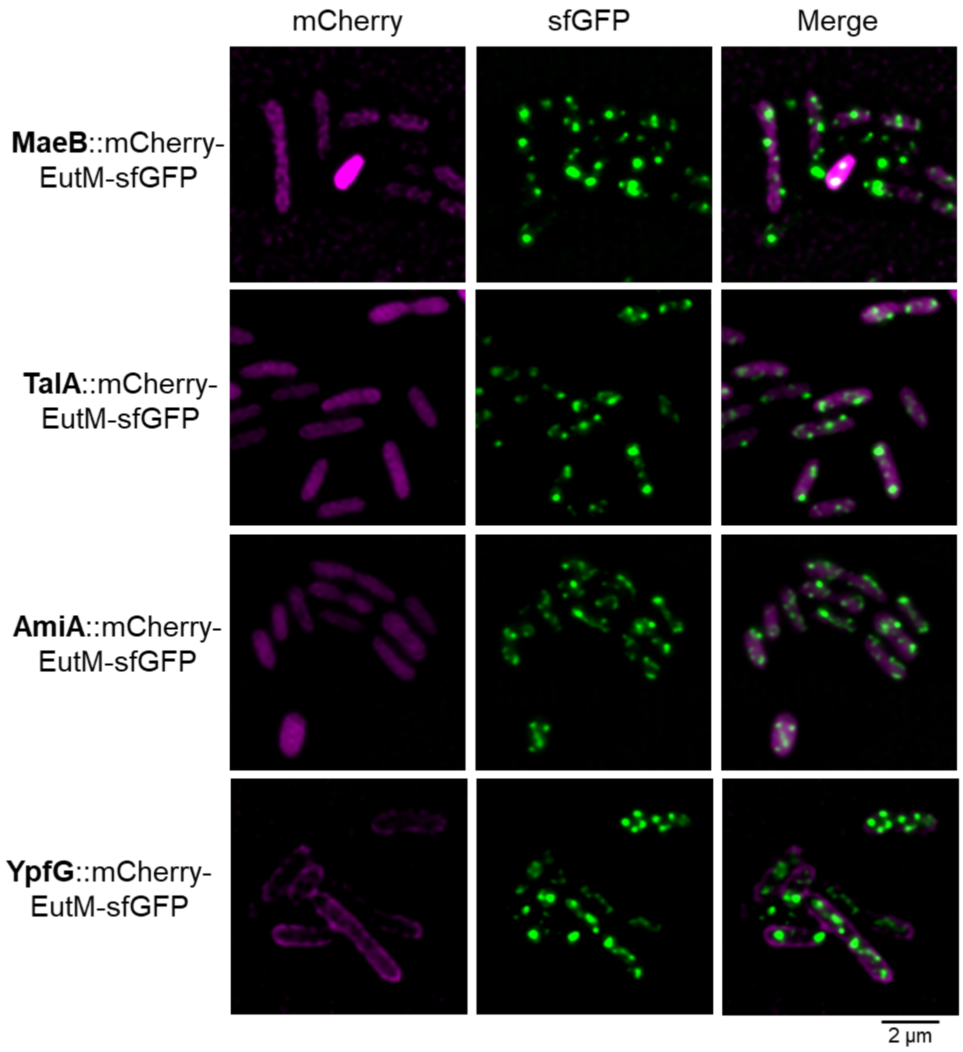

Fig. S5. Localization of potential ancillary proteins (MaeB, TalA, AmiA, YpfG) for Eut BMC biogenesis. *S*. Typhimurium LT2-WT carrying pBAD to express MaeB-, TalA-, AmiA- or YpfG-mCherry and EutM-sfGFP, were growing in minimal medium in the presence of EA and B_{12}.

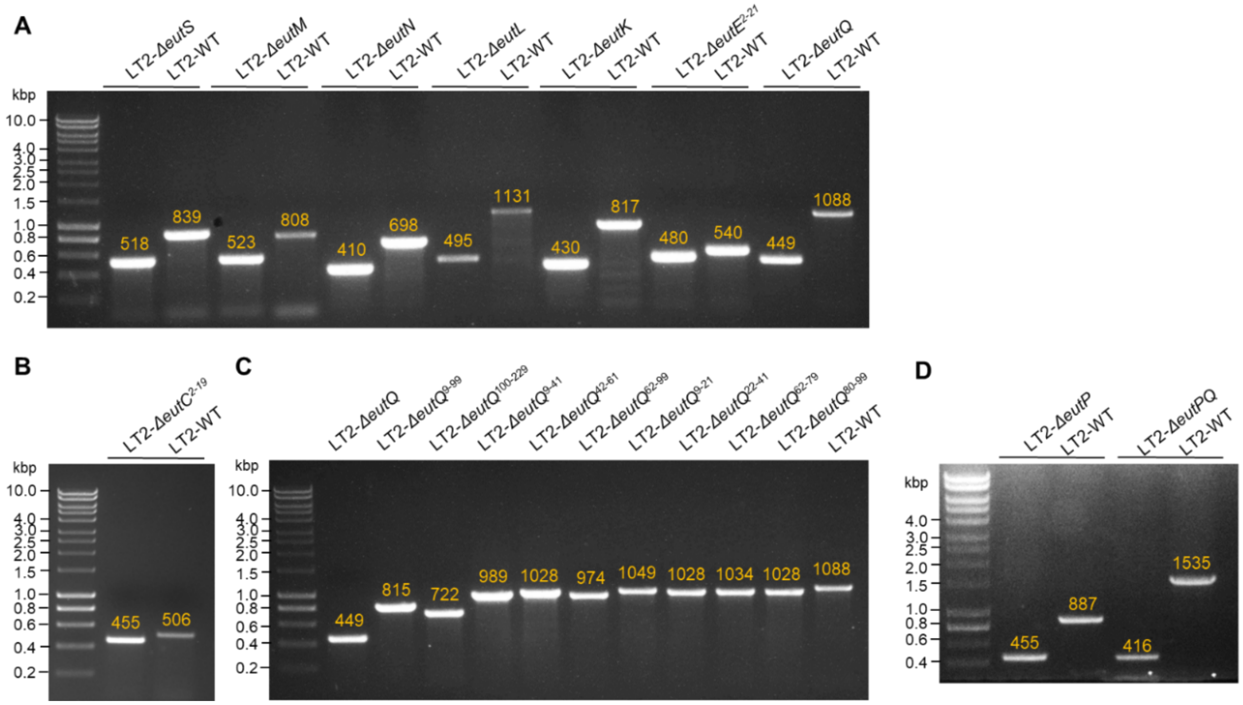

Fig. S6. PCR-based verification of the generation of gene deletion mutants in *S.* Typhimurium LT2. A, *eut* shell gene deletion mutants, Δ$euE^{2\text{-}21}$ and Δ*eutQ*. **B**, Δ$eutC^{2\text{-}19}$. **C**, *eutQ* related gene deletion mutants. **D**, Δ*eutP* and Δ*eutPQ*. The sizes of the PCR products are indicated (bp, yellow).

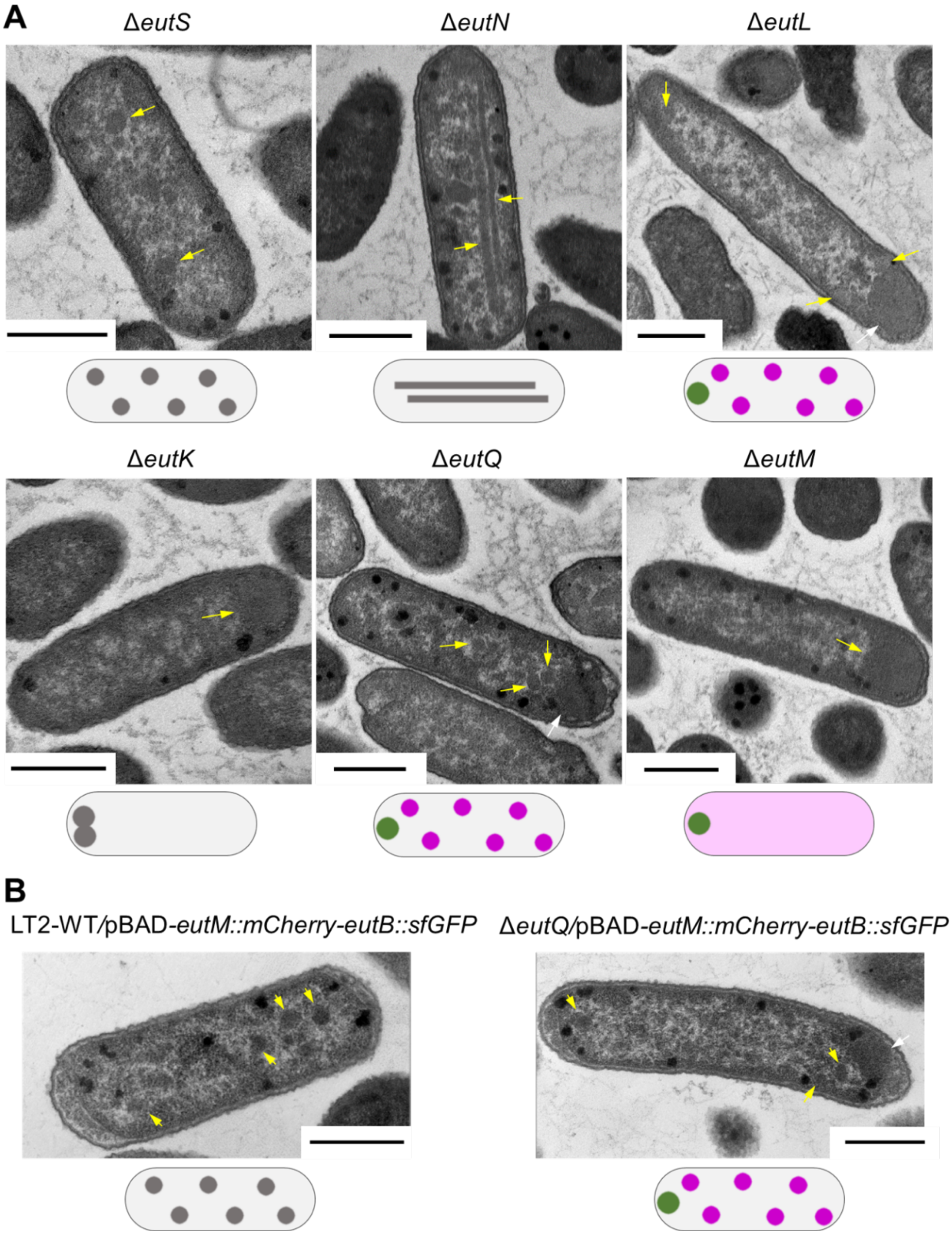

Fig. S7. Thin-section EM verified the observations from the fluorescence imaging. A, The strains used for thin-section EM were not transformed with any fluorescent-labeled vectors. **B,** The strains used for thin-section EM were *S.* Typhimurium WT and Δ*eutQ* mutant carrying pBAD to express EutM and EutB tagged with mCherry and sfGFP, respectively. Cells were growing in minimal medium in the presence of EA and B_{12}. Schematic models of the *in vivo* localization of shell and cargo assemblies observed from fluorescence imaging were shown at the bottom for comparison. Scale bar: 500 nm.

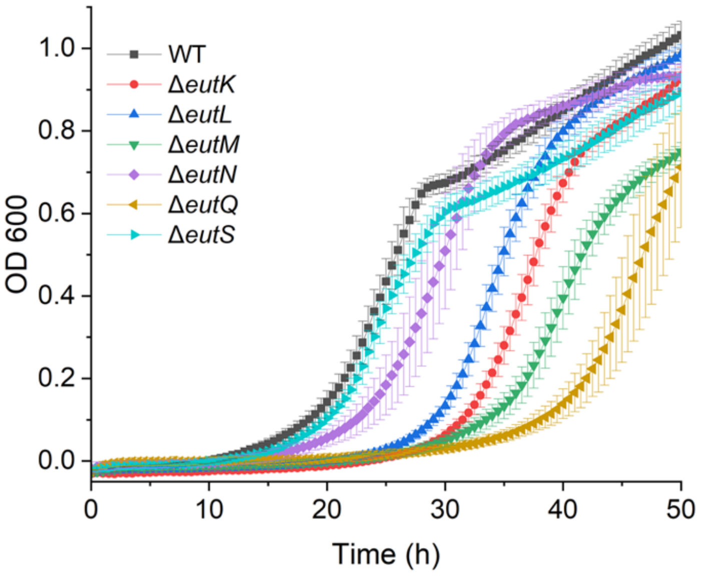

Fig. S8. Growth curves of the *S.* Typhimurium WT and the *eut* gene deletion mutants. Cells were grown under aerobic conditions in M9 medium, which contains 30 mM EA, 200 nM vitamin B_{12}, 2 mM $MgSO_4$, and 100 µM $CaCl_2$.

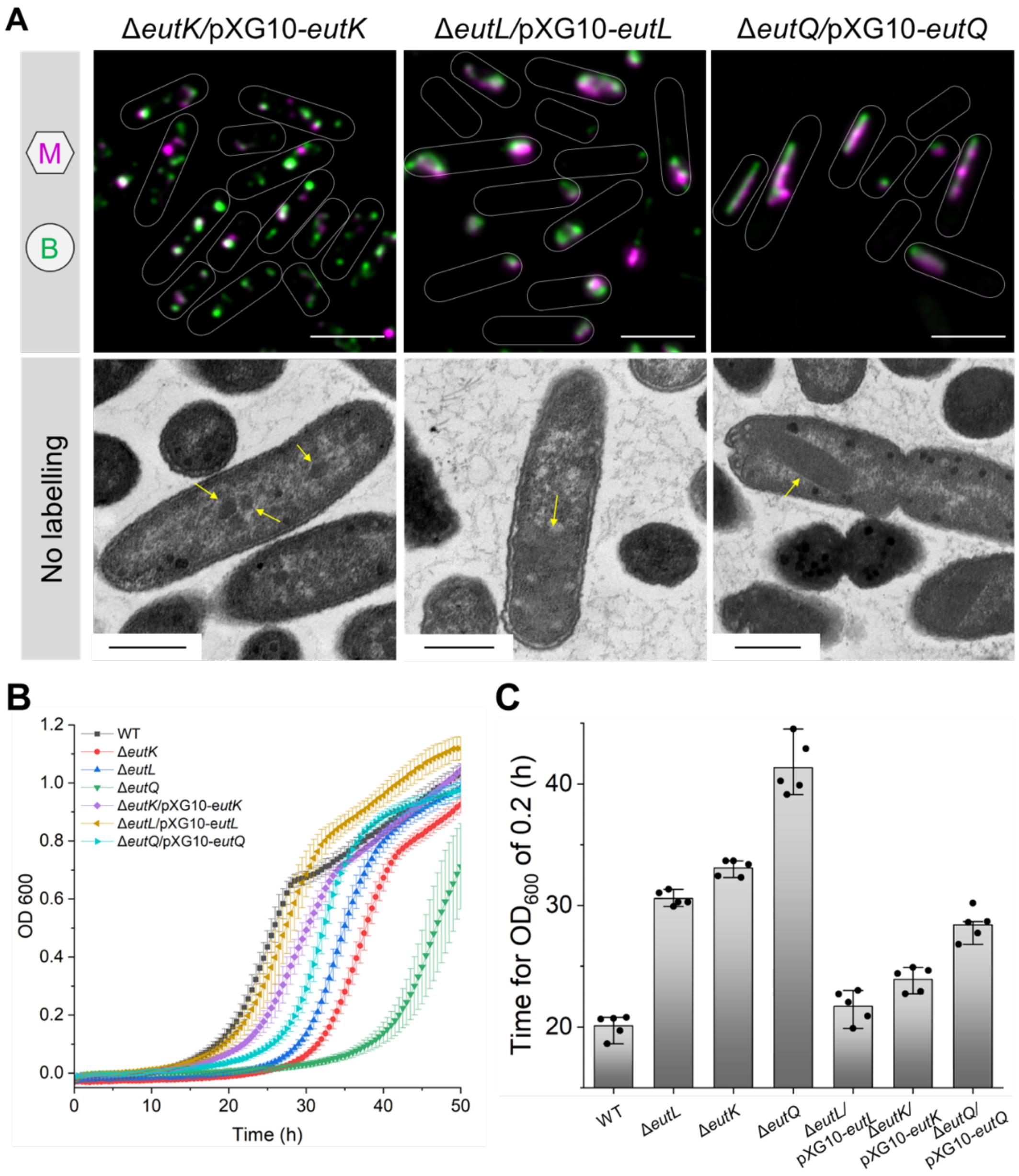

Fig. S9. Successful complementation of EutK, and partial complementation of EutL and EutQ. A, Fluorescence imaging and thin-section EM on gene deletion mutants expressing deleted proteins from pXG10 plasmid growing in minimal medium in the presence of EA and B_{12}. Scale bar in fluorescence images: 2 µm. The Eut BMC structures are indicated with yellow arrows. Scale bar in EM images: 500 nm. **B**, Growth assay curves on gene deletion mutants expressing deleted proteins from pXG10 plasmid grown on EA in M9 medium compared with WT and related mutants. **C**, Time for LT2 WT and mutants to grow to OD_{600}=0.2 on EA and B_{12} in M9 medium (n = 5). The center for error bars represents the mean. The whiskers extend to the smallest and largest data points that are within 1.5 times the interquartile range of the upper and lower quartiles. n number of biologically independent experiments.

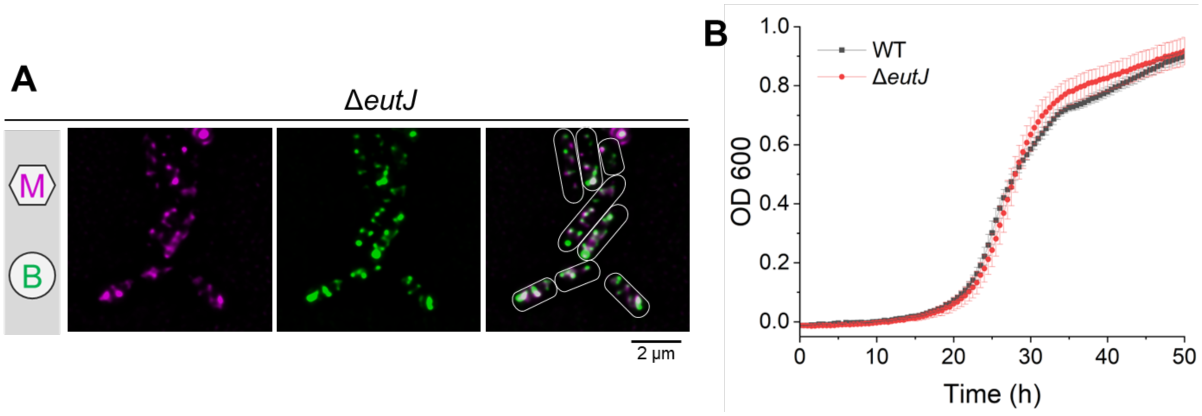

Fig. S10. EutJ is not essential for the assembly of Eut BMCs. A, Localization of EutM-mCherry (shell) and EutB-sfGFP (cargo) in the gene deletion mutants in the absence of EutJ, which were grown in the presence of EA and B_{12}. **B**, Growth curves of the *S*. Typhimurium WT and Δ*eutJ* mutant. Cells were grown under aerobic conditions in M9 medium, which contains 30 mM EA, 200 nM vitamin B_{12}, 2 mM $MgSO_4$, and 100 µM $CaCl_2$.

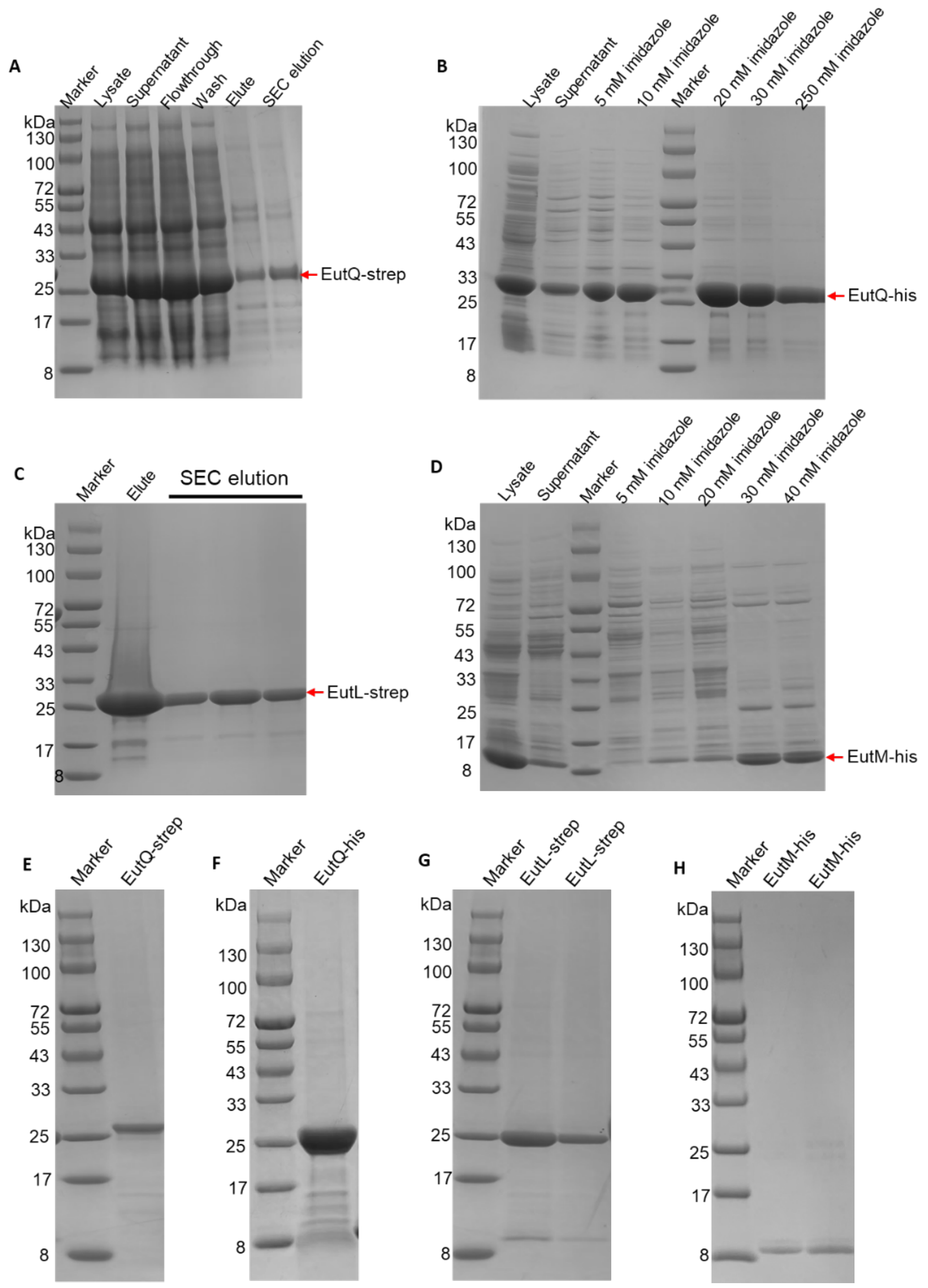

Fig. S11. Purification of EutQ-strep (A & E), EutQ-his (B & F), EutL-strep (C & G), and EutM-his (D& H). SEC: Size exclusion chromatography. A His-tag or Strep-tag was fused to the C-termins of these proteins.

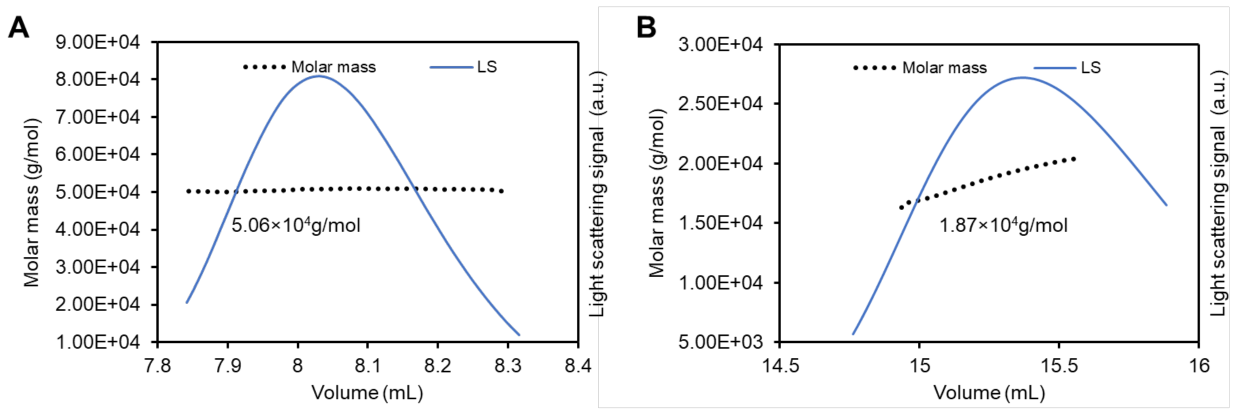

Fig. S12. **Molecular mass analysis by SEC-MALS with the fitting trace presented. A**, EutQ analysis by SEC-MALS revealed a ~50 KDa protein dimer (the molecular weight of a monomer is ~25 KDa). **B**, SEC-MALS analysis of EutQ$^{1\text{-}99}$ fused with GB1 revealed a ~19 KDa protein (expected molecular weight: 19.64 KDa), indicating that the dimerization of EutQ was mediated by the contacts between two EutQ C-termini.

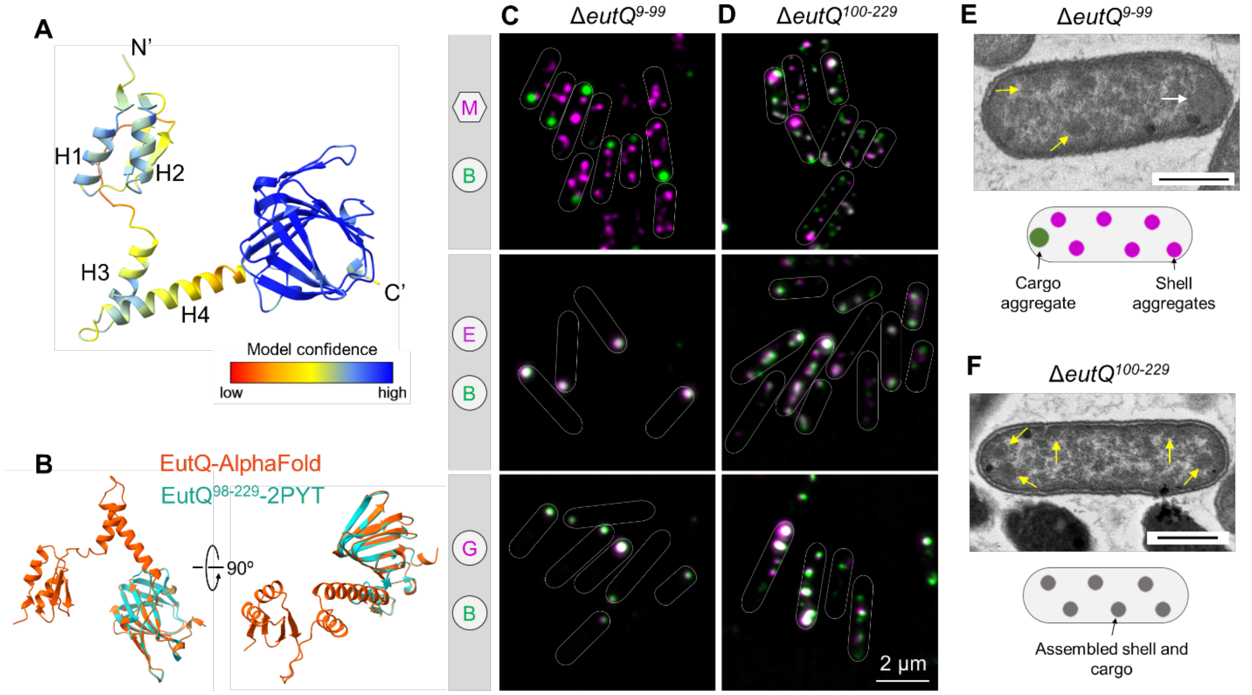

Fig. S13. The N-terminus of EutQ binds the enzymatic core to the shell of EutBMC. A, The structure of EutQ predicted by AlphaFold3. **B**, The structure of EutQ predicted by AlphaFold3 (orange) aligned to the crystal structure of the C-terminus of EutQ (cyan, PDB ID: 2PYT). **C** and **D**, The location of shell protein EutM and different catalytic components were visualized Δ*eutQ*$^{9\text{-}99}$ and Δ*eutQ*$^{100\text{-}229}$ growing in the presence of EA and B_{12}. **E** and **F**, Thin section EM on Δ*eutQ*$^{9\text{-}99}$ and Δ*eutQ*$^{100\text{-}229}$ growing in the presence of EA and B_{12}. Schematic models of the *in vivo* localization of shell and cargo assemblies observed from fluorescence imaging (Fig. 3B, Figs S13C, S13D) were shown at the bottom for comparison. White arrow indicates polar aggregate. Scale bar: 500 nm.

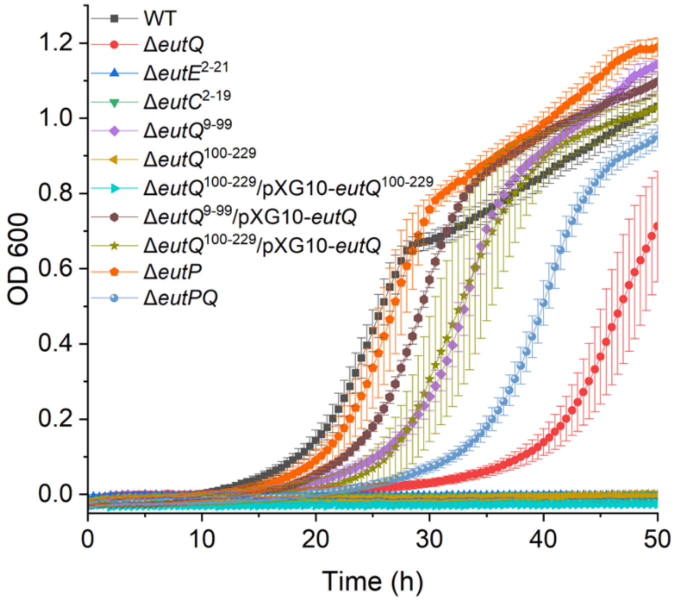

Fig. S14. Growth curves of the *S.* Typhimurium WT, the encapsulation peptides deletion mutants, and *eutQ*-related mutants. The medium is M9 medium, containing 30 mM EA, 200 nM vitamin B_{12}, 2 mM $MgSO_4$, and 100 µM $CaCl_2$. Growth curves were measured on a Growth Profiler 960 (EnzyScreen) under aerobic conditions.

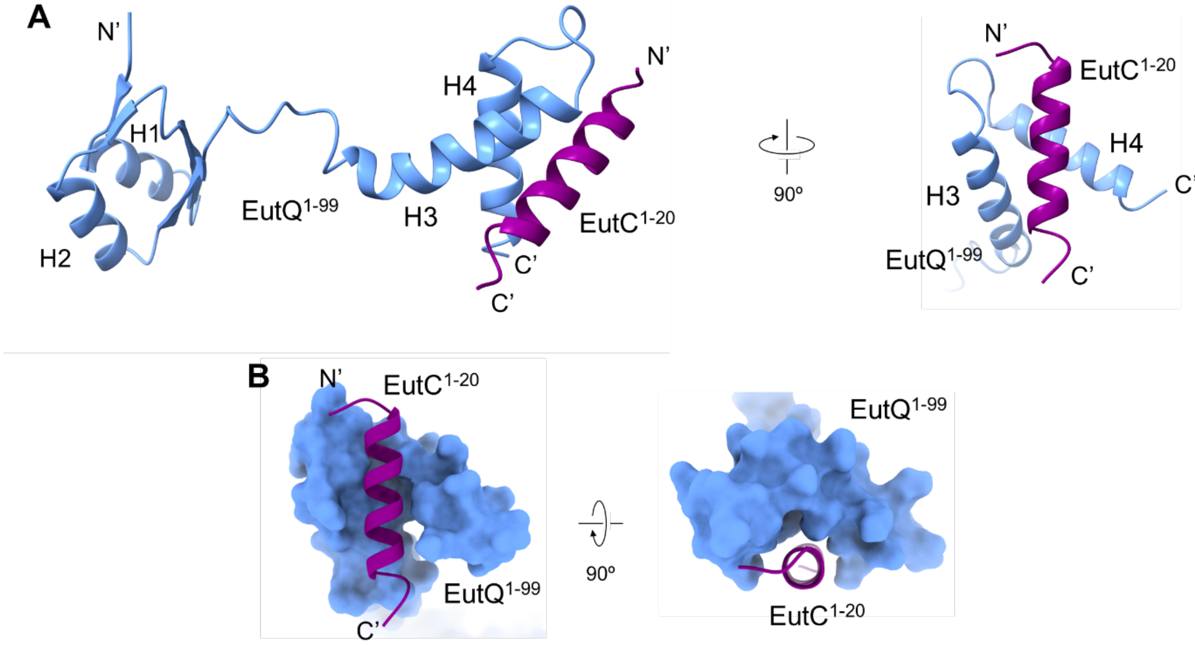

Fig. S15. Interactions between EutQ N-terminus and EutC$^{1\text{-}20}$ predicted by AlphaFold. A, EutC$^{1\text{-}20}$ binds to the H3 and H4 α-helices of EutQ$^{1\text{-}99}$. **B,** Surface presentation of the binding pocket formed by the H3 and H4 α-helices of EutQ$^{1\text{-}99}$ to EutC$^{1\text{-}20}$.

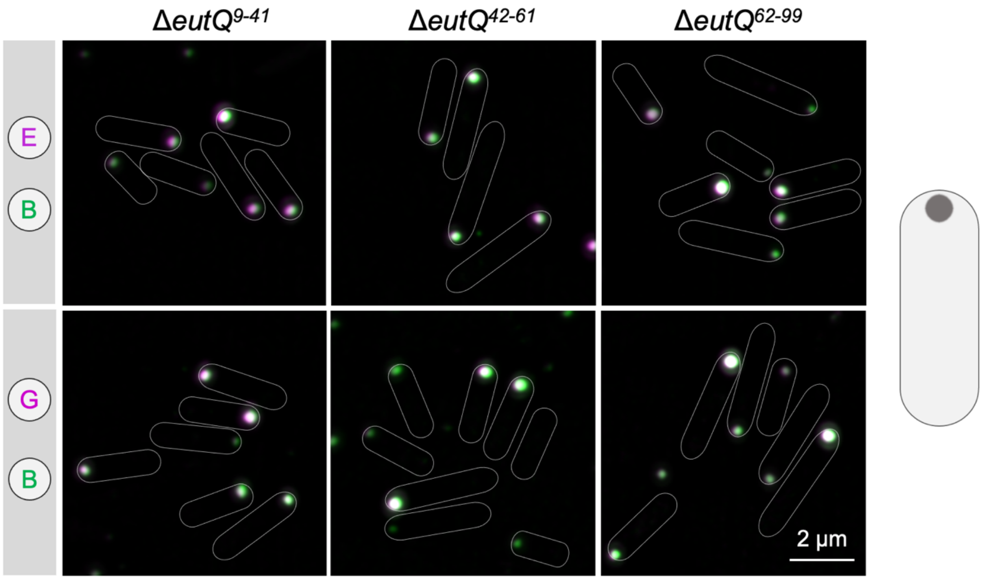

Fig. S16. The location of the cargo proteins of Eut BMCs in different gene deletion mutants of *eutQ*. EutE-mCherry/EutB-sfGFP and EutG-mCherry/EutB-sfGFP were visualized in different mutants following growth in minimal medium in the presence of EA and B_{12}.

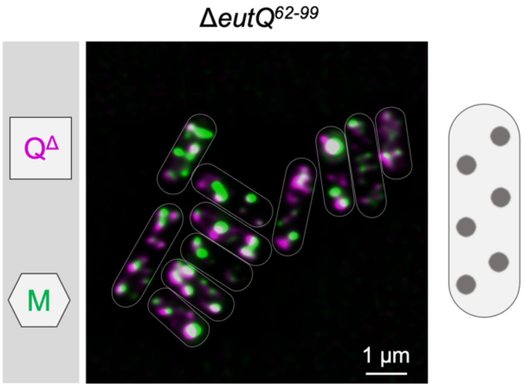

Fig. S17. EutQ$^{\Delta 62\text{-}99}$ assembled with the shell in Δ*eutQ*$^{62\text{-}99}$. EutQ$^{\Delta 62\text{-}99}$-mCherry and EutM-sfGFP (shell) were visualized in Δ*eutQ*$^{62\text{-}99}$ in the presence of EA and B_{12}.

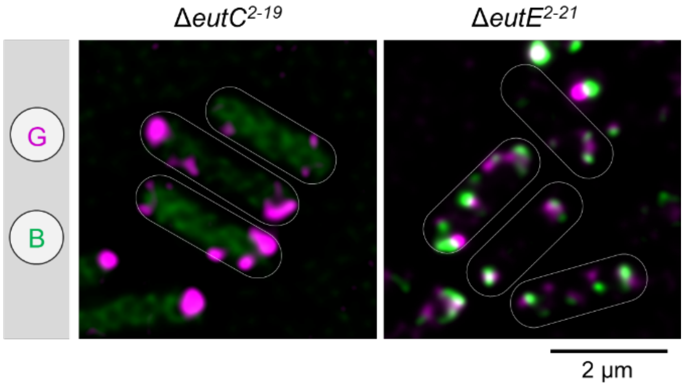

Fig. S18. The location of EutG in EP-deletion mutants. EutG-mCherry and EutB-sfGFP were visualized in Δ*eutC*$^{2\text{-}19}$ and Δ*eutE*$^{2\text{-}21}$ following growth in minimal medium in the presence of EA and B_{12}.

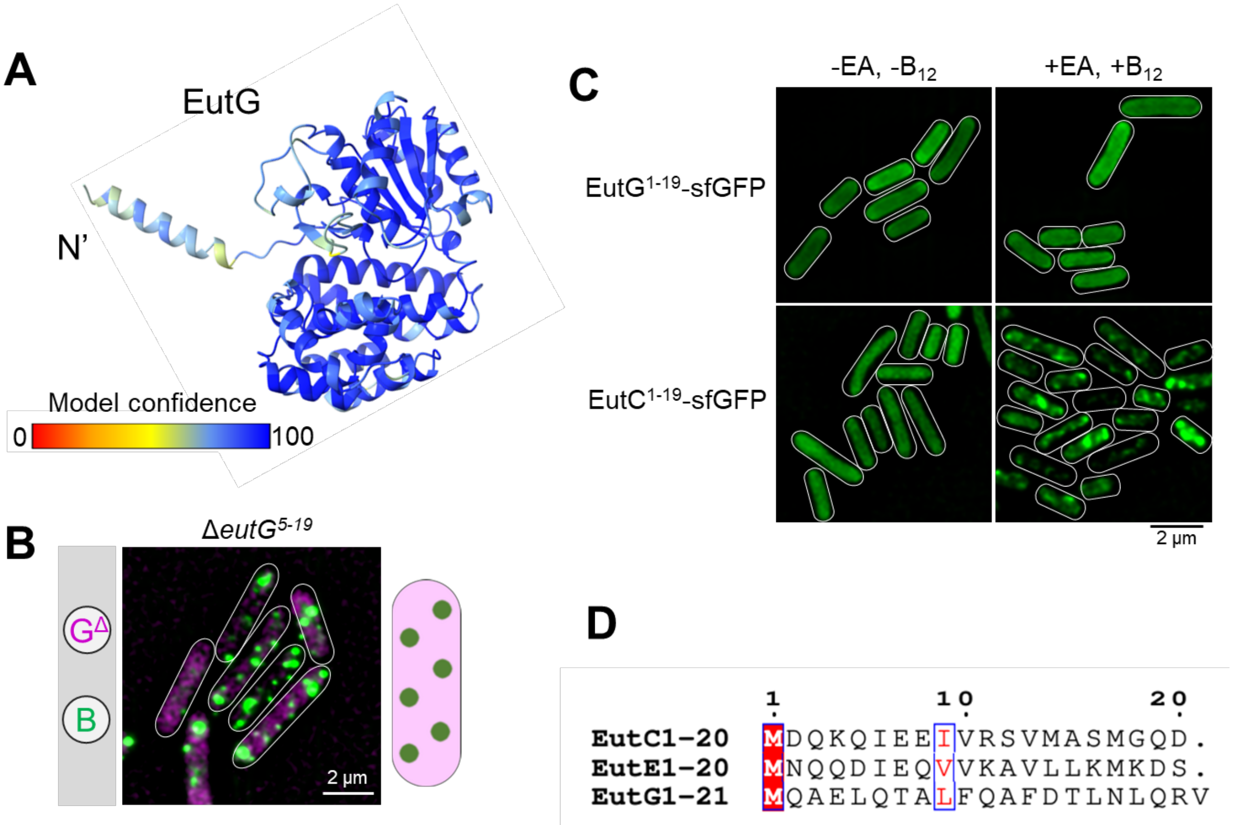

Fig. S19. The role of the N-terminal extension of EutG. A, AlphaFold predicted structures of EutG (Uniprot ID: P41795). **B**, $EutG^{\Delta}$-mCherry/EutB-sfGFP were visualized in $\Delta eutG^{5\text{-}19}$ following growth in minimal medium in the presence of EA and B_{12}. $EutG^{\Delta}$ represents the C-terminus of EutG with $EutG^{5\text{-}19}$ deleted. **C**, The N-terminus of EutG ($EutG^{1\text{-}19}$) is not sufficient to target sfGFP to the Eut BMC. As a control, the known EP, EutC1-19 can target sfGFP to the Eut BMC. *S.* Typhimurium LT2-WT carrying pBAD to express $EutG^{1\text{-}19}$-sfGFP or $EutC^{1\text{-}19}$-sfGFP, were growing in minimal medium in the absence and presence of EA and B_{12}. **D**, Sequence alignment of $EutG^{1\text{-}21}$ with EPs ($EutC1^{\text{-}20}$ and $EutE^{1\text{-}20}$) reveals no significant similarity.

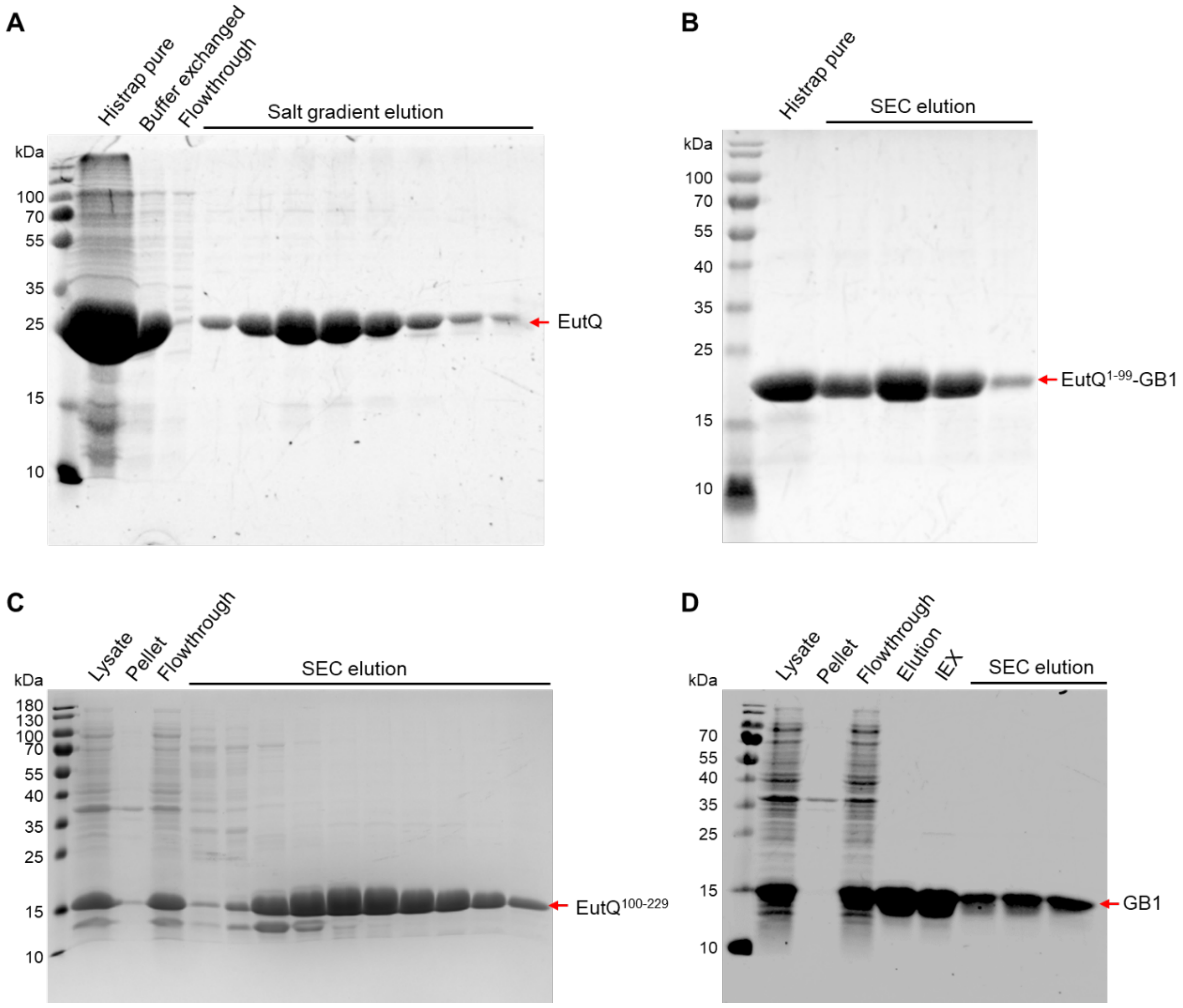

Fig. S20. Purification of EutQ (A), EutQ$^{1-99}$-GB1 (B), EutQ$^{100-229}$ (C), and GB1(D). IEX: Ion exchange chromatography; SEC: Size exclusion chromatography.

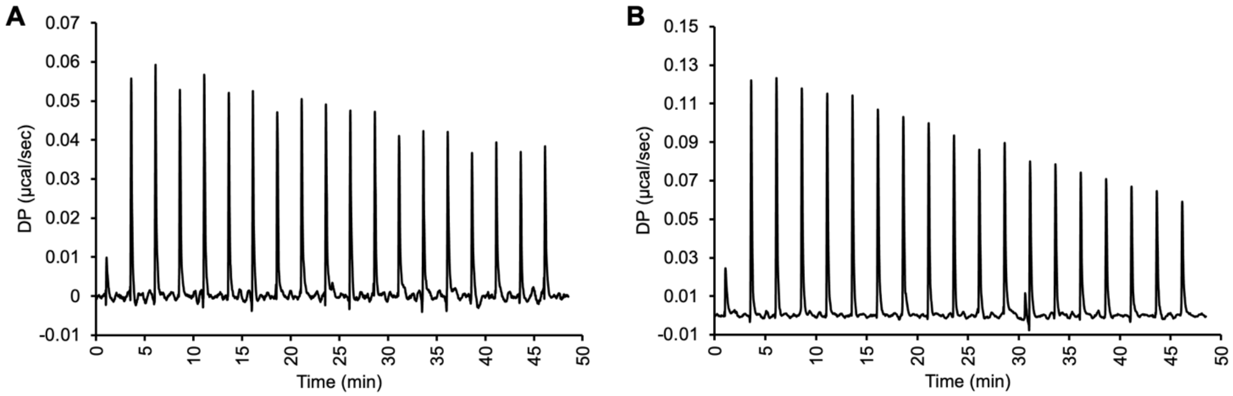

Fig. S21. **ITC isotherm for controls. A**, EutC$^{1-20}$ to GB1 titration. **B**, EutC$^{1-20}$ to buffer titration. There was no binding in both experimental procedures, hence, no calculated best fit curve.

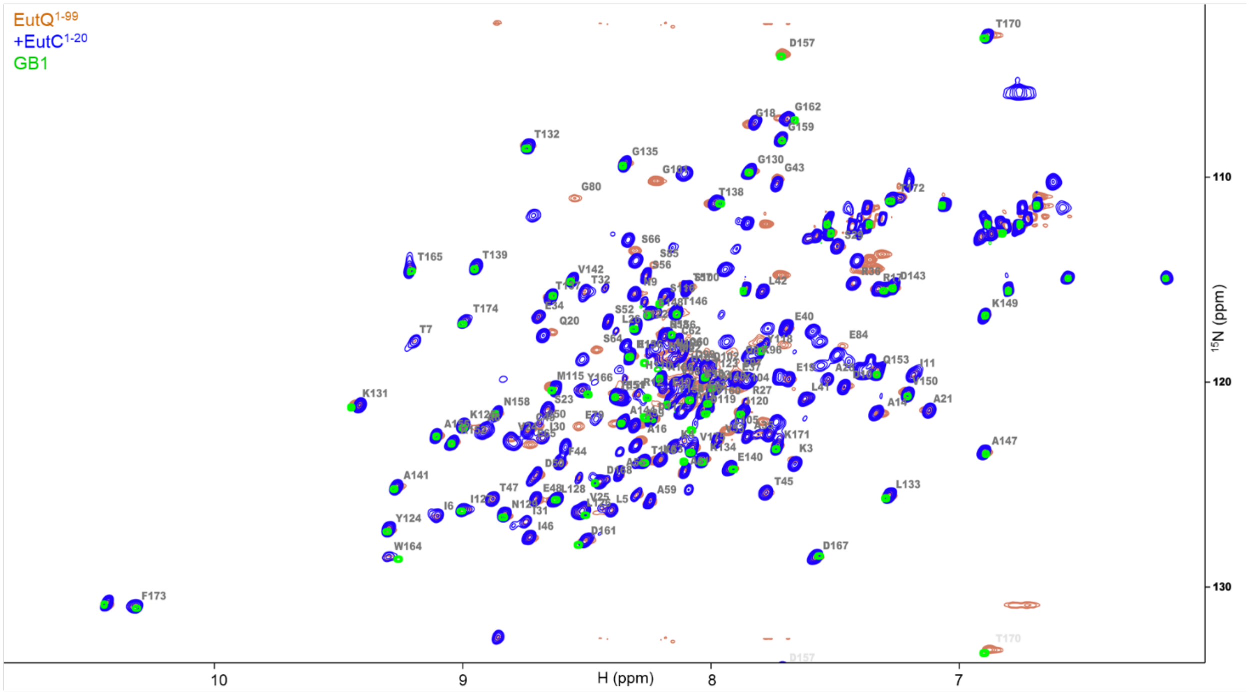

Fig. S22. Backbone assigned 2D ^{15}N-^{1}H HSQC spectrum of uniformly ^{15}N-labeled EutQ$^{1-99}$-GB1 (red) (100 mM, pH 6.5, 298 K) overlaid with (i) ^{15}N-^{1}H HSQC spectrum of ^{15}N uniformly GB1 (green), and (ii) ^{15}N EutQ$^{1-99}$-GB1 in the presence of 500 mM unlabeled EutC$^{1-20}$ (blue). The chemical shift perturbations that were observed in EutQ$^{1-99}$-GB1 without equivalent shifts on GB1 in the presence of ligand are consistent with specific interactions occurring between the EutQ$^{1-99}$ alone and EutC$^{1-20}$. Note that the numbers of residues shown in this figure are shifted by 1 residue due to an additional residue at position 1 (start codon ATG for methionine).

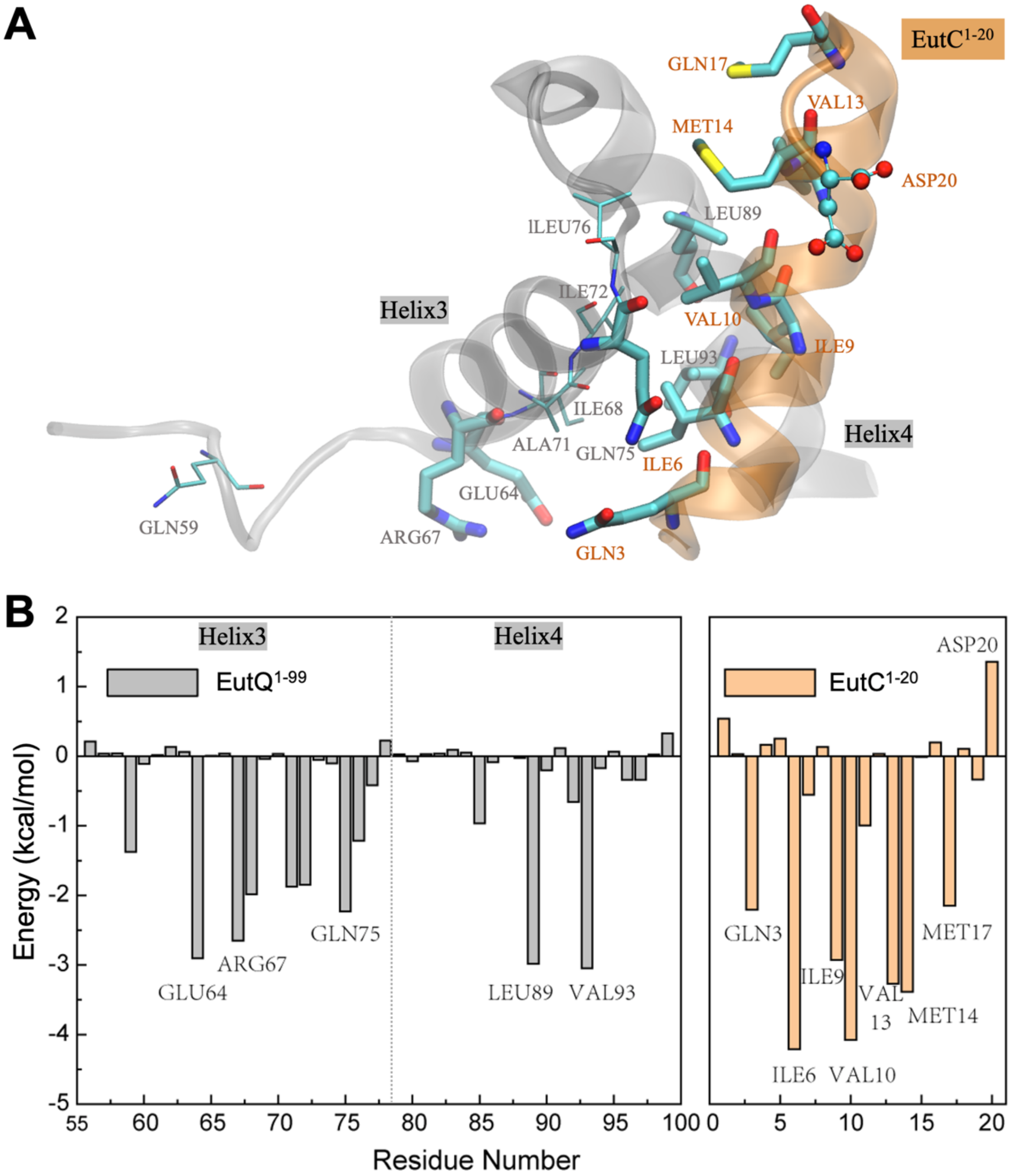

Fig. S23. **Molecular dynamics simulations of the interaction between EutQ$^{1-99}$ and EutC$^{1-20}$. A**, Free energy decomposition calculation of EutQ$^{1-99}$ (grey) and EutC$^{1-20}$ (brown). Residues contributing more than 2 kcal mol^{-1} are shown in thick licorice representation, while residues contributing between 1 and 2 kcal mol^{-1} are shown in thin licorice representation. Residues that generate significant repulsive interactions during the binding process are displayed using the CPK representation. **B**, The figure highlights residues with contributions greater than 2 kcal mol^{-1} and those with repulsion greater than 1 kcal mol^{-1}.

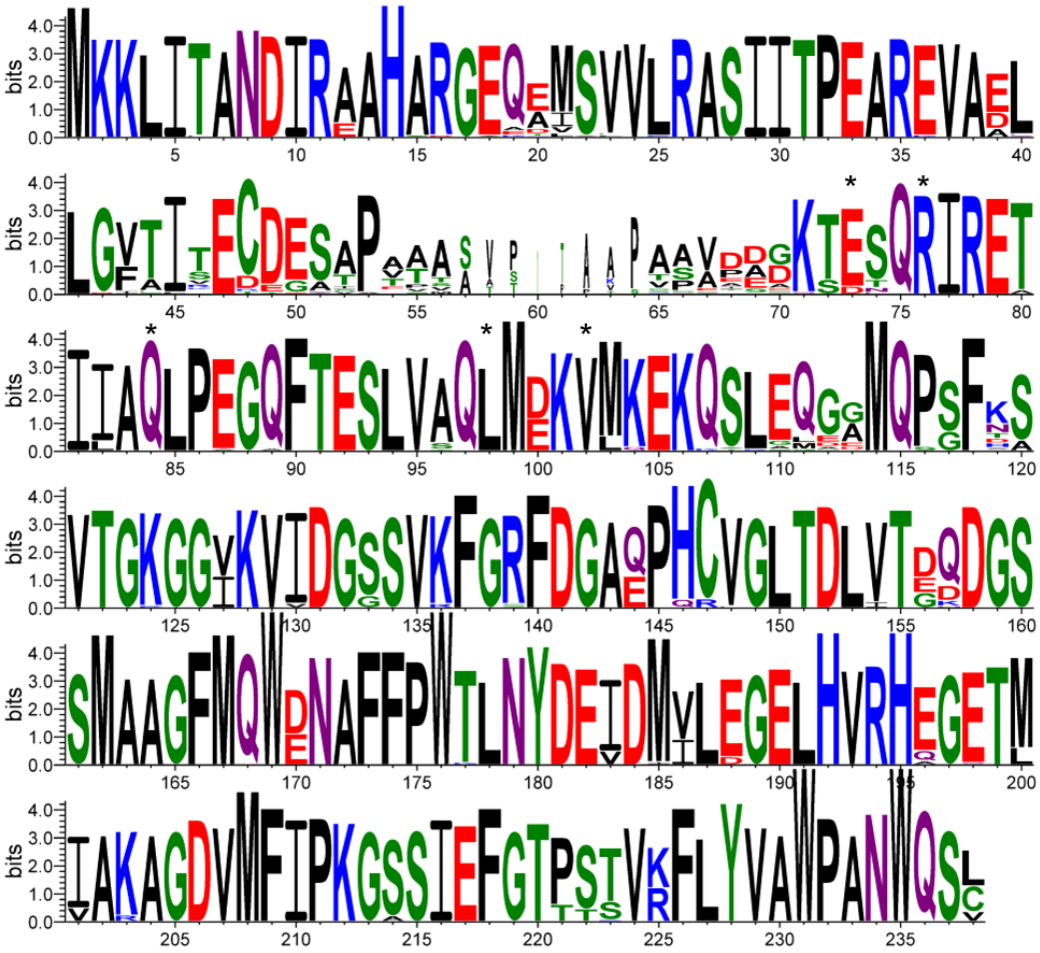

Fig. S24. **Sequence conservation logo for EutQ.** The alignment was generated using the EutQ sequence queried against the NCBI BLAST refseq-select-prot database. Sequences with similarity greater than 80% were selected, resulting in a total of 44 sequences. Residues marked with an asterisk indicate key residues involved in the EutQ and EutC$^{1\text{-}20}$ interaction, as identified by MD simulations depicted in Fig. S23.

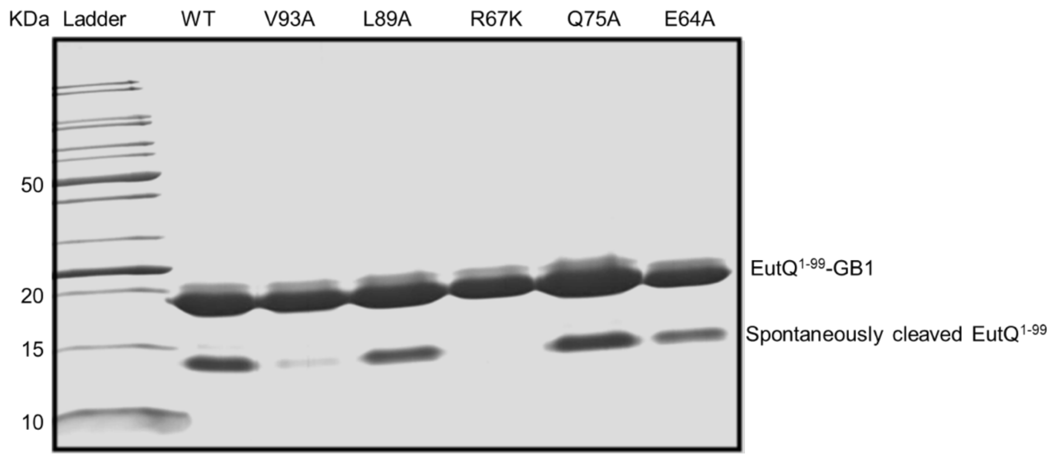

Fig. S25. Purification of various EutQ$^{1\text{-}99}$-GB1 mutants.

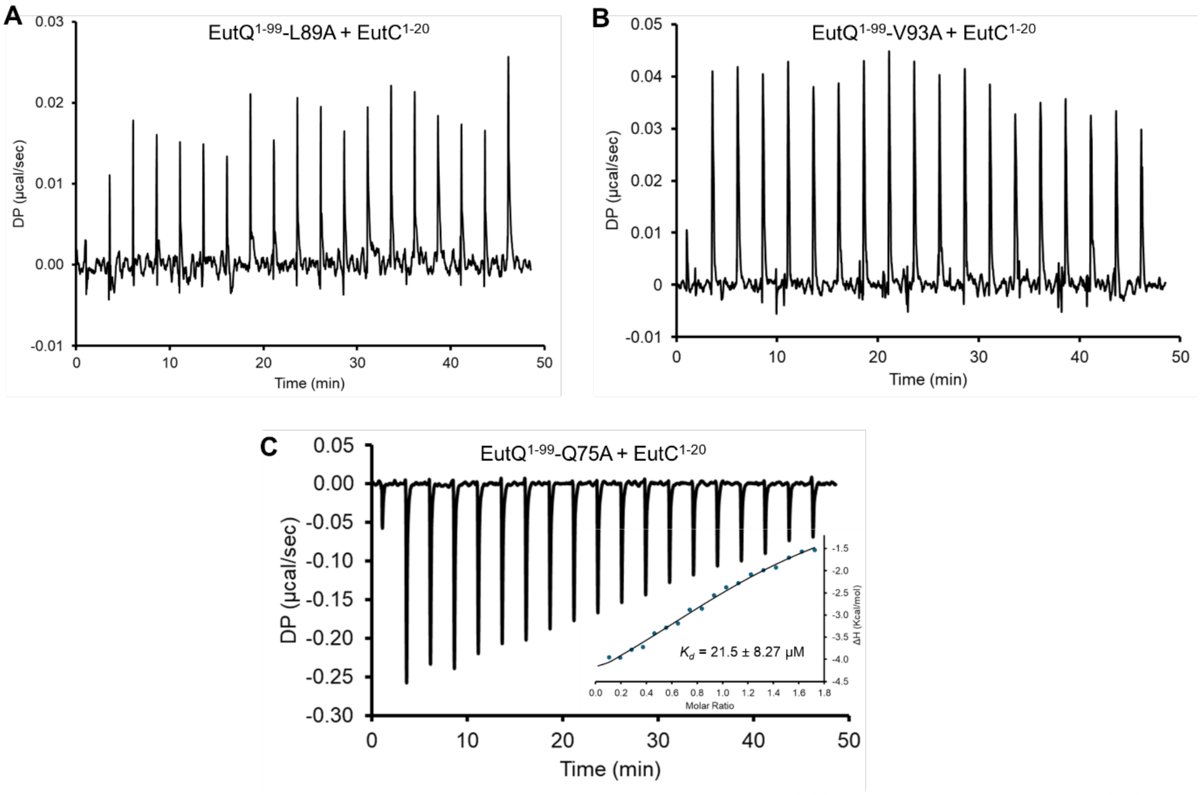

Fig. S26. **The fitted isotherms for the interaction of EutC$^{1-20}$ with various EutQ$^{1-99}$-GB1 mutants, as determined by ITC. A**, EutQ$^{1-99}$-GB1 (L89A) exhibited no binding. **B**, EutQ$^{1-99}$-GB1 (V93A) exhibited no binding. **C**, EutQ$^{1-99}$-GB1 (Q75A) demonstrated measurable binding with a stoichiometry (N) of approximately 1, a Gibbs free energy change ($\Delta G°$) of -6.39 kcal mol^{-1}, an enthalpy change ($\Delta H°$) of -5.45 ± 0.75 kcal mol^{-1}, and an entropic contribution ($T\Delta S$) of 0.94 kcal mol^{-1}. The inset summarizes the thermodynamic parameters.

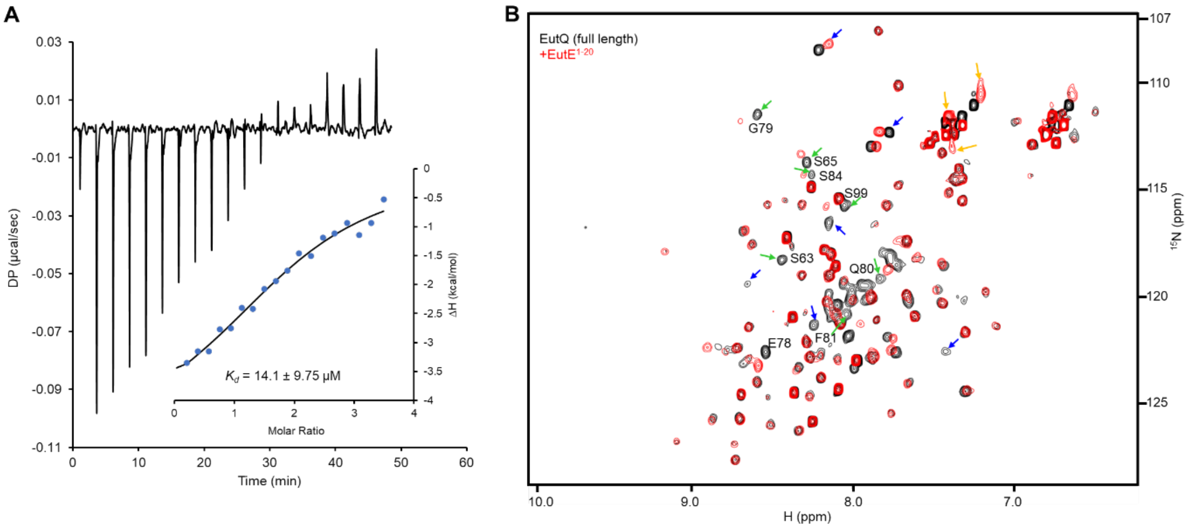

Fig. S27. **EutE$^{1\text{-}20}$ interacts with EutQ$^{1\text{-}99}$. A**, Fitted isotherm of EutE$^{1\text{-}20}$ binding with EutQ (N ~ 2 with the peptide binding to each monomeric subunit in the EutQ dimer; K_d = 14.1 µM; $\Delta G°$ = -6.62 kcal mol^{-1}; $\Delta H°$ = -4.66 kcal mol^{-1}; $T\Delta S$ = -1.96 kcal mol^{-1}). **B**, 2D ^{15}N-^{1}H HSQC spectrum (298K) of uniformly ^{15}N-labeled EutQ (black) overlaid with ^{15}N-labeled EutQ mixed with five-fold excess unlabeled EutE$^{1\text{-}20}$ (red). Assigned peaks are those derived from the EutQ N-terminal domain residues, which show chemical shift perturbations in the presence of EutE$^{1\text{-}20}$. Assigned peaks (63, 65, 78-81, 84, 99) chemical shift perturbations are highlighted with green arrows; unassigned peaks perturbations with blue arrows; Gln and Asn amide side chains with yellow arrows. Severe linewidth broadening precluded the assignments of many resonances in H3 and H4 regions.

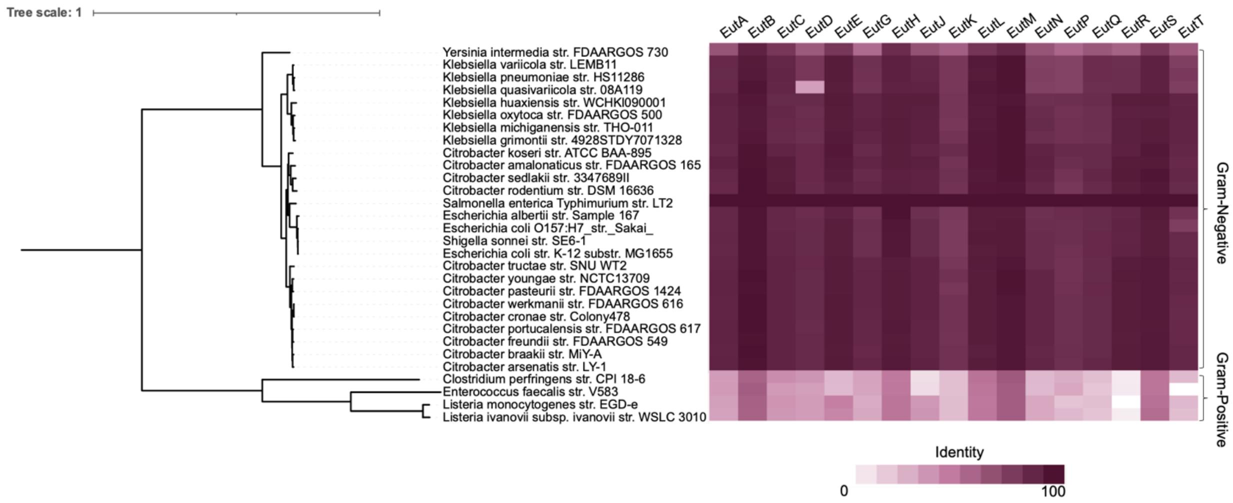

Fig. S28. Similarities among Eut proteins from different bacterial species at the amino acid level. The phylogenetic tree was made from an alignment of 30 representative isolates containing the Eut BMCs (see Supplemental File 1). The tree was rooted on the middle point of Gram-negative and Gram-positive bacteria because they are too far away. The heatmap shows the similarity of all 17 Eut protein sequences of the 30 genomes comparing to *S.* Typhimurium LT2. Eut protein sequences are highly conserved among Gram-negative bacteria, whereas the similarity of Eut proteins between *S*. Typhimurium LT2 and Gram-positive bacteria is low.

EutQ

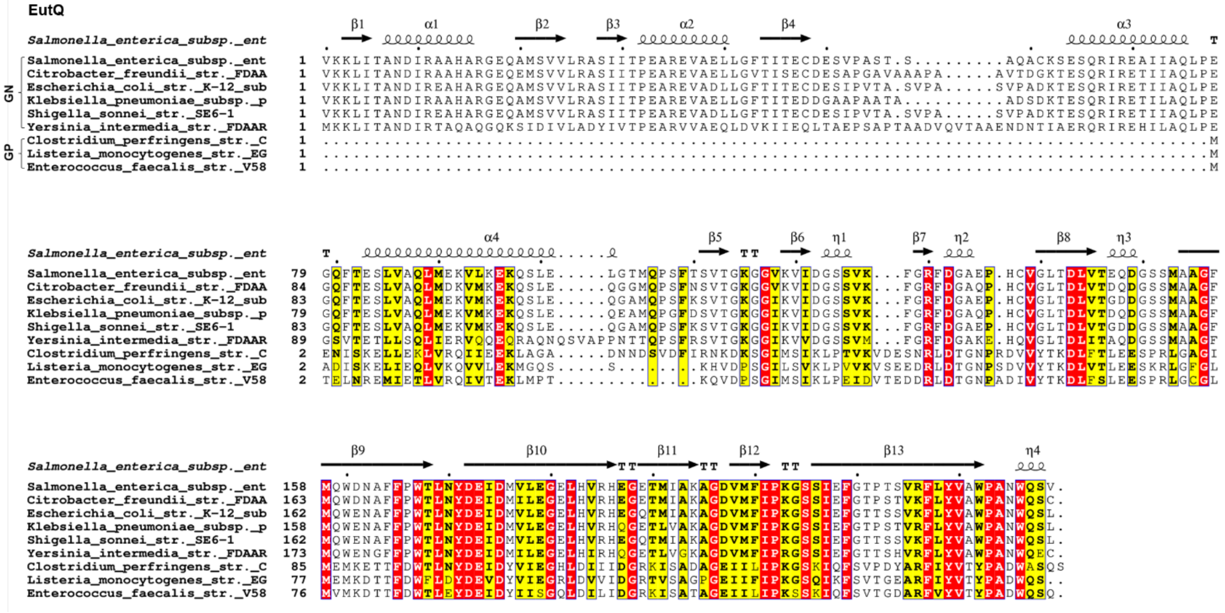
Salmonella_enterica_subsp._ent
GN
Salmonella_enterica_subsp._ent
Citrobacter_freundii_str._FDAA
Escherichia_coli_str._K-12_sub
Klebsiella_pneumoniae_subsp._p
Shigella_sonnei_str._SE6-1
Yersinia_intermedia_str._FDAAR
GP
Clostridium_perfringens_str._C
Listeria_monocytogenes_str._EG
Enterococcus_faecalis_str._V58

EutC

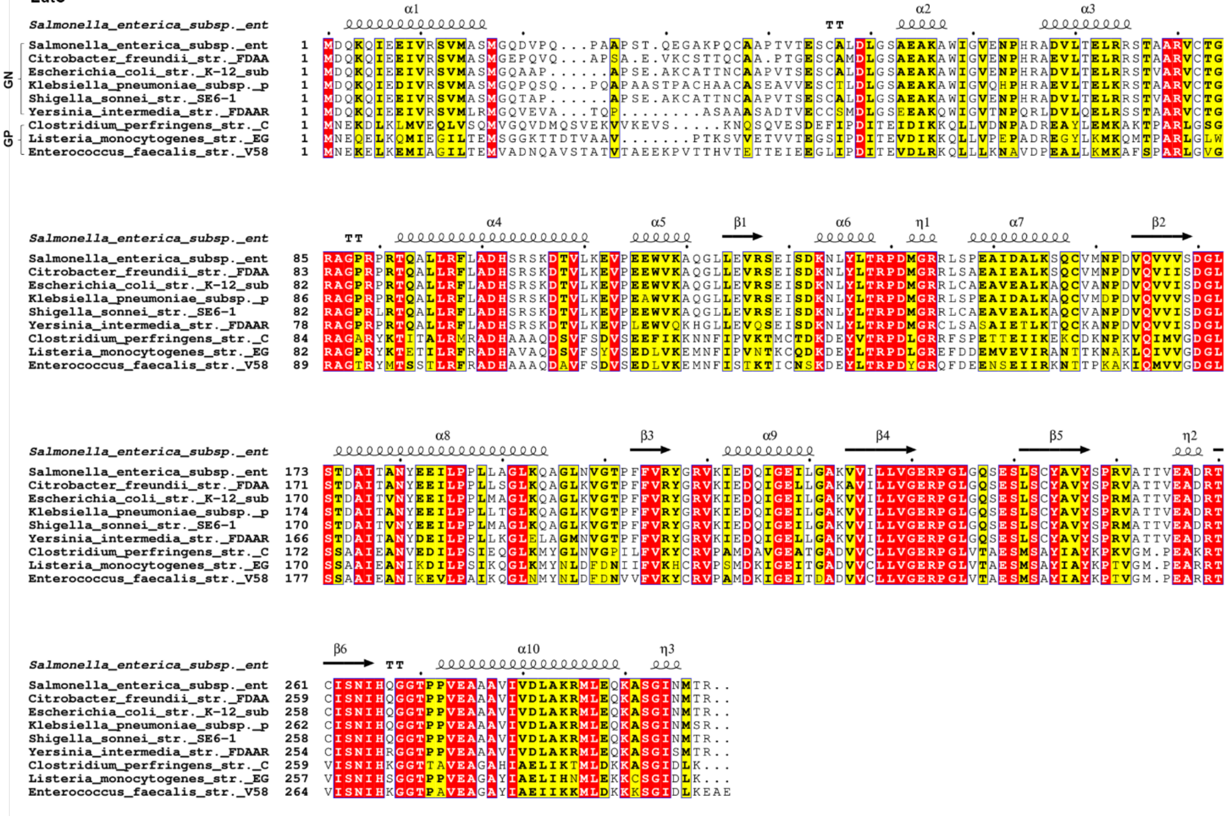
Salmonella_enterica_subsp._ent
GN
Salmonella_enterica_subsp._ent
Citrobacter_freundii_str._FDAA
Escherichia_coli_str._K-12_sub
Klebsiella_pneumoniae_subsp._p
Shigella_sonnei_str._SE6-1
Yersinia_intermedia_str._FDAAR
GP
Clostridium_perfringens_str._C
Listeria_monocytogenes_str._EG
Enterococcus_faecalis_str._V58

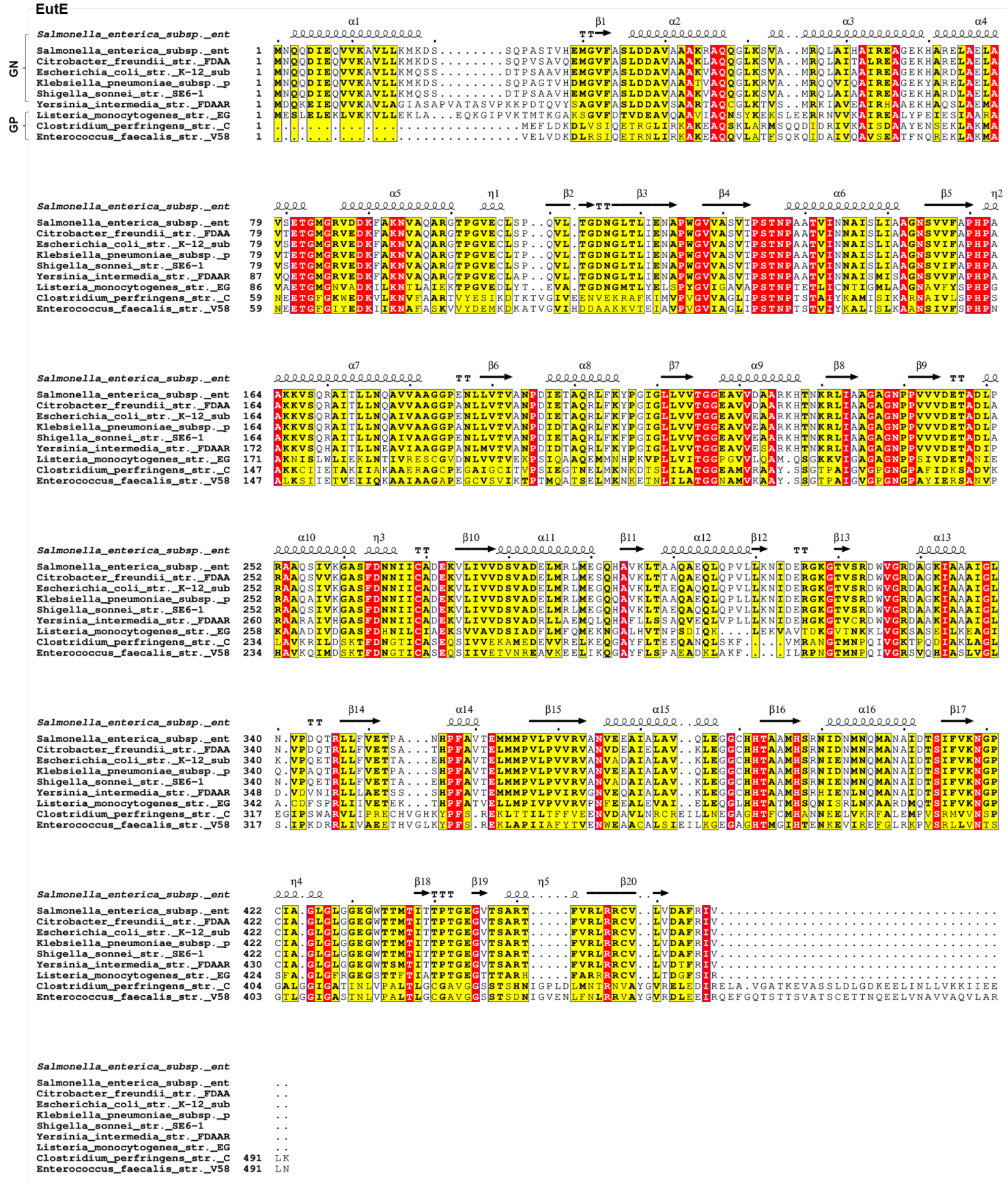

Fig. S29. Sequence alignment of EutQ, EutC and EutE from different bacterial species. Eut protein structures are highly conserved among Gram-negative (GN) bacteria, whereas the similarity of Eut proteins between S. Typhimurium LT2 and Gram-positive (GP) bacteria is low. Red boxes indicate completely conserved amino acid residues and similar residues are written with black bold characters and boxed in yellow.

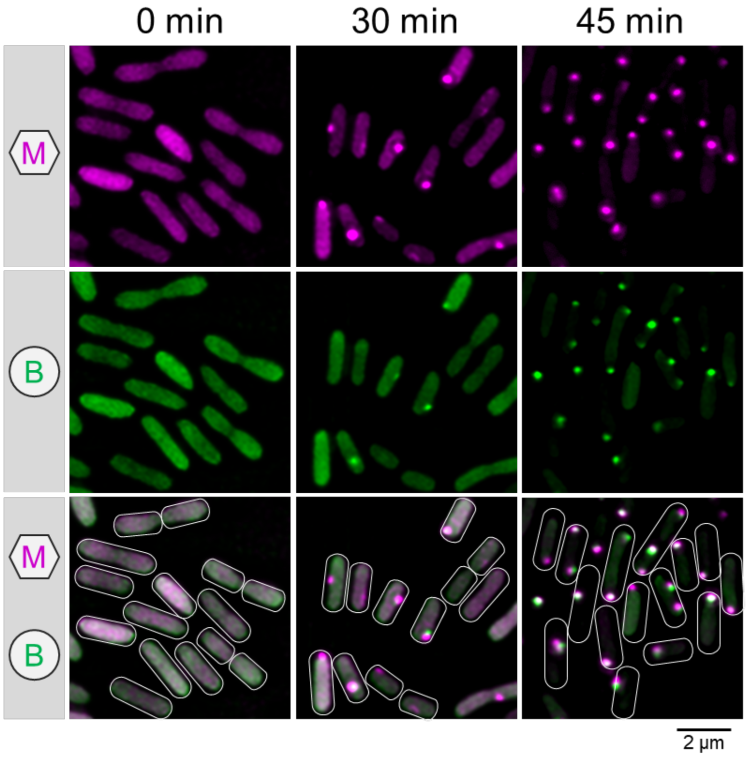

Fig. S30. Imaging the Eut BMC biogenesis in liquid media. EutM-mCherry and EutB-sfGFP were visualized in WT strain following growth in the liquid minimal medium before (0 min) and after (30 and 45 min) EA and B_{12} induction.

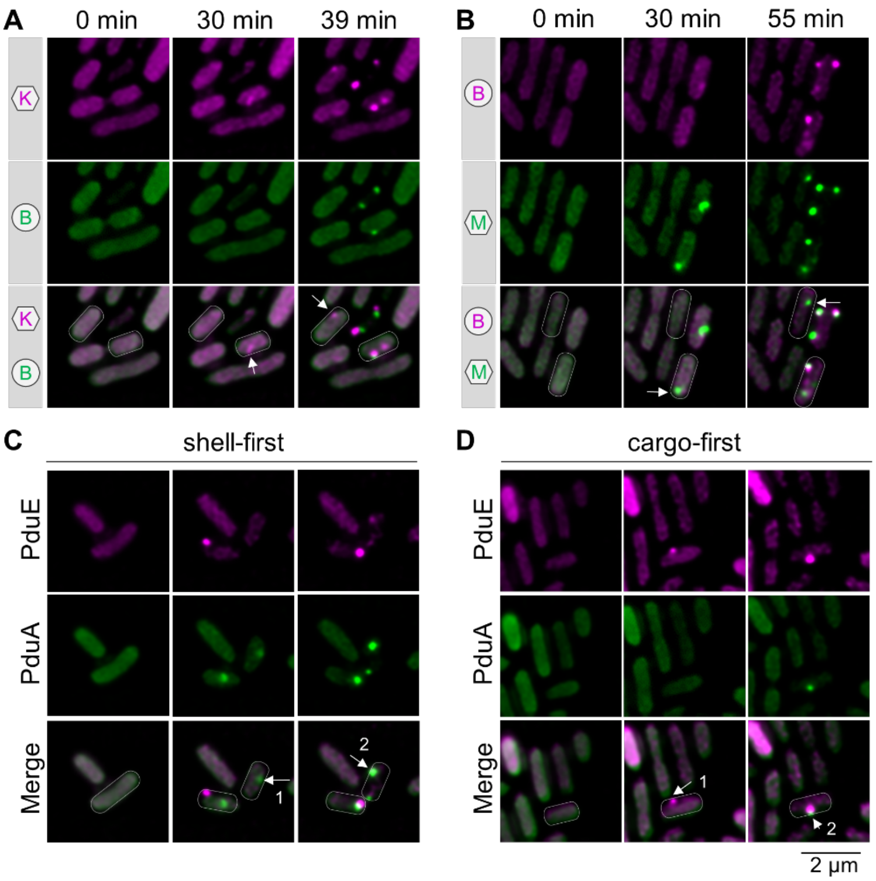

Fig. S31. Different assembly pathways of Eut BMC and Pdu BMC. A and **B**, Aggregation of the shell (EutK-mCherry or EutM-sfGFP) and cargo (EutB-sfGFP or EutB-mCherry) of Eut BMC in the WT strain following induction with EA and B_{12}. **C** and **D**, Aggregation of the shell (PduA-sfGFP) and cargo (PduE-mCherry) of Pdu BMC in the WT strain following induction with 1,2-propanediol. Number '1' represents the initial assembly of the shell or cargo; number '2' represents colocalization of the shell and cargo.

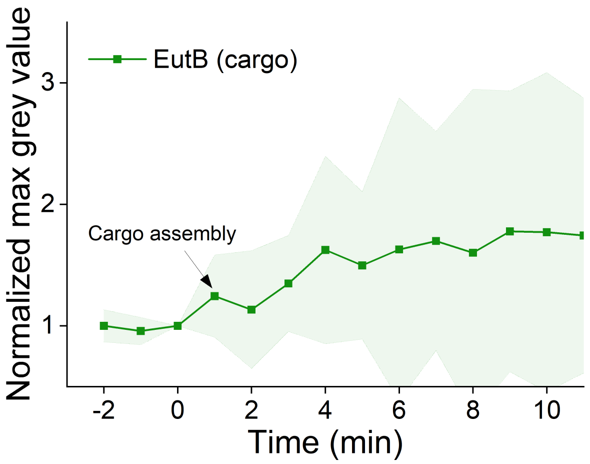

Fig. S32. Time course of the normalized max fluorescence intensity of the cargo protein EutB during Eut BMC assembly. The time point of the first cargo assembly was normalized to 1 min.

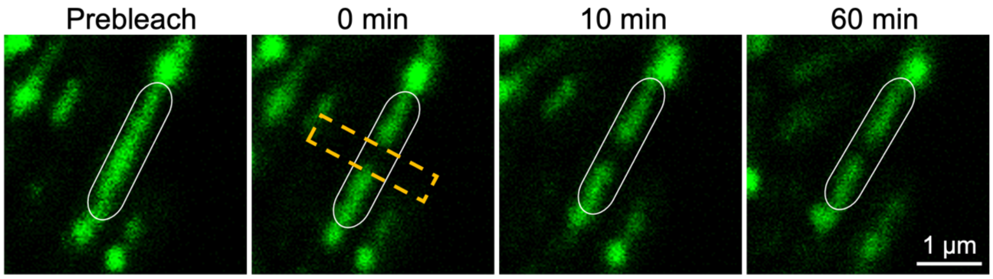

Fig. S33. Representative FRAP images of EutM-sfGFP (shell) at various time lapses. The yellow rectangular boxes indicate the bleaching area.

Table S1. Strains and plasmids used in this study. Relevant antibiotic resistances are indicated by ^{R}: Ap, ampicillin; Km, kanamycin; Cm, chloramphenicol; Gm, gentamicin; Tc, tetracycline.

Strains/Plasmids	Description	Reference/Origin
LT2	LT2, WT	*(65)*
LT2-Δ*eutS*	Δ*eutS*	This study
LT2-Δ*eutN*	Δ*eutN*	This study
LT2-Δ*eutL*	Δ*eutL*	This study
LT2-Δ*eutK*	Δ*eutK*	This study
LT2-Δ*eutM*	Δ*eutM*	This study
LT2-Δ*eutQ*	Δ*eutQ*	This study
LT2-Δ$eutQ^{9\text{-}99}$	Δ$eutQ^{9\text{-}99}$	This study
LT2-Δ$eutQ^{100\text{-}229}$	Δ$eutQ^{100\text{-}229}$	This study
LT2-Δ$eutQ^{9\text{-}41}$	Δ$eutQ^{9\text{-}41}$	This study
LT2-Δ$eutQ^{42\text{-}61}$	Δ$eutQ^{42\text{-}61}$	This study
LT2-Δ$eutQ^{62\text{-}99}$	Δ$eutQ^{62\text{-}99}$	This study
LT2-Δ$eutQ^{9\text{-}21}$	Δ$eutQ^{9\text{-}21}$	This study
LT2-Δ$eutQ^{22\text{-}41}$	Δ$eutQ^{22\text{-}41}$	This study
LT2-Δ$eutQ^{62\text{-}79}$	Δ$eutQ^{62\text{-}79}$	This study
LT2-Δ$eutQ^{80\text{-}99}$	Δ$eutQ^{80\text{-}99}$	This study
LT2-Δ$eutE^{2\text{-}21}$	Δ$eutE^{2\text{-}21}$	This study
LT2-Δ$eutC^{2\text{-}19}$	Δ$eutC^{2\text{-}19}$	This study
LT2-Δ*eutP*	Δ*eutP*	This study
LT2-Δ*eutPQ*	Δ*eutPQ*	This study
LT2-Δ*eutJ*	Δ*eutJ*	This study
LT2-Δ$eutG^{5\text{-}19}$	Δ$eutG^{5\text{-}19}$	This study
***E. coli* derivatives:**		
E. coli S17-1 λ*pir*	*pro thi hsdR recA* chromosome::RP4-2 Tc::Mu Km::Tn7/λ*pir*; Tp^R, Sm^R	
**Plasmids:**		
pEMG	Suicide plasmid; Km^R	*(66)*
pSW-2	Plasmid for m-toluate–inducible expression of the I-SceI enzyme; Gm^R	*(66)*
pBAD/*Myc*-His	Vector for dose-dependent expression of recombinant proteins; Ap^R	Invitrogen
pXG10-SF	Plasmid served as the backbone for complementation experiments; pSC101* origin of replication; $P_{LtetO\text{-}1}$ promoter; Cm^R	*(68)*
pXG10-*eutK*	Plasmid for expression of EutK; Cm^R	This study
pXG10-*eutL*	Plasmid for expression of EutL; Cm^R	This study
pXG10-*eutQ*	Plasmid for expression of EutQ; Cm^R	This study

pBAD-*MB*	*eutM::mCherry-eutB::sfGFP* cloned into pBAD/*Myc*-His at NcoI and HindIII sites; Ap^R	This study
pBAD-*KB*	*eutK::mCherry-eutB::sfGFP* cloned into pBAD/*Myc*-His at NcoI and HindIII sites; Ap^R	This study
pBAD-*LB*	*eutL::mCherry-eutB::sfGFP* cloned into pBAD/*Myc*-His at NcoI and HindIII sites; Ap^R	This study
pBAD-*EB*	*eutE::mCherry-eutB::sfGFP* cloned into pBAD/*Myc*-His at NcoI and HindIII sites; Ap^R	This study
pBAD-*GB*	*eutG::mCherry-eutB::sfGFP* cloned into pBAD/*Myc*-His at NcoI and HindIII sites; Ap^R	This study
pBAD-*PB*	*eutP::mCherry-eutB::sfGFP* cloned into pBAD/*Myc*-His at NcoI and HindIII sites; Ap^R	This study
pBAD-*JB*	*eutJ::mCherry-eutB::sfGFP* cloned into pBAD/*Myc*-His at NcoI and HindIII sites; Ap^R	This study
pBAD-*RB*	*eutR::mCherry-eutB::sfGFP* cloned into pBAD/*Myc*-His at NcoI and HindIII sites; Ap^R	This study
pBAD-*QB*	*eutQ::mCherry-eutB::sfGFP* cloned into pBAD/*Myc*-His at NcoI and HindIII sites; Ap^R	This study
pBAD-$Q^{\Delta 9\text{-}99}B$	$eutQ^{\Delta 9\text{-}99}$*::mCherry-eutB::sfGFP* cloned into pBAD/*Myc*-His at NcoI and HindIII sites; Ap^R	This study
pBAD-$Q^{\Delta 9\text{-}41}B$	$eutQ^{\Delta 9\text{-}41}$*::mCherry-eutB::sfGFP* cloned into pBAD/*Myc*-His at NcoI and HindIII sites; Ap^R	This study
pBAD-$Q^{\Delta 42\text{-}61}B$	$eutQ^{\Delta 42\text{-}61}$*::mCherry-eutB::sfGFP* cloned into pBAD/*Myc*-His at NcoI and HindIII sites; Ap^R	This study
pBAD-$Q^{\Delta 62\text{-}99}B$	$eutQ^{\Delta 62\text{-}99}$*::mCherry-eutB::sfGFP* cloned into pBAD/*Myc*-His at NcoI and HindIII sites; Ap^R	This study
pBAD-$E^{\Delta 2\text{-}21}B$	$eutE^{\Delta 2\text{-}21}$*::mCherry-eutB::sfGFP* cloned into pBAD/*Myc*-His at NcoI and HindIII sites; Ap^R	This study
pBAD-$C^{\Delta 2\text{-}19}B$	$eutC^{\Delta 2\text{-}19}$*::mCherry-eutB::sfGFP* cloned into pBAD/*Myc*-His at NcoI and HindIII sites; Ap^R	This study
pBAD-*BM*	*eutB::mCherry-eutM::sfGFP* cloned into pBAD/*Myc*-His at NcoI and HindIII sites; Ap^R	This study
pBAD-*KM*	*eutK::mCherry-eutM::sfGFP* cloned into pBAD/*Myc*-His at NcoI and HindIII sites; Ap^R	This study
pBAD-*LM*	*eutL::mCherry-eutM::sfGFP* cloned into pBAD/*Myc*-His at NcoI and HindIII sites; Ap^R	This study
pBAD-*QM*	*eutQ::mCherry-eutM::sfGFP* cloned into pBAD/*Myc*-His at NcoI and HindIII sites; Ap^R	This study
pBAD-$Q^{\Delta 62\text{-}99}M$	$eutQ^{\Delta 62\text{-}99}$*::mCherry-eutM::sfGFP* cloned into pBAD/*Myc*-His at NcoI and HindIII sites; Ap^R	This study
pBAD-*MaeB-EutM*	*maeB::mCherry-eutM::sfGFP* cloned into pBAD/*Myc*-His at NcoI and HindIII sites; Ap^R	This study
pBAD-*TalA-EutM*	*talA::mCherry-eutM::sfGFP* cloned into pBAD/*Myc*-His at NcoI and HindIII sites; Ap^R	This study
pBAD-*ypfG-EutM*	*ypfG::mCherry-eutM::sfGFP* cloned into pBAD/*Myc*-His at NcoI and HindIII sites; Ap^R	This study
pBAD-*AmiA-EutM*	*amiA::mCherry-eutM::sfGFP* cloned into pBAD/*Myc*-His at NcoI and HindIII sites; Ap^R	This study
pBAD-eutBC-sfGFP	*eutBC::sfGFP* cloned into pBAD/Myc-His at NcoI and HindIII sites; Ap^R	This study

pBAD-eutQ$^{1-99}$-sfGFP	*EutQ$^{1-99}$::sfGFP* cloned into pBAD/Myc-His at NcoI and HindIII sites; Ap^R	This study
pBAD-eutC$^{1-19}$-sfGFP	*EutC$^{1-19}$::sfGFP* cloned into pBAD/Myc-His at NcoI and HindIII sites; Ap^R	This study
pBAD-eutG$^{1-19}$-sfGFP	*EutG$^{1-19}$::sfGFP* cloned into pBAD/Myc-His at NcoI and HindIII sites; Ap^R	This study
pET14b-EutQ	*eutQ* cloned into pET14b backbone; Ap^R	This study
pETM11-EutQ$^{1-99}$-GB1	*eutQ$^{1-99}$-GB1* cloned into pETM11 backbone; Km^R	This study
pETM11-EutQ$^{100-229}$	*eutQ$^{100-229}$* cloned into pETM11 backbone; Km^R	This study
pETM11-GB1	*GB1* cloned into pETM11 backbone; Km^R	This study
pETM11-EutQ$^{1-99}$-V93A-GB1	*eutQ$^{1-99}$-V93A* cloned into pETM11-EutQ$^{1-99}$-GB1 backbone; Km^R	This study
pETM11-EutQ$^{1-99}$-L89A-GB1	*eutQ$^{1-99}$-L89A* cloned into pETM11-EutQ$^{1-99}$-GB1 backbone; Km^R	This study
pETM11-EutQ$^{1-99}$-R67K-GB1	*eutQ$^{1-99}$-R67K* cloned into pETM11-EutQ$^{1-99}$-GB1 backbone; Km^R	This study
pETM11-EutQ$^{1-99}$-Q75A-GB1	*eutQ$^{1-99}$-Q75A* cloned into pETM11-EutQ$^{1-99}$-GB1 backbone; Km^R	This study
pETM11-EutQ$^{1-99}$-E64A-GB1	*eutQ$^{1-99}$-E64A* cloned into pETM11-EutQ$^{1-99}$-GB1 backbone; Km^R	This study

Table S2. ssDNA Oligonucleotides used in this study.

Primers	Sequence (5'→3')
eutS-del-F1	AGGGATAACAGGGTAATCTGAATTCAGGACTACATAAAGCACCAG
eutS-del-R1	CCGCCACCAATTAACTTTTGTCATTGCTCACCGTCTCCAC
eutS-del-F2	GTGGAGACGGTGAGCAATGACAAAAGTTAATTGGTGGCG
eutS-del-R2	CCTGCAGGTCGACTCTAGAGGATCGTAATCGTGAAGCCCAGTAG
eutM-del-F1	AGGGATAACAGGGTAATCTGAATTCGGATATCGCTCTCGCCA
eutM-del-R1	CTTATCCGGCCTACCATCACATCGTGTTTTCCTCTCATTAAA
eutM-del-F2	TTTAATGAGAGGAAAACACGATGTGATGGTAGGCCGGATAAGA
eutM-del-R2	CCTGCAGGTCGACTCTAGAGGATCCTGCGTCATCCAGGGAG
eutN-del-F1	AGGGATAACAGGGTAATCTGAATTCGCTAATTCAGGGGCTTGC
eutN-del-R1	CATGATGTTCAATCCTATTTATGCATGGATTGCCCGTTGGGTC
eutN-del-F2	GACCCAACGGGCAATCCATGCATAAATAGGATTGAACATCATG
eutN-del-R2	CCTGCAGGTCGACTCTAGAGGATCAGAGTGATTGCCCGCTGA
eutL-del-F1	AGGGATAACAGGGTAATCTGAATTGTGCGTTCGGAGATCAGC
eutL-del-R1	CAGCCTCCGTTACGCACGCATGATGTCTCCTTAACGGG
eutL-del-F2	CCCGTTAAGGAGACATCATGCGTGCGTAACGGAGGCTG
eutL-del-R2	CCTGCAGGTCGACTCTAGAGGATCCGTCTTCGGGTAACGCTTC
eutK-del-F1	AGGGATAACAGGGTAATCTGAATTCCTATATTGCCGCTGACGAAG
eutK-del-R1	CCCGGCCCTCCGACAGATTACATTGGCAGCCTCCGTTA
eutK-del-F2	TAACGGAGGCTGCCAATGTAATCTGTCGGAGGGCCGGG
eutK-del-R2	CCTGCAGGTCGACTCTAGAGGATCCATATGCAGCACCCTTTCC
eutP-del-F1	AGGGATAACAGGGTAATCTGAATTGCACAAATCGAGTGATACGC
eutP-del-R1	CTCTTCCTCCTGTTCACTTTACATTACCGCCACCAATTAACTTTTTG
eutP-del-F2	CAAAAAGTTAATTGGTGGCGGTAATGTAAAGTGAACAGGAGGAAGAG
eutP-del-R2	CCTGCAGGTCGACTCTAGAGGATCGAACATCACATCTCCGGC
eutJ-del-F1	AGGGATAACAGGGTAATCTGAATTGTGCCGACGAAAAAGTGC
eutJ-del-R1	CCCTCCGCTTTCGCTCTCATGAGTCATCCCTTATACAATG
eutJ-del-F2	CATTGTATAAGGGATGACTCATGAGAGCGAAAGCGGAGGG
eutJ-del-R2	CCTGCAGGTCGACTCTAGAGGATCCAAGAATCGCCACGTCC
eutC2-19-del-F1	AGGGATAACAGGGTAATCTGAATTCAAGGGTCTGCGCTCTCC
eutC2-19-del-R1	CGGGCTGCGGTACGTCCATGGTGTTATCCCCGCG
eutC2-19-del-F2	CGCGGGGATAACACCATGGACGTACCGCAGCCC
eutC2-19-del-R2	CCTGCAGGTCGACTCTAGAGGATCGGCGTGCCGACGTTCAG
eutE2-21-del-F1	AGGGATAACAGGGTAATCTGAATT GCAAAGCCGCAACCGATG
eutE2-21-del-R1	GAACAGTACTGGCCGGCTGCATGATGTTCAATCCTATTTATGG
eutE2-21-del-F2	CCATAAATAGGATTGAACATCATGCAGCCGGCCAGTACTGTTC
eutE2-21-del-R2	CCTGCAGGTCGACTCTAGAGGATC GTATGTTTACGCGCCGCG
eutG5-19-del-F1	AGGGATAACAGGGTAATCTGAATTGTCGATTTGGGCACCTGC
eutG5-19-del-R1	GCTGAACGTTTTCACCCGTTCAGCTTGCATAGAGTCCCTC
eutG5-19-del-F2	GAGGGACTCTATGCAAGCTGAACGGGTGAAAACGTTCAGC
eutG5-19-del-R	CCTGCAGGTCGACTCTAGAGGATCCCATTCCCGCCATACAGGAG
eutQ-del-F1	AGGGATAACAGGGTAATCTGAATTGCGCTATTACAGACGGTCAG
eutQ-del-R1	CGGATTGCCAGTTTGCAGGCTAGTTAGCTGTGATAAGTTTTTTCACC
eutQ-del-F2	GGTGAAAAAACTTATCACAGCTAACTAGCCTGCAAACTGGCAATCCG
eutQ-del-R2	CCTGCAGGTCGACTCTAGAGGATCCTCATCCTCGTTCAGTCC
eutPQ-del-R1	CGGATTGCCAGTTTGCAGGCTTACATTACCGCCACCAATTAACTTTTTG
eutPQ-del-F2	CAAAAAGTTAATTGGTGGCGGTAATGTAAGCCTGCAAACTGGCAATCCG

eutQ-del-9-99-R1	CATCGTCCCCAGTTCCAGGTTAGCTGTGATAAGTTTTTCACC
eutQ-del-9-99-F2	GGTGAAAAAACTTATCACAGCTAACCTGGAACTGGGGACGATG
eutQ-del-100-229-R1	CGGATTGCCAGTTTGCAGGCTACGACTGCTTTTCCTTCAGC
eutQ-del-100-229-F2	GCTGAAGGAAAAGCAGTCGTAGCCTGCAAACTGGCAATCCG
eutQ-del-9-21-R1	GCGCAGCACTACCGAGTTAGCTGTGATAAGTTTTTCACC
eutQ-del-9-21-F2	GGTGAAAAAACTTATCACAGCTAACTCGGTAGTGCTGCGC
eutQ-del-9-41-R1	CATTCGGTAATCGTGAAGCCGTTAGCTGTGATAAGTTTTTCACC
eutQ-del-9-41-F2	GGTGAAAAAACTTATCACAGCTAAC GGCTTCACGATTACCGAATG
eutQ-del-22-41-R1	CATTCGGTAATCGTGAAGCCCATCGCCTGTTCGCCGCGGG
eutQ-del-22-41-F2	CGGCGAACAGGCGATGGGCTTCACGATTACCGAATG
eutQ-del-42-61-R1	TGCGCTGGCTTTCGCTTTTCAGTAGTTCCGCCACTTCG
eutQ-del-42-61-F2	CGAAGTGGCGGAACTACTG AAAAGCGAAAGCCAGCGCA
eutQ-del-62-79-R1	CACCAGGCTTTCAGTAAACTGACACGCCTGCGCCGATG
eutQ-del-62-79-F2	TCGGCGCAGGCGTGT CAGTTTACTGAAAGCCTGGTG
eutQ-del-80-99-R1	CATCGTCCCCAGTTCCAGCCCTTCCGGGAGCTG
eutQ-del-80-99-F2	CAGCTCCCGGAAGGG CTGGAACTGGGGACGATG
eutQ-del-62-99-R1	CATCGTCCCCAGTTCCAGACACGCCTGCGCCGATG
eutQ-del-62-99-F2	CATCGGCGCAGGCGTGT CTGGAACTGGGGACGATG
eutS-up	GCACAAATCGAGTGATACGC
eutS-down	CGCGTGGACATAAATCAGCG
eutM-up	GATGATTTTCCCGTCGCTGG
eutM-down	TACTGTCGATAGCGACGGC
eutN-up	CATTGGCGAGTTGGTCTCC
eutN-down	GAATGGCATGAATGGCAAGC
eutL-up	TCGTCATCCTGCTGGTAGG
eutL-down	CTTTGCGACTAATCACACGGC
eutK-up	GAAGCGACATTCGGCATTG
eutK-down	GATCGTAGATCTGCTGCCAG
eutP-up	GGCGATTTAGCGATGAAGG
eutP-down	CAGTAAACTGCCCTTCCGG
eutJ-up	CATCGACACCAGCATTTTCG
eutJ-down	TCATTCCCGCCTGATGCA
EutG5-19-up	GCCACATTGAAGGGCAAG
EutG5-19-down	CAGACATCGGTGATGCACG
eutE2-21-up	ATCGATGCCCAGGGCAAC
eutE2-21-down	GCGGAGATAAACACTCGACG
eutC2-19-up	GCTGTAACTACATCATGGGGA
eutC2-19-down	CCAGGAAGCGCAACAGC
eutQ-up	GGAACCCGTAAACGACATATC
eutQ-down	GTTAAGCCATGTACCGGC
pBAD-eutMB-F1	GGGCTAACAGGAGGAATTAACCATGGAAGCATTAGG
pBAD-eutMB-R1	CGTCCTCGAAGTTCATCACGCGCTCCCACTTGAAG
pBAD-eutMB-F2	CTTCAAGTGGGAGCGCGTGATGAACTTCGAGGACG
pBAD-eutMB-R2	ATTCCTAATGCTTCCATGGTTAATTCCTCCTGTTAGCCCCTACTTG
pBAD-eutMB-F3	AACCATGGAAGCATTAGGAATGATTGAAACCCGGGGCCTGGTTGCGCTGATTGAGG CCT
pBAD-eutMB-R3	TTTGCTCATGAATTCGCCAGAACCAGCAGCGGAGCCAGCGGATCCAATGTTGCTGT CGCC
pBAD-eutMB-F4	CTGGCGAATTCATGAGCAAAG
pBAD-eutMB-R4	CTGAGATGAGTTTTTGTTCTACGTA
pBAD-mCherry-eutB/M-sfGFP-F1	CTGGCTCCGCTGCTGGTTCTGGCGAATTCGTGAGCAAGGGCGAGGAG
pBAD-mCherry-eutB/M-sfGFP-R1	GAGATGAGTTTTTGTTCTACGTATTATTTGTAGAGCTCATCCATGCCA
pBAD-eutE-F1	GGGCTAACAGGAGGAATTAACCATGAATCAACAGGATATTGAACAGG
pBAD-eutE-R1	AGAACCAGCAGCGGAGCCAGCGGATCCTACAATGCGAAACGCATCCA
pBAD-eutE-KO2-21-F1	GGGCTAACAGGAGGAATTAACCATGCAGCCGGCCAGTACTGTTC

pBAD-eutP-F1	GGGCTAACAGGAGGAATTAACCATGAAACGTATTGCTTTTGTCG
pBAD-eutP-R1	AGAACCAGCAGCGGAGCCAGCGGATCCGCTGTGATAAGTTTTTCACCTG
pBAD-eutQ-F1	GGGCTAACAGGAGGAATTAACCATGAAAAAACTTATCACAGCTAAC
pBAD-eutQ-R1	AGAACCAGCAGCGGAGCCAGCGGATCCTACGGATTGCCAGTTTGC
pBAD-eutQ1-99-R1	AGCAGCGGAGCCAGCGGATCCCGACTGCTTTTCCTTCAGCAC
pBAD-eutJ-F1	GGGCTAACAGGAGGAATTAACCATGGCGCACGACGAACA
pBAD-eutJ-R1	AGAACCAGCAGCGGAGCCAGCGGATCCGCTTGCATAGAGTCCCTCC
pBAD-eutR-F1	GGGCTAACAGGAGGAATTAACCATGAAAAAGACCCGTACAGC
pBAD-eutR-R1	AGAACCAGCAGCGGAGCCAGCGGATCCAGCCCATTGCCGCATCC
pBAD-eutK-F1	GGGCTAACAGGAGGAATTAACCATGATCAATGCCCTGGGA
pBAD-eutK-R1	AGAACCAGCAGCGGAGCCAGCGGATCCATTTTTGATGCGATAGCGACTA
pBAD-eutL-F1	GGGCTAACAGGAGGAATTAACCATGCCTGCATTAGATTTAATTCG
pBAD-eutL-R1	AGAACCAGCAGCGGAGCCAGCGGATCCCGCACGCTGGACAGG
pBAD-eutG-F1	GGGCTAACAGGAGGAATTAACCATGCAAGCTGAACTACAGAC
pBAD-eutG-R1	AGAACCAGCAGCGGAGCCAGCGGATCCCCCGGCAGCCGCGTAC
pBAD-eutG1-19-F1	GGCGCTGTTTCAGGCATTCGACACCCTGAATCTGCAAGGATCCGCTGGCTCCGC
pBAD-eutG1-19-R1	CGAATGCCTGAAACAGCGCCGTCTGTAGTTCAGCTTGCATGGTTAATTCCTCCTGTT AGC
pBAD-eutC-KO-2-19-F1	GGGCTAACAGGAGGAATTAACCATGGACGTACCGCAGCCCGCC
pBAD-eutC-R1	AGAACCAGCAGCGGAGCCAGCGGATCCACGGGTCATGTTGATGCCG
pBAD-eutC1-19-F1	AGAAATTGTACGTAGCGTGATGGCGTCAATGGGACAGGGATCCGCTGGCTCCGC
pBAD-eutC1-19-R1	TCACGCTACGTACAATTTCTTCAATCTGTTTTTGATCCATGGTTAATTCCTCCTGTTA GC
pBAD-BC-sfGFP-F1	GGGCTAACAGGAGGAATTAACCATGAAACTAAAGACCACATTGTTCGG
pBAD-BC-sfGFP-R1	GGAGCCAGCGGATCCACGGGTCATGTTGATGC
pBAD-BC-sfGFP-F2	CCCGTGGATCCGCTGGCTCCG
pBAD-BC-sfGFP-R2	GAGATGAGTTTTTGTTCTACGTAAGCTT
pBAD-maeB-F1	GGGCTAACAGGAGGAATTAACCATGGATGAGCAGTTAAAACAAAGC
pBAD-maeB-R1	AGAACCAGCAGCGGAGCCAGCGGATCCAAGCGGCGTGGTTTGCGC
pBAD-talA-F1	GGGCTAACAGGAGGAATTAACCaTGAACCAGCTAGACGGCAT
pBAD-talA-R1	AGAACCAGCAGCGGAGCCAGCGGATCCTAGTTTGGCGGCAAGCAGG
pBAD-ypfG-F1	GGGCTAACAGGAGGAATTAACCATGCGCTACCGCGTTATTCTTTTTG
pBAD-ypfG-R1	AGAACCAGCAGCGGAGCCAGCGGATCCACGCGTGATCCACAGCGTAG
pBAD-amiA-F1	GGGCTAACAGGAGGAATTAACCATGAGCACTTTTAAACTCCTAAAAACTCTCACAT C
pBAD-amiA-R1	AGAACCAGCAGCGGAGCCAGCGGATCCCCGTTTCTTCGTGTGTGCTTTCTG
pXG10-eutL-F	GTGATAGAGATACTGAGCACATGCATTCCGGCATCAACATGACCCG
pXG10-eutL-R	CTTTCGTTTTATTTGATGCCTCTAGATTACGCACGCTGGACAGGGTTA
pXG10-eutQ-F	GTGATAGAGATACTGAGCACATGCATTGGCGGCTCTCAGTGAACA
pXG10-eutQ-R	CTTTCGTTTTATTTGATGCCTCTAGATCATACGGATTGCCAGTTTGC
pXG10-eutK-F	GTGATAGAGATACTGAGCACATGCATAACCCTGTCCAGCGTGC
pXG10-eutK-R	CTTTCGTTTTATTTGATGCCTCTAGATTAATTTTTGATGCGATAGCGACTAC
pXG10-eutC-F	GTGATAGAGATACTGAGCACATGCATTCACTGTTCTTCTGATGACG
pXG10-eutC-R	CTTTCGTTTTATTTGATGCCTCTAGATTAACGGGTCATGTTGATGC
pET-22b-EutQ-His-F	CTTTAAGAAGGAGATATACATATGGTGAAAAAACTTATCACAGCTAA
pET-22b-EutQ-His-R	GTGGTGGTGGTGCTCGAGTCATACGGATTGCCAGTTTGC
pET-22b-EutQ-Strep-R	GTGGTGCTCGAGTTATTTTTCAAACTGCGGATGGCTCCATCATACGGATTGCCAGTT TG
pET-22b-EutM-His-F	CTTTAAGAAGGAGATATACATATGATGGAAGCATTAGGAATGATTGA
pET-22b-EutM-His-R	GTGGTGGTGGTGCTCGAGTCAAATGTTGCTGTCGCCTTT
pET-22b-EutL-Strep-F	CTTTAAGAAGGAGATATACATATGATGCCTGCATTAGATTTAATTCG
pET-22b-EutL-Strep-R	TGGTGGTGCTCGAGTTATTTTTCAAACTGCGGATGGCTCCACGCACGCTGGACAGG GTT
pET-22b-T7-up	TAATACGACTCACTATAGGG
pET-22b-T7-down	TGCTAGTTATTGCTCAGCGG
EutQ_For	TTTTCAGGGCAAGAGGCAGGTGAAAAAACTTATCACAGC
EutQ_Rev	AGCCGGATCCTCATACGGATTGCCAGTTTGCAGG

pET14b_EutQ_For	ATCCGTATGAGGATCCGGCTGCTAACAAAGCC
pET14b_EutQ_Rev	CCTGCCTCTTGCCCTGAAAATACAGGTTTTCCATATG
EutQ_C_Term_For	TTTTCAGGGCCTGGAACTGGGGACGATGCAG
EutQ_C_Term_Rev	CGGATCCCTATCATACGGATTGCCAGTTTGCAGG
pETM11_EutQ_C_Term_For	ATCCGTATGATAGGGATCCGAATTCGAGCTCCGTC
pETM11_EutQ_C_Term_Rev	CCAGTTCCAGGCCCTGAAAATAAAGATTCTCAGTAGTGGGGATG
EutQ_N_Term_For	ATATACCATGGTGAAAAAACTTATCACAGCTAACGATATTCGTGCGG
EutQ_N_Term_Rev	TCAGGGCTCACGACTGCTTTTCCTTCAGCACTTTTTC
GB1_His_Tev_For	AAAGCAGTCGTGAGCCCTGAAAATACAGGTTTTCCATCACCATCACCATCACCCC ATGAG
GB1_His_Tev_Rev	ATCCCTATTATTCGGTCACGGTAAAGGTTTTGG
pETM11_EutQ_N_Term_For	CGTGACCGAATAATAGGGATCCGAATTCGAGCTCC
pETM11_EutQ_N_Term_Rev	GTTTTTTCACCATGGTATATCTCCTTCTTAAAGTTAAACAAAATTATTTCTAGAGGG
EutQ_vector_For	TGAGCAATAACTAGCATAACCCCTTGG
EutQ_mutant_Rev	TTATTGCTCAGCGGTGGCAGC
L89A_For	GCGATGGAAAAAGTGCTGAAGGAAAAGC
L89A_vect_Rev	TTTCCATCGCCTGCGCCACCAGGCTTTC
V93A_For	GGAAAAAGCGCTGAAGGAAAAGCAGTCG
V93A_vect_Rev	CGCTTTTTCCATCAACTGCGCCAC
Q75A_For	GCGCTCCCGGAAGGGCAGTTTAC
Q75A_vector_Rev	CCGGGAGCGCCGCGATGATGGCTTCC
R67K_For	AAAATTCGGGAAGCCATCATCG
R67K_vector_Rev	CCCGAATTTTCTGGCTTTCGCTTTTACACGCCTG

Table S3. Additional imaging and processing parameters of light microscopy

Microscope component	Zeiss Elyra	LSM 780
Light source	Zeiss Laser module for HR systems 230V (000000-2269-457)<br>Zeiss Laser HR Diode 488 nm (000000-2266-075) 500mW – set to 500 mW and 5-20%<br>Zeiss Laser HR DPSS 561 nm (000000-1762-586) 500mW – set to 500 mW and 10-30%	Argon-Multiline Laser LGK7812 ML5<br>Wavelengths: 488 nm, 1.5%
Excitation/ Emission optics	Laser Blocking Filter: 405/488/561/642<br>Excitation and emission filters manufacturer and wavelength characteristics<br>Grating period (488 nm): 27.5 µm<br>Grating period (561 nm): 32 µm<br>Grating: 13 Phases<br>Filter module DuoLink SR QUAD (000000-2290-250) with secondary beam splitter (SBS) and emission filters (EF):<br>488 nm filter: SBS LP 560 with EF BP420-480/BP495-550 & EF BP570-620 & LP655<br>561 nm filter: SBS BP490-560/LP640 with EF BP420-480/BP570-630/LP740 & EF BP495-550/LP655	Pinhole: 1.3 AU/66 µm<br>Averaging: line 4<br><br>Beam spitter: MBS 488<br>Emission wavelength ranges:<br>488: 499-561 nm<br>Pixel dwell time: 1.58 µs<br>Channel Gain: 848<br>Digital Gain: 0.7
Objective lens	Zeiss Plan Apochromat 63×/1.40 Oil DIC f/ELYRA lens (420782-9900-799)	Zeiss Plan Apochromat 63×/1.40 Oil lens (420782-9900-000)
Detector	PCO.edge 4.2 CLHS, adjusted<br>Camera pco.edge sCMOS (Version 4.2 CL HS)<br>(D)Exposure/dwell time<br>1280 × 1280 pixels<br>16 bit	Photomultiplier tube<br>16 bit<br>512 × 512 pixels<br>Scaling X/Y: 0.026 µm
Special settings	Post-processing<br>SIM^2 Weak live/fixed filtering of the 488 and 561 channels, for live/fixed samples, respectively<br>Processing/Output sampling: 2×<br>Fast fit Median filter<br>Scaled to raw for time series images only	FRAP imaging:<br>Time interval: 60 seconds<br>Iterations: 20<br>Bleaching wavelength: 488 nm<br>Bleaching laser intensity: 100%

Movie S1. Dynamics of Eut BMC assemblies in *S.* Typhimurium WT expressing EutB-sfGFP. Cells were grown in the minimal medium in the presence of EA and B_{12}. Time-lapse was conducted with a time interval of 1.13 seconds for 45 seconds.

Movie S2. Dynamics of Eut BMC assemblies in *S.* Typhimurium Δ*eutK* expressing EutB-sfGFP. Cells were grown in the minimal medium in the presence of EA and B_{12}. Time-lapse was conducted with a time interval of 1.13 seconds for 45 seconds.

Data S1. Source data of the similarities of Eut proteins between different bacterial species related to Figure S28 and the associated protein locus tags.
